# Global Distribution and Prevalence of Hepatitis C Virus Genotypes

**DOI:** 10.1002/hep.27259

**Published:** 2014-07-28

**Authors:** Jane P Messina, Isla Humphreys, Abraham Flaxman, Anthony Brown, Graham S Cooke, Oliver G Pybus, Eleanor Barnes

**Affiliations:** 1 Spatial Epidemiology and Ecology Group, Department of Zoology, University of OxfordOxford, UK; 2 Peter Medawar Building for Pathogen Research, University of Oxford, and Oxford NHIR BRCOxford, UK; 3 Institute of Health Metrics and EvaluationSeattle, WA, USA; 4 Division of Infectious Diseases, St Mary's Campus, Imperial CollegeLondon, UK; 5 Department of Zoology, University of OxfordOxford, UK

## Abstract

Hepatitis C virus (HCV) exhibits high genetic diversity, characterized by regional variations in genotype prevalence. This poses a challenge to the improved development of vaccines and pan-genotypic treatments, which require the consideration of global trends in HCV genotype prevalence. Here we provide the first comprehensive survey of these trends. To approximate national HCV genotype prevalence, studies published between 1989 and 2013 reporting HCV genotypes are reviewed and combined with overall HCV prevalence estimates from the Global Burden of Disease (GBD) project. We also generate regional and global genotype prevalence estimates, inferring data for countries lacking genotype information. We include 1,217 studies in our analysis, representing 117 countries and 90% of the global population. We calculate that HCV genotype 1 is the most prevalent worldwide, comprising 83.4 million cases (46.2% of all HCV cases), approximately one-third of which are in East Asia. Genotype 3 is the next most prevalent globally (54.3 million, 30.1%); genotypes 2, 4, and 6 are responsible for a total 22.8% of all cases; genotype 5 comprises the remaining <1%. While genotypes 1 and 3 dominate in most countries irrespective of economic status, the largest proportions of genotypes 4 and 5 are in lower-income countries. *Conclusion*: Although genotype 1 is most common worldwide, nongenotype 1 HCV cases—which are less well served by advances in vaccine and drug development—still comprise over half of all HCV cases. Relative genotype proportions are needed to inform healthcare models, which must be geographically tailored to specific countries or regions in order to improve access to new treatments. Genotype surveillance data are needed from many countries to improve estimates of unmet need. (Hepatology 2015;61:77–87)

Hepatitis C virus (HCV) is a globally prevalent pathogen and a leading cause of death and morbidity.^1^ The most recent estimates of disease burden show an increase in seroprevalence over the last 15 years to 2.8%, equating to >185 million infections worldwide.^2^ Persistent HCV infection is associated with the development of liver cirrhosis, hepatocellular cancer, liver failure, and death,^3^ and HCV is now the most common cause of death in HIV-positive patients on highly active antiretroviral therapy.^4^ While the incidence rate of HCV infection is apparently decreasing in the developed world, deaths from liver disease secondary to HCV infection will continue to increase over the next 20 years.^5^

Historically, HCV drug therapy has depended on interferon-α (administered by injection) and ribavirin over many months and is associated with severe side effects. The resources required for treating HCV patients with such drugs have been a considerable barrier for healthcare systems in many low and/or lower-middle income countries, despite treatment outcomes that are comparable to those in well-resourced settings.^6^–^8^ That said, the coming decade should witness a remarkable transformation in the treatment of HCV infection. While the first generation of new direct-acting antivirals (DAAs) were given in combination with interferon and ribavirin, and thus added to the burden of side effects,^9^,^10^ second-generation DAA therapies with minimal side effects and shortened courses of therapy are associated with cure rates of more than 90% in Phase II or III studies.^11^ Moreover, multiple DAA therapies targeting distinct HCV proteins have been developed and, when given in combination, will obviate the need for interferon treatment.^12^–^14^ Thus, if DAAs are made affordable, the treatment of HCV across the globe will become a realistic option for the first time.

HCV exhibits an extraordinarily high degree of genetic diversity—substantially greater than that of the HIV-1 pandemic—creating a major challenge for the development of both HCV vaccines and pan-genotypic drug therapies.^15^ At present, the duration of treatment, cure rates, and the need for adjuvant interferon and ribavirin with the new DAA therapies remain dependent in part on HCV genotype and subtype. Therefore, the development of national treatment strategies using DAA therapies requires a detailed understanding of relative HCV genotype prevalence and subtypes.

Furthermore, the first prophylactive T-cell vaccines that aim to prevent persistent HCV infection are currently in Phase II efficacy testing, with further candidates moving into human studies.^16^ The first generation of vaccines in development contain a subtype-1b immunogen in viral vectors that are deployed in a heterologous prime-boost regimen.^17^ In countries with mixed genotype infection, crossreactive immunity will depend on the generation of an immune response that targets HCV antigens that are conserved between genotypes. An alternative strategy, however, would be to develop geographically tailored vaccine immunogens for deployment at a country or regional level where detailed information on viral subtypes is available. Overall, the rational testing of HCV vaccine candidates will require a comprehensive country-level understanding of relative subtype prevalence.

HCV strains are classified into seven recognized genotypes (1-7) on the basis of phylogenetic and sequence analyses of whole viral genomes.^18^ HCV strains belonging to different genotypes differ at 30-35% of nucleotide sites. Within each genotype, HCV is further classified into 67 confirmed and 20 provisional subtypes. Strains that belong to the same subtype differ at <15% of nucleotide sites.^19^ The contemporary global geographic distribution of HCV genotypes is complex. It has already been established that a few subtypes—specifically 1a, 1b, 2a, and 3a—are widely distributed across the globe and account for a large proportion of HCV infections in high-income countries. These so-called “epidemic subtypes” are thought to have spread rapidly in the decades prior to the discovery of HCV by way of infected blood, blood products, injecting drug use, and other routes.^20^–^22^ Many other HCV subtypes are considered “endemic” strains; these are comparatively rare and have circulated for long periods of time in more restricted regions: endemic strains from genotypes 1 and 2 are primarily in West Africa, 3 in south Asia, 4 in Central Africa and the Middle East, 5 in Southern Africa, and 6 in South East Asia.^20^,^23^,^24^ To date, only one genotype 7 infection has been reported; it was isolated in Canada from a Central African immigrant.^25^ The global distribution of HCV genetic variation has likely been influenced by historical and contemporary trends in human migration. For example, strains from West Africa appear to have been transferred to the Americas by way of the trans-Atlantic slave trade.^26^

Here we are the first to estimate the relative prevalence of HCV genotypes globally by region and by country where data permitted. The data described provide a platform for the rational deployment and efficacy testing of new DAA therapies and vaccines for HCV.

## Materials and Methods

We searched PubMed (http://www.pubmed.gov) for all articles containing the terms “HCV” or “hepatitis c virus” and “genotype” or “subtype” published between 1989 (the year that HCV was discovered) and 2013. As a standardized genotype classification system was not present before 1994, we converted HCV genotypes or serotypes in articles published between 1989 and 1994 to the consensus nomenclature proposed by Simmonds et al.^18^ In many studies HCV cases were classified at the genotype level but not at the subtype level. If articles contained the same patient cohort then this cohort was only counted once. We recorded the number of cases of each HCV genotype identified along with the size of the study population. Some individuals tested positive for more than one genotype; in such cases each genotype identified was given its own record. We did not include genotype 7 in the analysis (see introduction).

We summed the total numbers of HCV cases attributable to each genotype across all studies for each country. This enabled a straightforward calculation of the relative proportion of HCV infections comprised by each genotype. We then computed relative genotype frequencies for each Global Burden of Disease (GBD) region as defined by the World Health Organization (WHO).^27^ This allowed us to combine our genotype frequency estimates with recently published GBD estimates of overall HCV seroprevalence to estimate the numbers of genotype-specific cases within countries and regions as well as globally. Countries for which we were unable to obtain HCV genotype prevalence data were excluded from calculations of regional proportions, although their populations were included in the total population size of each region when generating regional genotype prevalence estimates. Since clinical studies of new DAA therapies have routinely been stratified by genotype 1 subtype, we examined the relative frequency of genotype 1 cases that were confirmed as either subtype 1a or 1b.

Mapping of genotype frequencies by country and region was performed using ArcGIS 9.3 (http://www.esri.com). The diversity of HCV genotypes present in each country was computed using the Shannon Diversity Index,^28^ resulting in a mappable score for each country that is a function of the number and relative frequency of genotypes identified within it. We also further stratified genotype prevalence by national income according to WHO income categories.^29^

## Results

In total, data from 1,217 studies were included in the analysis. A complete reference list for these is provided in the Supporting Information. The 117 countries for which information on HCV genotypes was found represent 90% of the world's population and are listed in Table 1 by WHO GBD region, along with the number of studies for each country (the total number of virus samples by country is provided in Supporting Information Table S1). Unsurprisingly, Western Europe and North America had the greatest number of applicable studies. No studies meeting our search criteria were available for the Oceania GBD region (which does not include Australia), and the number of studies with HCV genotype information is relatively poor for the Caribbean, Central Latin America (outside of Mexico), and parts of Africa. Altogether, HCV genotype frequency information is available for ∼60% of the world's countries (79 out of 196), representing nearly 90% of the world's population. The three most populous countries with no HCV genotype information are Bangladesh, Malaysia, and North Korea.

**Table 1:** Number of Studies per Country for Those Countries Included in the Analysis; Grouped by GBD Region

Region/Country	Number of Studies	Region/Country	Number of Studies
**Andean Latin America**	3	**North Africa and Middle East**	95
Bolivia	1	Algeria	2
Peru	2	Egypt	19
**Australasia**	20	Iran (Islamic Republic of)	9
Australia	19	Iraq	3
New Zealand	1	Israel	21
**Caribbean**	4	Jordan	1
Cuba	2	Kuwait	2
Martinique	1	Lebanon	3
Suriname	1	Libyan Arab Jamahiriya	1
**Central Asia**	18	Morocco	2
Azerbaijan	1	Qatar	2
Georgia	5	Saudi Arabia	4
Mongolia	8	Syrian Arab Republic	2
Tajikistan	1	Tunisia	6
Turkmenistan	1	Turkey	15
Uzbekistan	2	United Arab Emirates	3
**Central Europe**	43	**South Asia**	54
Albania	1	Afghanistan	1
Bulgaria	3	India	15
Croatia	6	Nepal	19
Czech Republic	1	Pakistan	19
Hungary	7	**Southeast Asia**	32
Montenegro	1	Brunei Darussalam	1
Poland	12	Cambodia	1
Romania	5	Indonesia	4
Slovakia	1	Lao People's Democratic Republic	2
Slovenia	5	Myanmar	2
The former Yugoslav Republic of Macedonia	1	Philippines	1
**Central Latin America**	29	Singapore	2
Colombia	3	Sri Lanka	1
Mexico	22	Thailand	11
Venezuela	4	Viet Nam	7
**Central sub-Saharan Africa**	7	**Southern Latin America**	42
Central African Republic	2	Argentina	31
Congo	1	Chile	5
Democratic Republic of the Congo	1	Uruguay	6
Equatorial Guinea	1	**Southern sub-Saharan Africa**	6
Gabon	2	Namibia	1
**East Asia**	35	South Africa	5
China	35	**Tropical Latin America**	73
**Eastern Europe**	19	Brazil	73
Belarus	2	**Western Europe**	560
Estonia	2	Austria	38
Latvia	3	Belgium	19
Lithuania	3	Cyprus	2
Republic of Moldova	2	Denmark	10
Russian Federation	6	Finland	4
Ukraine	1	France	111
**Eastern sub-Saharan Africa**	9	Germany	62
Eritrea	1	Greece	32
Ethiopia	1	Iceland	1
Kenya	1	Ireland	9
Madagascar	1	Italy	155
Mozambique	1	Luxembourg	1
Sudan	1	Netherlands	1
Uganda	1	Norway	6
United Republic of Tanzania	2	Portugal	4
**High-income Asia Pacific**	46	Spain	55
Japan	42	Sweden	19
Korea, Republic of	4	Switzerland	14
**High-income North America**	117	U.K. of Great Britain and Northern Ireland	17
Canada	32	**Western sub-Saharan Africa**	18
United States of America	85	Benin	1
**Middle East and North Africa**	1	Burkina Faso	2
Bahrain	1	Cameroon	3
		Cote d'Ivoire	1
		Gambia	2
		Ghana	1
		Guinea	2
		Guinea-Bissau	2
		Nigeria	4

**Table 2:** Global and Regional Estimates of HCV Seroprevalence Attributable to Each Genotype

	Genotype 1		Genotype 2		Genotype 3		Genotype 4		Genotype 5		Genotype 6		Regional HCV
WHO GBD Region	N (thousands)	%	N (thousands)	%	N (thousands)	%	N (thousands)	%	N (thousands)	%	N (thousands)	%	Seroprevalence Totals* (thousands)
Andean Latin America	1,003	90.9	17	1.5	83	7.6	0	0.0	0	0.0	0	0.0	1,103
Australasia	388	54.2	34	4.7	280	39.2	9	1.3	0	0.0	3	0.5	715
Caribbean	450	92.6	15	3.2	17	3.5	4	0.8	0	0.0	0	0.0	486
Central Asia	2,100	66.6	148	4.7	906	28.7	0	0.0	0	0.0	0	0.0	3,155
Central Europe	1,548	89.2	1	0.1	164	9.4	22	1.3	0	0.0	0	0.0	1,736
Central Latin America	2,796	71.7	754	19.3	330	8.5	16	0.4	2	0.0	0	0.0	3,899
Central sub-Saharan Africa	37	1.7	17	0.8	0	0.0	2,145	97.6	0	0.0	0	0.0	2,198
East Asia	32,082	58.0	8,444	15.3	5,762	10.4	40	0.1	0	0.0	8,982	16.2	55,311
Eastern Europe	4,023	65.1	270	4.4	1,881	30.4	6	0.1	0	0.0	0	0.0	6,181
Eastern sub-Saharan Africa	1,187	37.3	294	9.2	288	9.1	978	30.7	436	13.7	0	0.0	3,183
High-income Asia Pacific	1,926	74.9	629	24.5	15	0.6	0	0.0	0	0.0	0	0.0	2,571
High-income North America	3,595	75.8	567	12.0	492	10.4	55	1.2	6	0.1	26	0.6	4,742
North Africa and Middle East	3,808	27.3	115	0.8	884	6.3	9,118	65.3	47	0.3	0	0.0	13,971
South Asia	12,889	23.2	1,333	2.4	39,706	71.6	1,413	2.5	80	0.1	55	0.1	55,475
Southeast Asia	4,910	57.0	1,572	18.2	1,331	15.4	77	0.9	0	0.0	729	8.5	8,619
Southern Latin America	876	87.0	58	5.7	65	6.5	5	0.5	4	0.4	0	0.0	1,008
Southern sub-Saharan Africa	399	26.5	18	1.2	107	7.1	98	6.5	887	58.8	0	0.0	1,508
Tropical Latin America	1,802	69.3	89	3.4	699	26.9	7	0.3	3	0.1	0	0.0	2,600
Western Europe	3,169	59.0	583	10.8	1,332	24.8	262	4.9	26	0.5	2	0.0	5,374
Western sub-Saharan Africa	4,427	65.7	1,550	23.0	0	0.0	761	11.3	5	0.1	0	0.0	6,743
Totals (excludes Oceania)	*83,413.4*	*46.2*	*16,509.0*	*9.1*	*54,345.0*	*30.1*	*15,014.5*	*8.3*	*1,496.3*	*0.8*	*9,798.6*	*5.4*	*180,576.8*

* Regional HCV seroprevalence data from Hanafiah et al.^2^

**Fig. 1:**
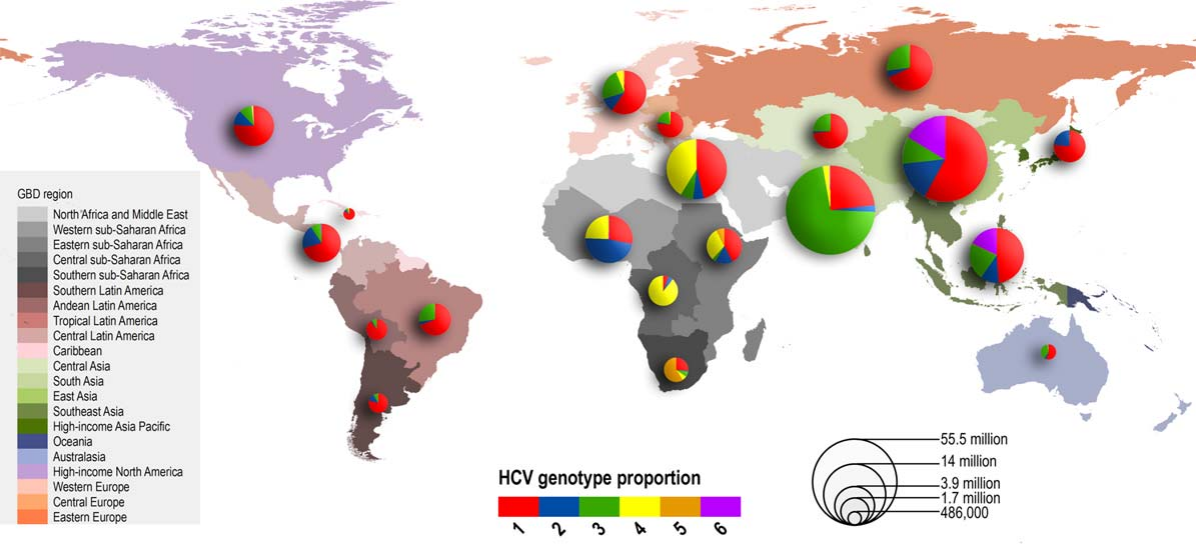
Relative prevalence of each HCV genotype by GBD region. Size of pie charts is proportional to the number of seroprevalent cases as estimated by Hanafiah et al.^2^

The relative prevalence of each HCV genotype by GBD region is mapped using pie charts in Fig. 1 and numerically in Table 2; the size of each chart is proportional to the number of regional seroprevalent HCV cases as estimated by Hanafiah et al.^2^ A complete breakdown of HCV genotype frequency information by country is provided in the Supporting Information (Table S1). Globally, genotype 1 is estimated to account for more HCV cases than any other genotype at 83.4 million (46.2%), with over one-third of genotype 1 cases located in East Asia. HCV genotype 3 is the next most common and is estimated to account for 54.3 million (30.1%) cases globally, approximately three-quarters of which occur in south Asia. Genotypes 2, 4, and 6 are responsible for the majority of the remaining cases of HCV worldwide, with an estimated 16.5 million (9.1%), 15.0 million (8.3%), and 9.8 million (5.4%) cases, respectively. East Asia accounts for the greatest numbers of genotype 2 and genotype 6 HCV cases, while North Africa and the Middle East have the largest number of genotype 4 cases. We estimate genotype 5 to be responsible for the fewest HCV cases globally (1.4 million, <1% of all HCV cases), the great majority of which occur in Southern and Eastern sub-Saharan Africa.

**Fig. 2:**
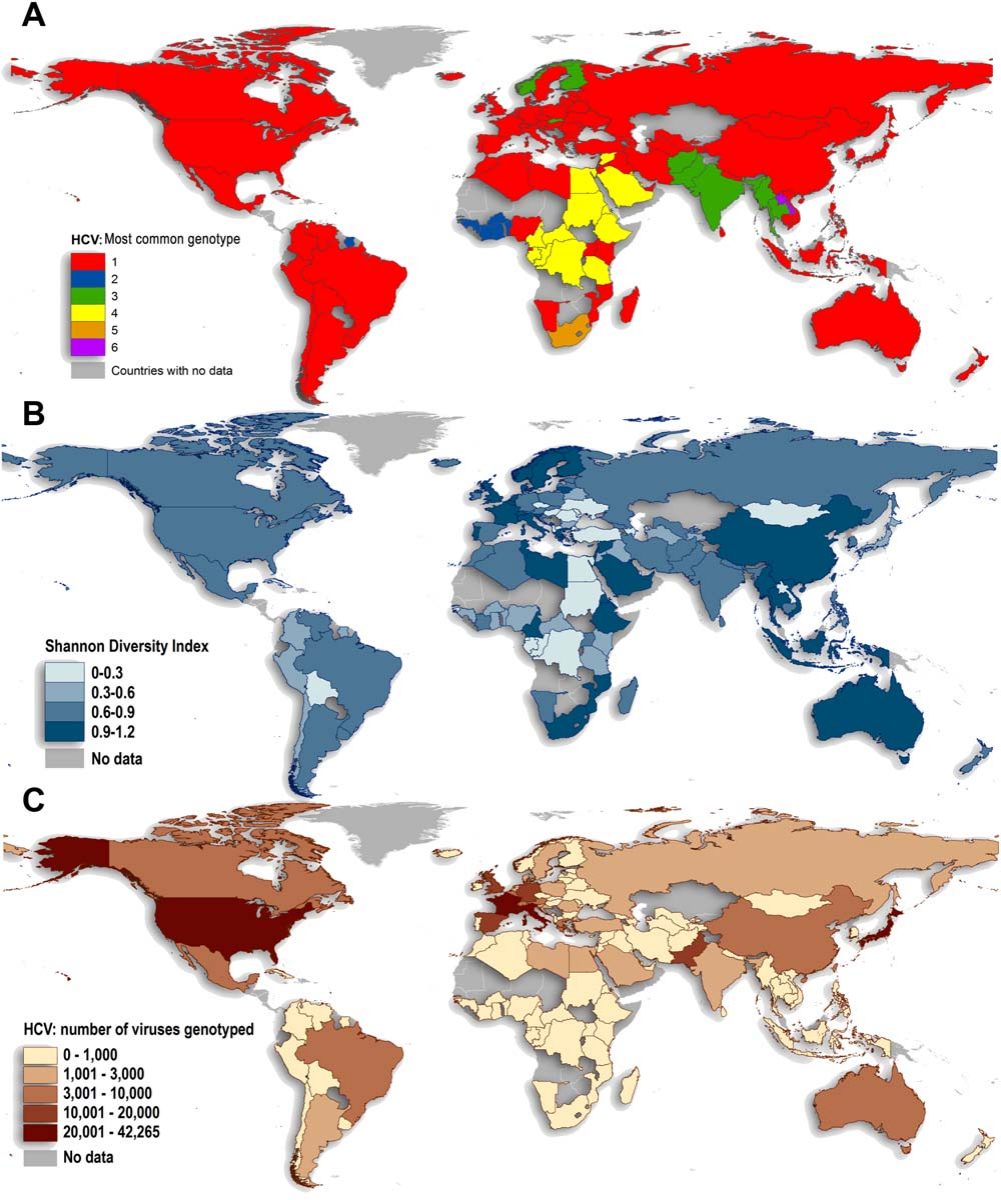
(A) Countries by the majority genotype found across all associated studies. (B) Shannon Diversity Index for study countries, ranging from 0 (comprised of larger proportions of a few genotypes) to 1.2 (comprised of smaller proportions of many genotypes). (C) Number of genotyped virus samples across all studies by country.

Figure 2A displays the most common genotype by country. Genotype 1 is the most common genotype in 85 of the 117 countries identified as having genotype information in our study, and is highly prevalent worldwide. Of the 53% of genotype 1 cases for which the subtype was specified, we find 99% to be attributable to subtypes 1a and 1b (31% and 68%, respectively). A map showing the dominance of subtype 1a versus 1b in countries whose relative prevalence of genotype 1 exceeds 25% is provided in the Supporting Information (Fig. S5). We observed that genotype 2 dominated in West Africa, genotype 3 in south Asia and parts of Scandinavia, genotype 4 in Central and North Africa, Genotype 5 in South Africa, and genotype 6 in SE Asia.

The Shannon Diversity Index computed for each country is mapped in Fig. 2B. A low score indicates that most infections belong to one or two dominant genotypes, whereas a high score indicates that infections are more evenly distributed across several genotypes. The diversity of HCV genotypes varies considerably across countries, with few discernable geographic patterns. Diversity is high in China and many Southeast Asian countries, and also in Western Europe and Australia.

The absolute number of genotyped cases, and the numbers genotyped as a proportion of the population included in our survey, is shown for each country in Table S1 and Fig. 2C. Large sample sizes were available for the USA and many countries in Western Europe and Japan, whereas South America, Africa, parts of Eastern Europe, and Asia were relatively poorly sampled.

**Fig. 3:**
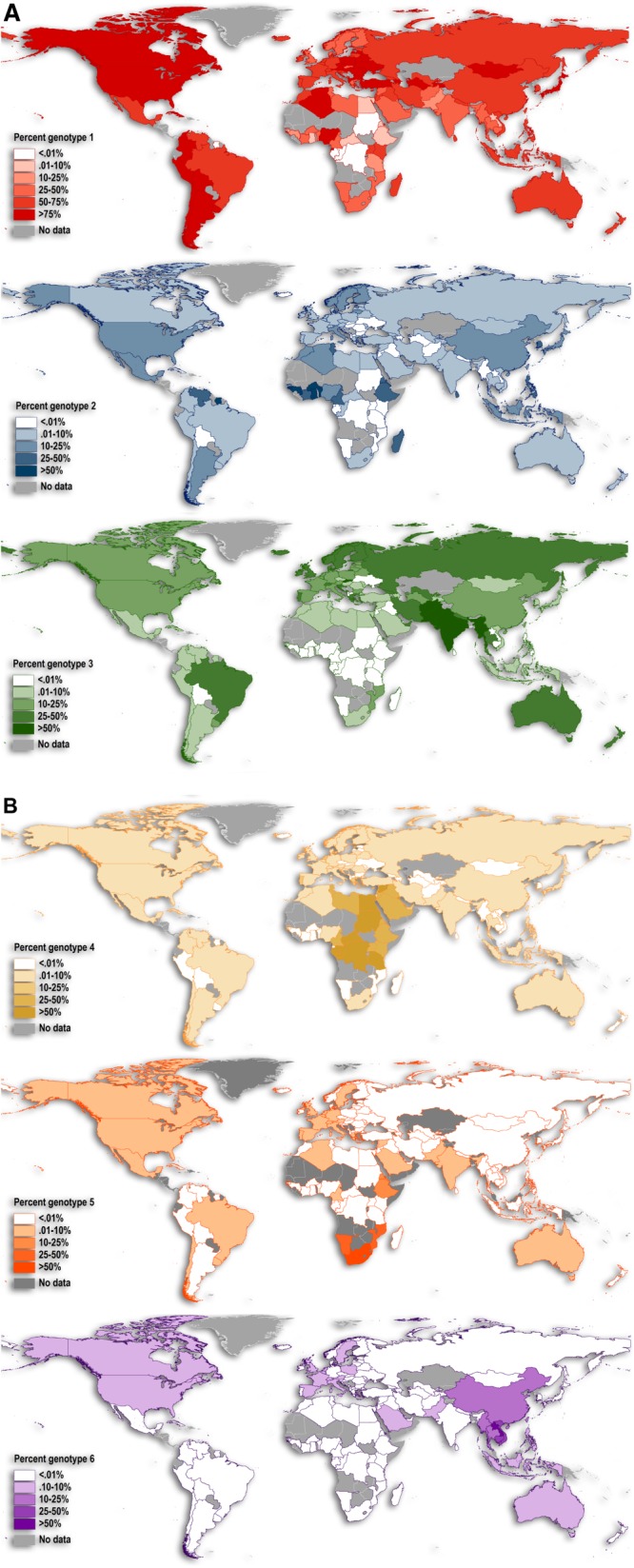
Relative prevalence of each genotype across all virus samples by country. Panel A: genotypes 1–3.

Figure 3A,B displays six maps (one for each genotype) of the relative prevalence among all HCV genotypes by country. This figure reinforces the patterns seen in Fig. 2A for genotype 1, displaying its wide geographic extent and overall high prevalence worldwide. The frequency of genotype 3 cases is notably higher in south Asia but is comparatively lower in Africa (Fig. 3A). Genotypes 2, 4, 5, and 6 are typically more restricted in their extents. Genotype 4 frequencies are highest from central Africa to the Middle East, while genotype 5 only reaches higher frequencies in southern Africa (Fig. 3B). Lastly, genotype 6 is present at the highest frequencies in East and Southeast Asia, but is the dominant genotype in only one country—Laos (see Figs. 2A and 3B); it is also prevalent in neighboring Vietnam. These patterns are broadly consistent with previous informal summaries of the geographic structure of HCV genetic diversity.^23^,^30^,^31^

HCV cases in countries classified as high-income by the WHO were comprised of 66% of genotype 1, 18% genotype 3, 12% genotype 2, and small proportions of genotypes 4-6. Upper-middle income countries displayed a similar composition of HCV genotypes, although with a greater proportion of genotype 6 cases (11%). In contrast, the low and lower-middle income countries displayed relatively high proportions of genotypes 4 and 5. For the genotype proportions for all income classes, see the Supporting Information (Table S2).

## Discussion

The data presented here represent the most comprehensive effort to date to estimate the relative contribution of each HCV genotype to the global HCV epidemic. Such data are crucial to inform future prevention efforts (including vaccine design) as well as policies to ensure global access to the new generation of interferon-free DAAs. Aside from countries entirely lacking genotype information, our data highlight that the evidence base for genotype prevalence is weakest in Sudan, the Democratic Republic of the Congo, and Tanzania, in terms of the number of viruses genotyped as a proportion of their overall populations (Table S1; Fig. 2C). Of the world's countries for which genotype data are missing, the largest populations are in Asia (accounting for 3.6% of the global population), followed by Africa (3.2% of the global population) and Latin America (1.4% of the global population). These regions typically represent areas of health neglect, and should be a priority for further surveillance as disease burden attributable to HCV is also likely to be high in these locations.

It is important to note that the most prevalent genotype in developed economies (genotype 1) is also the most prevalent globally and should be well served by interferon-free regimens of second-generation DAA therapies with viral eradication rates of >90%.^13^,^32^ Genotype 3, however, which accounts for ∼30% of global infections, is not susceptible to the first generation of DAA protease inhibitors, and also appears less susceptible than other genotypes to sofosbuvir, the most advanced second-generation DAA therapy to date.^33^,^34^ Ultimately, should DAA therapies currently in development prove to be equally effective against all viral genotypes, viral genotyping will cease to be relevant to therapy. Even so, early data suggest that potential pan-genotypic combinations in late-stage development will continue to require stratification by viral genotype to define the optimal duration of drug therapy (e.g., sofosbuvir/GS5816 or MK5172/8472).^35^

One possible hypothesis for the current global distribution of genotype 1 is the chance association of subtypes 1a and 1b with the international dissemination of contaminated blood and blood products during the twentieth century, prior to the discovery of HCV in 1989.^22^ The global dissemination of genotype 3 is likely due to the association of subtype 3a with injection drug use^21^,^36^,^37^ and to population migration from countries where subtype 3a is dominant, such as India and Pakistan.^38^

Although HCV genotype 1 and 3 infections are more prevalent than all other genotypes globally, genotypes found more commonly in lower-income countries still account for a significant proportion of HCV cases worldwide (Table S2). Specifically, genotype 2 is most frequent in West Africa and parts of South America (the latter likely reflecting population movements resulting from the trans-Atlantic slave trade),^26^ while genotypes 4 and 6 are common in Central/North Africa and East/Southeast Asia, respectively. We estimate that genotypes 2, 4, and 6 combined account for nearly one-quarter of all HCV cases globally. Although initial data suggest that genotype 2 will be relatively straightforward to treat in DAA interferon-free regimens,^33^,^34^ the need for more data about genotype 4 and 6 infections is a priority in order to inform local policies, especially in areas of highest prevalence.

Stratification by viral genotype at a national and regional level, and a better understanding of viral diversity within target populations, will also critically inform the rational design and testing of HCV vaccines. We have shown that diversity tends to be high in high-prevalence countries. The first T-cell vaccine, which hosts a genotype-1 immunogen, is currently undergoing efficacy testing in intravenous-drug user (IVDU) populations in Baltimore and San Francisco, where genotype 1 infection is dominant. Phase I clinical data suggest that the generation of T cells that target multiple viral genotypes using this vaccine is possible.^17^ Still, the roll-out of this and other HCV vaccines to other countries will require a detailed understanding of HCV genetic diversity within new target populations. Genotype diversity is particularly high in China and many Southeast Asian countries, and also in Western Europe and Australia, perhaps as a result of population immigration from Africa and/or Asia.^39^ In countries like these, vaccine efficacy at a population level would be dependent on the generation of crossreactive immunity; an alternative approach could also be the development of vaccines hosting different immunogens. In contrast, in countries where diversity is constrained such as Egypt (low diversity here is likely the result of the rapid amplification of subtype 4a during the twentieth century as a result of specific iatrogenic events),^40^ vaccine immunogens designed to most closely align with population HCV sequence may be the optimal strategy.

Our study is intended as a comprehensive literature survey and is not intended as a statistically formal meta-analysis. PubMed is the world's largest medical library, and so we are confident in the comprehensiveness of our published literature base. However, we recognize that there are limitations within our dataset, and also that there may be some useful unpublished reports that were excluded from this study. Foremost among these is potential selection bias, since not all studies included were surveys of the general population (or proxies thereof, such as prospective blood donors). Thus, some risk groups associated with specific genotypes may be more readily sampled than others, and the impact of this may be strongest in those countries with the fewest number of genotyped cases. A mechanism for removing studies of certain risk groups would be required, and such a subjective decision could lead to additional sources of bias. In addition, we have not stratified studies according to the methodology used to genotype patients. Since the HCV RNA 5′UTR (untranslated region) is the most common target for diagnostic HCV RNA assays, it is also the viral region most commonly used to define viral genotype. While this region is relatively conserved, single nucleotide differences assessed using sequencing or probe-based assays are most commonly used in assays to define viral genotype.^41^ Importantly, these assays have been deemed adequate for current clinical applications. Although clinical assays that assess viral subtype also rely on 5′UTR as a substrate, the accuracy of HCV subtyping will be improved by additional sequencing of other viral regions such as core^42^ and NS5B.^43^ Overall, we believe errors arising from the misassignment of viral genotype to be limited. As a consequence of all the above, we have chosen not to provide confidence limits for our prevalence estimates, as we believe any such measure of uncertainty would be inappropriate without an explicit statistical sampling model. This is an important task for future refinements of the values reported here. Lastly, we have not addressed the contribution of each HCV genotype to the overall burden of morbidity and mortality due to acute infection, liver failure, or cirrhosis.

Our estimates of relative genotype prevalence are dependent on viral genetic data in association with global and national estimates of HCV prevalence based on seropositivity, which includes instances of cleared infections.^1^ Our estimates of relative genotype prevalence could be affected, therefore, if one genotype is cleared more readily than another. Comparison of spontaneous clearance rates between genotypes is unreliable since these have largely relied on the assessment of outbreak studies of single genotypes in distinct patient populations^44^ and are therefore subject to cohort bias. Consequently, the absolute number of cases attributable to each genotype reported here is most correctly interpreted as the number of infections of each genotype—past and present—that induced the seropositivity observed. Thus, if a country has undergone rapid, recent, and significant changes in relative genotype frequency, our absolute counts may be a poor proxy for the genotypes that characterize future incidence.

Although genotypes 1 and 3 account for the majority of infections worldwide, HCV strains belonging to the rarer genotypes 2, 4, 5, and 6 can increase rapidly in prevalence if they happen to become associated with efficient routes of transmission. The high prevalence and dominance of subtype 4a in Egypt is widely believed to have resulted from its spread by way of unsafe injections during past anti-schistosomal public health campaigns.^40^ Other subtypes have been amplified locally and regionally after being introduced into networks of injection drug users—for example, subtype 4d in Europe^45^,^46^ and subtype 6a in Vietnam and Hong Kong.^47^ These observations suggest that under the right circumstances, most, if not all, HCV genotypes have epidemic potential. They also suggest that social, behavioral, and demographic factors (including international migration) are more important than viral genetic variation in determining the global prevalence of different genotypes.

## Abbreviations

DAA: direct-acting antiviral
GBD: Global Burden of Disease project
HCV: hepatitis C virus
IVDU: intravenous drug user
WHO: World Health Organization
