# Supplementary material for: Global Distribution and Prevalence of Hepatitis C Virus Genotypes
Source: Hepatology. 2014 Jul 28;61(1):77–87. doi: 10.1002/hep.27259 (PMC4303918; doi:10.1002/hep.27259)
Supplement: Supplementary file 1 — Supplementary Information [file hep0061-0077-sd1.pdf]

Supplementary Information:

The global distribution of HCV genotypes

Jane P. Messina[1], Isla Humphreys[2], Abraham Flaxman[3], Anthony Brown[2] Graham S. Cooke[4], Oliver G. Pybus[5], Eleanor Barnes[2]

[1] Spatial Epidemiology and Ecology Group, Department of Zoology, University of Oxford, Oxford, UK
[2] Peter Medawar Building for Pathogen Research, University of Oxford, Oxford,UK
[3] Institute of Health Metrics and Evaluation, Seattle, WA, USA
[4] Division of Infectious Diseases, St Mary's Campus, Imperial College, London, UK
[5] Department of Zoology, University of Oxford, South Parks Road, Oxford, UK

Contact Email: jane.messina@zoo.ox.ac.uk

Table S1

Estimated genotype-specific prevalence of HCV by country

Country	Genotype 1		Genotype 2		Genotype 3		Genotype 4		Genotype 5		Genotype 6		Total Viruses	Total Cases
	N (thousands)	*%*	*N (thousands)*	*%*	*N (thousands)*	*%*	*N (thousands)*	*%*	*N (thousands)*	*%*	*N (thousands)*	*%*	*per 10,000 pop.*	*(thousands)*
Afghanistan	10	38.03	0	0.00	17	61.97	0	0.00	0	0.00	0	0.00	25	27
Albania	1	100.00	0	0.00	0	0.00	0	0.00	0	0.00	0	0.00	10	1
Algeria	34	82.35	5	11.43	1	3.51	1	2.38	0	0.34	0	0.00	236	42
Argentina	59	76.31	12	15.22	6	7.80	1	0.67	0	0.00	0	0.00	263	77
Australia	244	54.23	21	4.71	176	39.18	6	1.35	0	0.03	2	0.50	1,524	449
Austria	13	77.75	0	2.43	3	14.42	1	5.35	0	0.02	0	0.02	4,949	17
Azerbaijan	43	93.75	0	0.00	3	6.25	0	0.00	0	0.00	0	0.00	53	45
Bahrain	1	66.67	0	33.33	0	0.00	0	0.00	0	0.00	0	0.00	24	1
Belarus	35	81.22	0	0.51	7	17.26	0	1.02	0	0.00	0	0.00	208	43
Belgium	117	66.23	8	4.41	33	18.72	13	7.39	6	3.19	0	0.07	4,048	177
Benin	4	28.57	9	71.43	0	0.00	0	0.00	0	0.00	0	0.00	7	12
Bolivia	18	100.00	0	0.00	0	0.00	0	0.00	0	0.00	0	0.00	7	18
Brazil	1,240	69.29	61	3.43	481	26.87	5	0.28	2	0.12	0	0.00	333	1,790
Brunei Darussalam	0	33.33	0	16.67	0	50.00	0	0.00	0	0.00	0	0.00	175	0
Bulgaria	98	87.77	0	0.00	14	12.23	0	0.00	0	0.00	0	0.00	188	112
Burkina Faso	6	28.57	15	71.43	0	0.00	0	0.00	0	0.00	0	0.00	14	22
Cambodia	10	50.00	0	1.72	0	0.00	0	0.00	0	0.00	9	48.28	40	19
Cameroon	214	25.74	223	26.84	0	0.00	391	47.06	3	0.37	0	0.00	132	831
Canada	238	79.26	19	6.35	32	10.72	7	2.22	3	1.06	1	0.40	886	301
Central African Republic	1	8.43	1	7.23	0	0.00	9	84.34	0	0.00	0	0.00	191	10
Chile	170	91.67	4	1.89	11	5.68	1	0.38	1	0.38	0	0.00	154	185
China	10,107	58.00	2,660	15.27	1,815	10.42	13	0.07	0	0.00	2,830	16.24	41	17,425
Colombia	66	87.76	5	6.12	2	2.04	3	4.08	0	0.00	0	0.00	11	75
Congo	0	0.00	0	4.55	0	0.00	8	95.45	0	0.00	0	0.00	54	8
Cote d'Ivoire	14	40.00	20	60.00	0	0.00	0	0.00	0	0.00	0	0.00	3	34
Croatia	7	60.53	0	1.67	4	35.11	0	2.69	0	0.00	0	0.00	3,857	12

Cuba	32	95.97	0	0.00	1	3.23	0	0.81	0	0.00	0	0.00	110	34
Cyprus	1	77.19	0	3.51	0	7.02	0	10.53	0	1.75	0	0.00	516	2
Czech Republic	25	95.60	0	1.10	1	3.30	0	0.00	0	0.00	0	0.00	86	26
Democratic Republic of the Congo	0	0.00	0	0.00	0	0.00	119	100.00	0	0.00	0	0.00	1	119
Denmark	24	46.93	4	8.37	22	41.91	1	2.79	0	0.00	0	0.00	4,196	52
Egypt	164	2.73	16	0.26	9	0.15	5,834	96.86	0	0.00	0	0.00	343	6,023
Equatorial Guinea	2	100.00	0	0.00	0	0.00	0	0.00	0	0.00	0	0.00	14	2
Eritrea	0	6.25	3	37.50	0	0.00	3	43.75	1	12.50	0	0.00	30	7
Estonia	4	73.38	0	3.80	1	22.81	0	0.00	0	0.00	0	0.00	2,025	6
Ethiopia	7	5.56	42	33.33	0	0.00	63	50.00	14	11.11	0	0.00	2	127
Finland	4	37.72	2	16.65	5	45.52	0	0.12	0	0.00	0	0.00	1,600	11
France	646	59.31	96	8.78	229	21.05	97	8.93	20	1.85	1	0.07	4,232	1,088
Gabon	0	0.00	0	0.00	0	0.00	4	100.00	0	0.00	0	0.00	135	4
Gambia	0	0.00	22	100.00	0	0.00	0	0.00	0	0.00	0	0.00	36	22
Georgia	19	60.43	0	0.40	13	39.17	0	0.00	0	0.00	0	0.00	1,704	32
Germany	1,055	71.63	83	5.63	300	20.37	34	2.34	0	0.03	0	0.00	1,601	1,473
Ghana	39	12.50	276	87.50	0	0.00	0	0.00	0	0.00	0	0.00	14	315
Greece	69	42.36	12	7.65	56	34.46	24	15.05	1	0.47	0	0.00	3,635	162
Guinea	5	27.27	12	72.73	0	0.00	0	0.00	0	0.00	0	0.00	14	17
Guinea-Bissau	0	0.88	3	99.12	0	0.00	0	0.00	0	0.00	0	0.00	712	3
Hungary	26	98.74	0	0.00	0	0.31	0	0.94	0	0.00	0	0.00	319	26
Iceland	0	61.54	0	0.00	0	38.46	0	0.00	0	0.00	0	0.00	1,226	0
India	4,446	29.62	277	1.84	10,141	67.58	133	0.89	10	0.07	0	0.00	12	15,006
Indonesia	1,755	66.97	557	21.27	249	9.50	47	1.81	0	0.00	12	0.45	9	2,620
Iran (Islamic Republic of)	827	57.66	9	0.60	559	39.00	39	2.75	0	0.00	0	0.00	112	1,434
Iraq	195	71.70	0	0.00	0	0.00	77	28.30	0	0.00	0	0.00	17	273
Ireland	5	58.81	0	2.24	3	38.46	0	0.48	0	0.00	0	0.00	1,397	8
Israel	8	71.37	2	14.10	1	9.19	1	4.91	0	0.00	0	0.43	1,265	11
Italy	1,189	62.87	405	21.42	232	12.27	64	3.37	1	0.03	1	0.03	5,256	1,891
Japan	1,096	76.64	332	23.19	2	0.15	0	0.02	0	0.00	0	0.00	1,590	1,430
Jordan	18	73.33	0	0.00	0	0.00	7	26.67	0	0.00	0	0.00	46	25
Kenya	44	73.08	0	0.00	0	0.00	16	26.92	0	0.00	0	0.00	6	60
Korea, Republic of	382	70.41	151	27.84	10	1.76	0	0.00	0	0.00	0	0.00	153	543

Kuwait	1	23.70	0	0.00	1	20.00	2	56.30	0	0.00	0	0.00	451	4
Lao People's Democratic Republic	4	4.76	0	0.00	0	0.00	0	0.00	0	0.00	79	95.24	66	82
Latvia	7	79.17	0	4.17	2	16.67	0	0.00	0	0.00	0	0.00	344	9
Lebanon	3	37.82	0	0.84	2	27.73	2	33.61	0	0.00	0	0.00	274	7
Libyan Arab Jamahiriya	3	43.87	1	8.12	1	9.09	3	38.91	0	0.00	0	0.00	3,773	7
Lithuania	9	63.39	2	12.02	3	24.04	0	0.55	0	0.00	0	0.00	596	14
Luxembourg	0	53.37	0	4.74	0	32.92	0	8.35	0	0.62	0	0.00	15,787	1
Madagascar	15	52.94	13	47.06	0	0.00	0	0.00	0	0.00	0	0.00	8	27
Martinique	0	81.19	0	6.87	0	7.76	0	3.58	0	0.30	0	0.30	8,354	0
Mexico	638	70.47	180	19.91	84	9.29	3	0.28	1	0.06	0	0.00	7,519	906
Mongolia	95	97.68	2	1.86	0	0.46	0	0.00	0	0.00	0	0.00	1,589	97
Montenegro	1	63.33	0	4.00	0	25.33	0	7.33	0	0.00	0	0.00	2,419	1
Morocco	32	74.34	11	24.25	0	0.53	0	0.88	0	0.00	0	0.00	181	43
Mozambique	127	50.00	0	0.00	56	22.22	0	0.00	71	27.78	0	0.00	8	254
Myanmar	25	29.55	0	0.00	45	53.03	0	0.00	0	0.00	15	17.42	25	86
Namibia	2	50.00	0	0.00	0	0.00	0	0.00	2	50.00	0	0.00	9	4
Nepal	277	52.50	0	0.00	250	47.50	0	0.00	0	0.00	0	0.00	15	527
Netherlands	149	53.31	24	8.40	69	24.77	37	13.35	0	0.11	0	0.05	1,119	280
New Zealand	5	53.13	1	6.25	4	40.63	0	0.00	0	0.00	0	0.00	73	10
Nigeria	2,448	82.28	452	15.19	0	0.00	75	2.53	0	0.00	0	0.00	5	2,975
Norway	3	39.29	1	10.55	4	49.51	0	0.65	0	0.00	0	0.00	1,259	9
Pakistan	1,177	11.93	334	3.39	7,793	78.95	514	5.21	27	0.27	25	0.26	815	9,870
Peru	59	88.46	1	1.92	6	9.62	0	0.00	0	0.00	0	0.00	18	66
Philippines	761	73.20	274	26.35	0	0.00	2	0.23	0	0.00	2	0.23	48	1,040
Poland	419	78.11	0	0.06	101	18.76	16	3.00	0	0.00	0	0.06	419	536
Portugal	10	51.20	2	9.34	5	26.20	3	12.95	0	0.30	0	0.00	314	20
Qatar	0	0.00	0	0.00	0	0.00	1	100.00	0	0.00	0	0.00	1,749	1
Republic of Moldova	13	94.74	0	1.75	0	3.51	0	0.00	0	0.00	0	0.00	174	13
Romania	645	99.75	0	0.00	1	0.13	1	0.13	0	0.00	0	0.00	1,085	646
Russian Federation	1,977	62.53	148	4.67	1,034	32.70	3	0.09	0	0.00	0	0.00	78	3,161
Saudi Arabia	102	40.08	13	5.22	15	5.88	122	47.92	2	0.85	0	0.05	732	254
Singapore	4	81.03	0	6.90	1	12.07	0	0.00	0	0.00	0	0.00	114	5
Slovakia	5	39.13	0	0.00	8	60.87	0	0.00	0	0.00	0	0.00	169	14

Slovenia	3	56.75	0	5.72	2	36.22	0	1.31	0	0.00	0	0.00	9,275	5
South Africa	200	26.33	9	1.18	54	7.10	49	6.51	448	58.88	0	0.00	66	760
Spain	65	71.81	2	2.25	17	18.28	7	7.20	0	0.44	0	0.02	2,866	91
Sri Lanka	183	55.56	146	44.44	0	0.00	0	0.00	0	0.00	0	0.00	13	329
Sudan	0	0.00	0	0.00	0	0.00	74	100.00	0	0.00	0	0.00	1	74
Suriname	0	0.00	1	90.00	0	10.00	0	0.00	0	0.00	0	0.00	190	1
Sweden	9	42.44	5	22.83	8	33.60	0	0.85	0	0.23	0	0.06	1,882	22
Switzerland	9	51.12	2	9.37	5	30.61	1	8.67	0	0.11	0	0.11	4,566	17
Syrian Arab Republic	91	29.35	2	0.76	5	1.51	182	58.70	30	9.68	0	0.00	307	310
Tajikistan	19	85.42	1	6.25	2	8.33	0	0.00	0	0.00	0	0.00	63	22
Thailand	439	31.10	60	4.24	583	41.36	2	0.11	0	0.00	327	23.19	135	1,411
The former Yugoslav Republic of Macedonia	4	100.00	0	0.00	0	0.00	0	0.00	0	0.00	0	0.00	252	4
Tunisia	6	40.63	4	27.81	1	4.38	4	27.19	0	0.00	0	0.00	304	16
Turkey	1,151	94.68	17	1.41	16	1.35	31	2.57	0	0.00	0	0.00	227	1,215
Turkmenistan	16	81.25	0	0.00	4	18.75	0	0.00	0	0.00	0	0.00	34	19
U.K. of Great Britain and Northern Ireland	737	44.83	110	6.69	735	44.71	56	3.39	5	0.31	1	0.07	3,104	1,643
Uganda	27	64.71	0	0.00	0	0.00	15	35.29	0	0.00	0	0.00	5	41
Ukraine	197	100.00	0	0.00	0	0.00	0	0.00	0	0.00	0	0.00	7	197
United Arab Emirates	3	49.57	0	4.27	2	25.21	1	20.94	0	0.00	0	0.00	278	6
United Republic of Tanzania	13	22.22	0	0.00	0	0.00	45	77.78	0	0.00	0	0.00	2	58
United States of America	2,570	75.52	424	12.46	352	10.36	36	1.06	1	0.04	19	0.57	1,354	3,403
Uruguay	6	78.85	0	3.85	1	13.46	0	0.00	0	3.85	0	0.00	154	7
Uzbekistan	405	59.59	39	5.70	236	34.72	0	0.00	0	0.00	0	0.00	70	680
Venezuela	35	68.17	14	28.23	2	3.30	0	0.30	0	0.00	0	0.00	115	51
Viet Nam	86	60.56	8	5.90	7	4.97	0	0.00	0	0.00	41	28.57	36	142

Table S2

Genotype proportions for WHO income categories

Income grouping	HCV genotype proportions by WHO income grouping (% of all genotypes)					
	1	2	3	4	5	6
High-Income	65.9	11.7	18.0	4.0	0.3	0.2
Low-Income	40.3	8.3	25.4	18.5	5.9	1.7
Lower Middle-Income	30.7	6.7	44.4	17.7	0.2	0.4
Upper Middle-Income	60.6	10.8	15.9	0.6	1.5	10.7

Figures S1-S4

The relative prevalence of each HCV genotype by country displayed by region. Pie charts are uniform in size.

Figure S1: Africa & the Arabian Peninsula

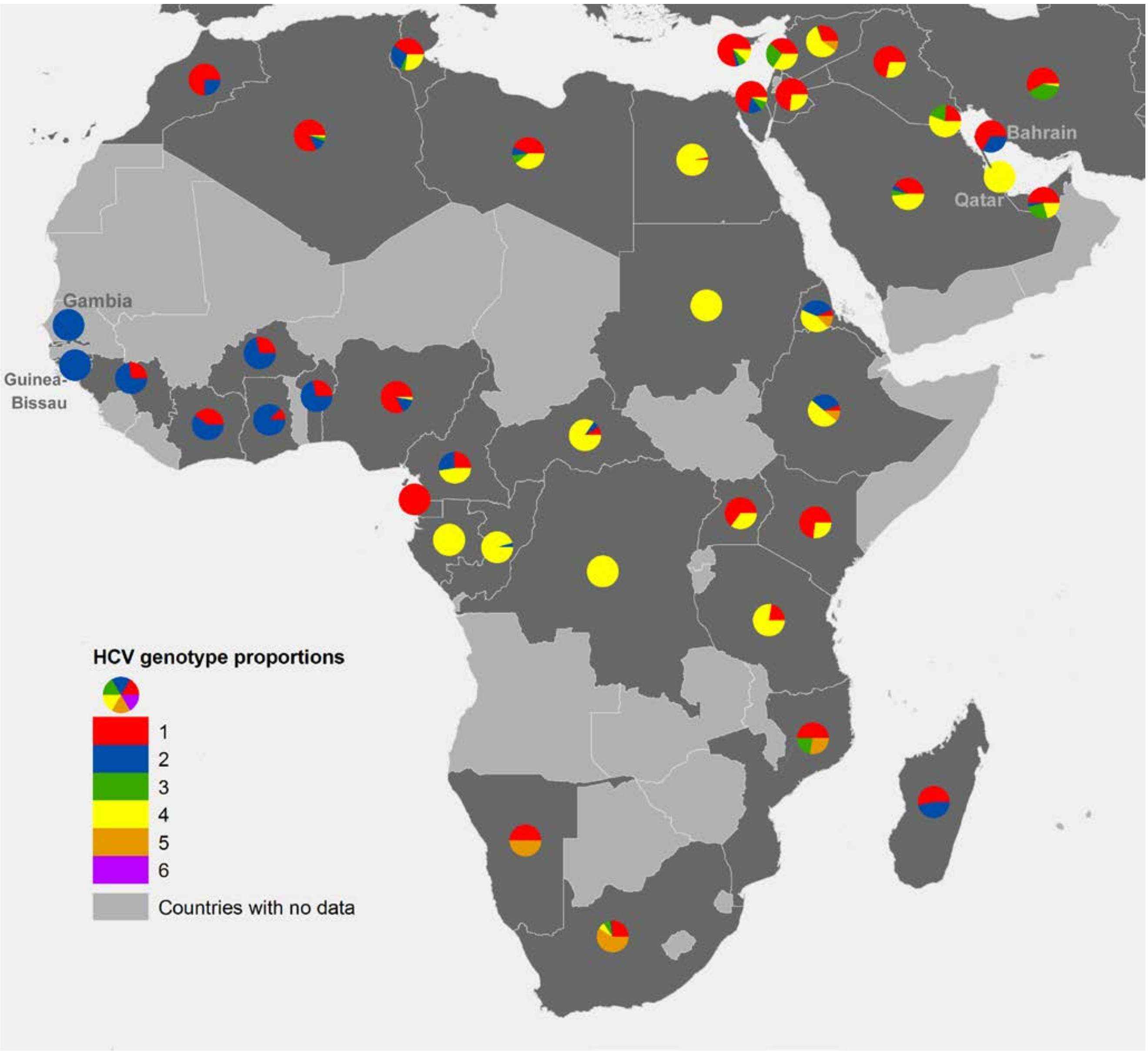

Figure S2: Americas

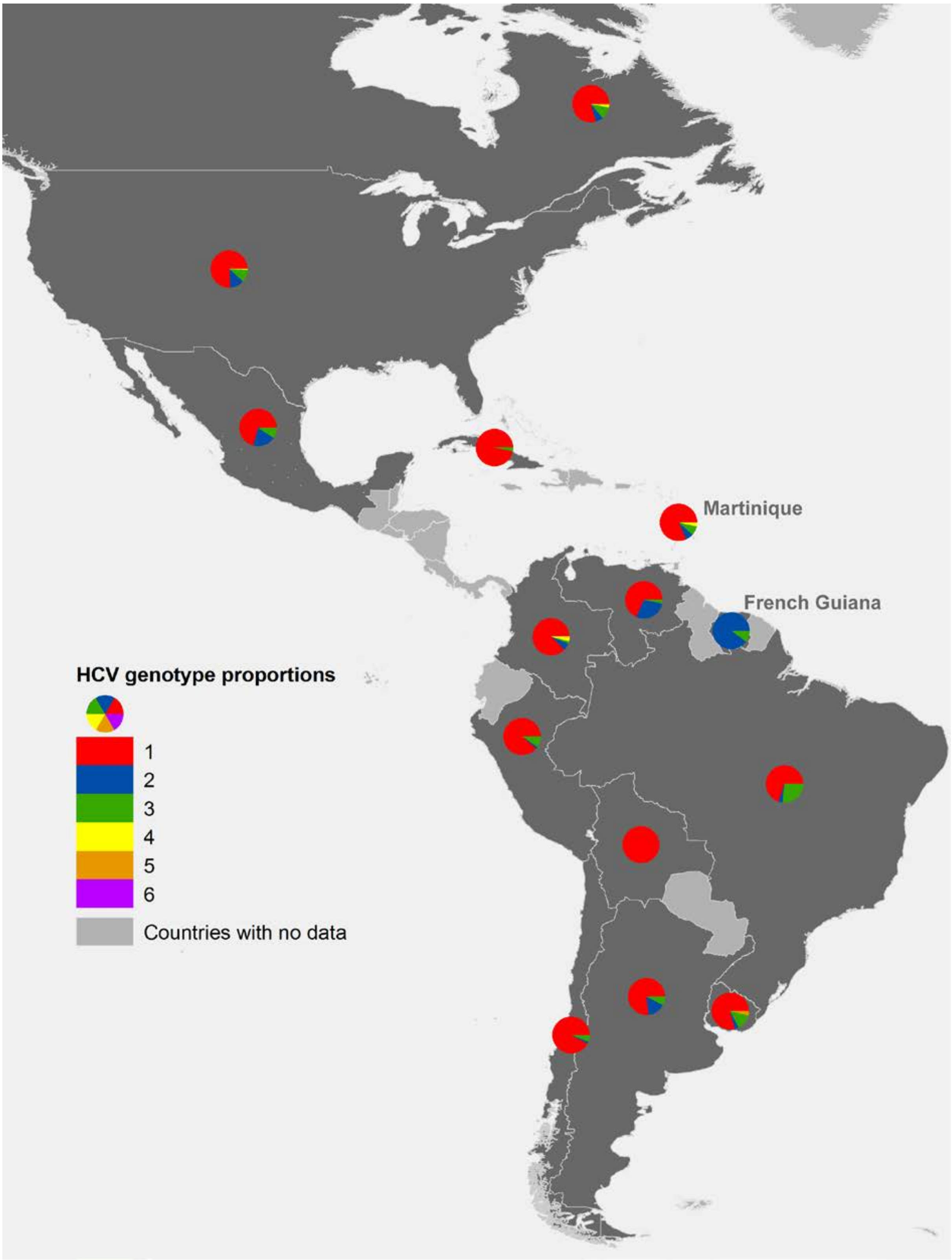

Figure S3: Asia & Middle East

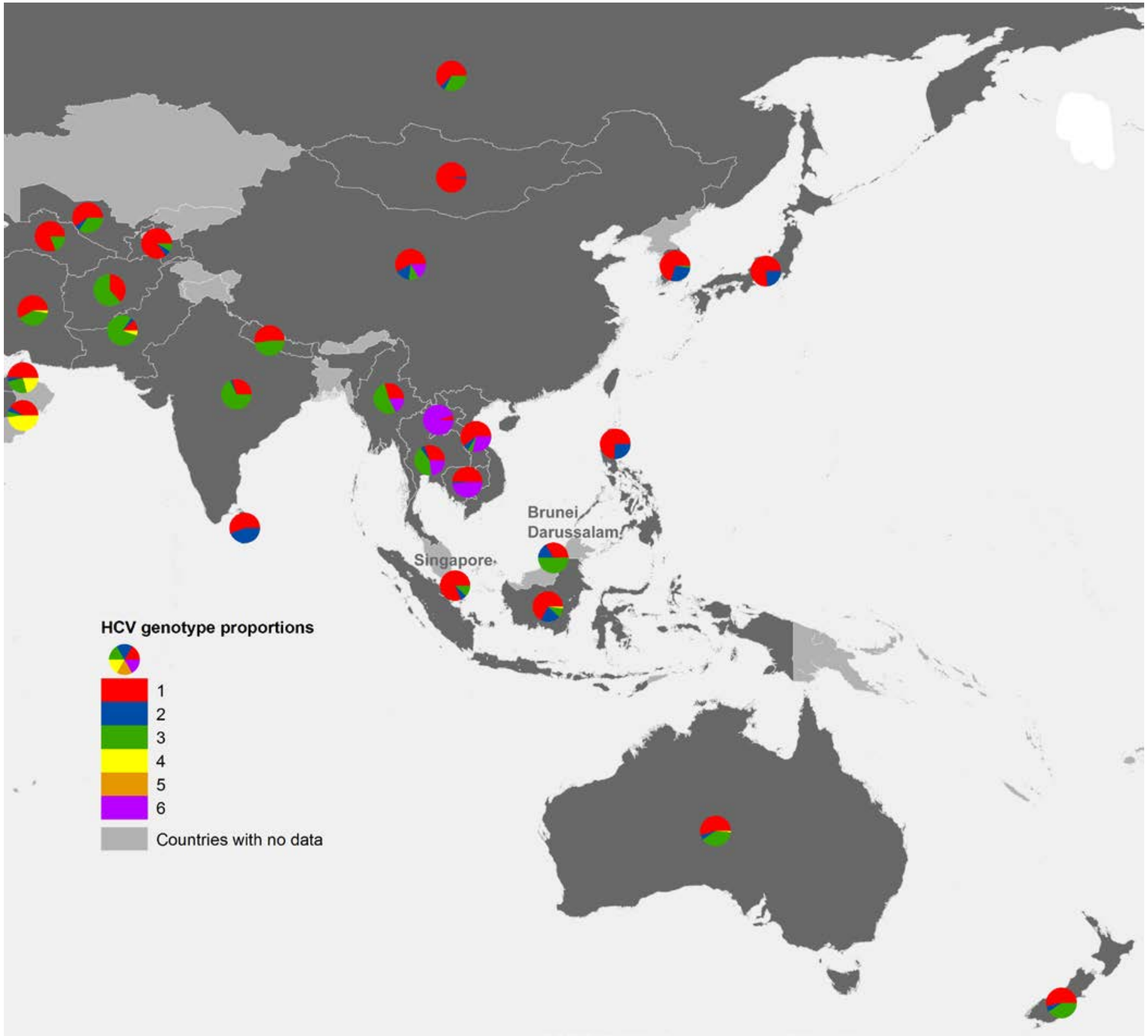

Figure S4: Europe

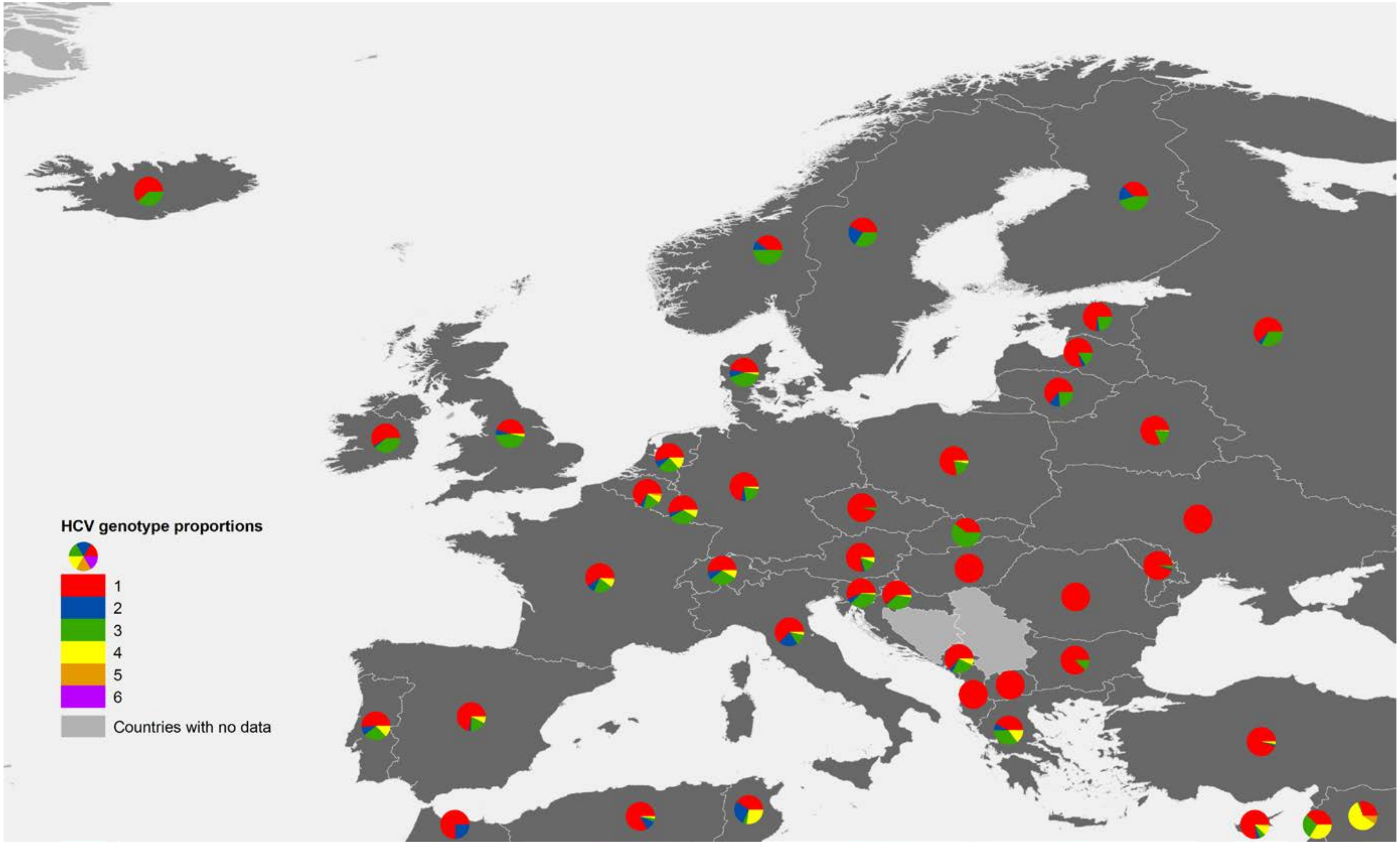

Figure S5

HCV 1a vs. 1b dominance in countries with HCV genotype 1.

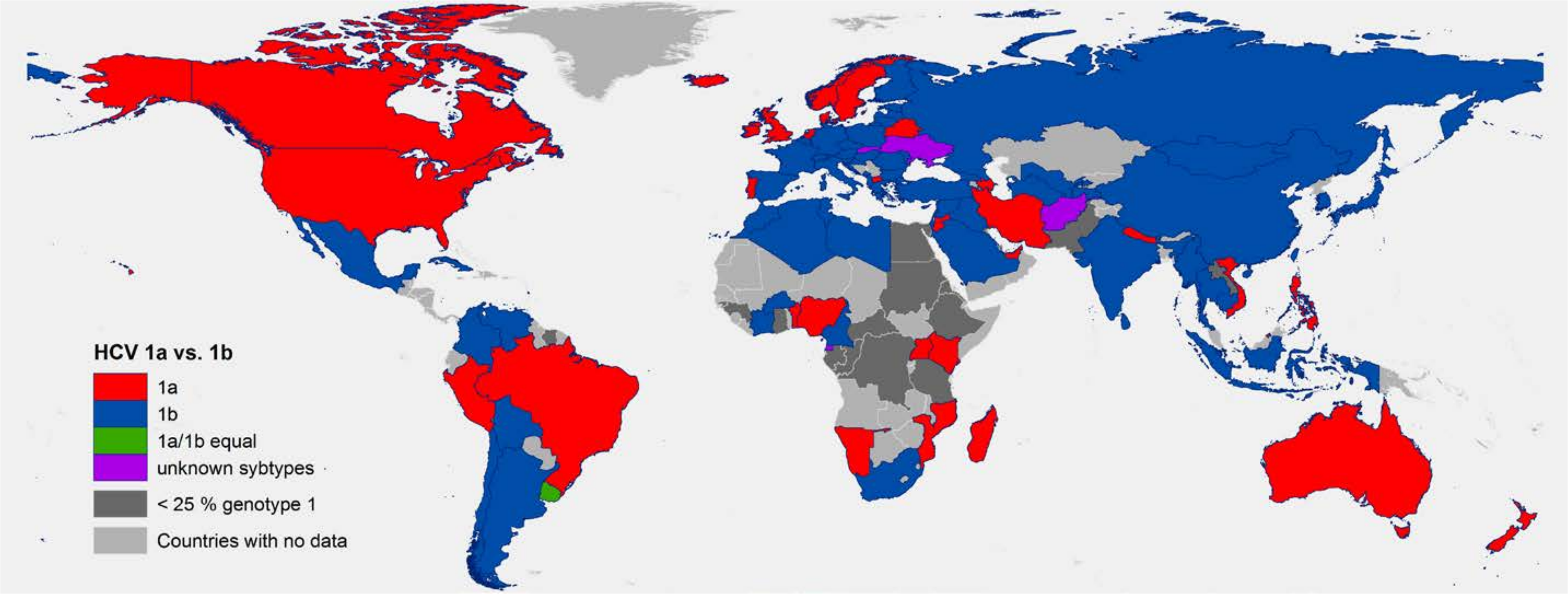

Reference List

A complete list of all published studies used in this analysis.

1 Khattab, M. et al. Changes in Adipocytokines and Insulin Sensitivity during and after Antiviral Therapy for Hepatitis C Genotype 4. Journal of Gastrointestinal Liver Disease 21, 59-65, (2014).

2 Sanders-Buell, E. et al. Hepatitis C genotype distribution and homology among geographically disparate injecting drug users in Afghanistan. J Med Virol 85, 1170-1179, (2013).

3 Rouabhia, S., Sadelaoud, M., Chaabna-Mokrane, K., Toumi, W. & Abenavoli, L. Hepatitis C virus genotypes in north eastern Algeria: A retrospective study. World J Hepatol 5, 393-397, (2013).

4 Iles, J. C. et al. Hepatitis C virus infections in the Democratic Republic of Congo exhibit a cohort effect. Infect Genet Evol 19, 386-394, (2013).

5 Ezzikouri, S., Pineau, P. & Benjelloun, S. Hepatitis C virus infection in the Maghreb region. J Med Virol 85, 1542-1549, (2013).

6 Vicenti, I. et al. Naturally occurring hepatitis C virus (HCV) NS3/4A protease inhibitor resistance-related mutations in HCV genotype 1-infected subjects in Italy. J Antimicrob Chemother 67, 984-987, (2012).

7 Velmishi, V., Dervishi, E., Cullufi, P., Bali, D. & Durro, V. Treatment and follow up of children with chronic hepatitis C in Albania. Virol J 9, 17, (2012).

8 Veldt, B. J. et al. Recipient IL28B polymorphism is an important independent predictor of posttransplant diabetes mellitus in liver transplant patients with chronic hepatitis C. Am J Transplant 12, 737-744, (2012).

9 Shalaby, S. M., Radwan, M. I., Abdelazim, S. & Nafee, A. M. Interleukin-4 polymorphisms and response to combination therapy in Egyptian chronic hepatitis C patients. Cell Immunol 276, 110 - 113, (2012).

10 Romero-Figueroa, S. et al. Risk factors associated with hepatitis C virus infection in an urban population of the State of Mexico. Arch Virol 157, 329-332, (2012).

11 Rodriguez Lay Lde, L. et al. HCV genotype determination in monoinfected and HIV co-infected patients in Cuba. Trans R Soc Trop Med Hyg 106, 711-717, (2012).

12 Rivero-Juárez, A. et al. Association between the IL28B genotype and hepatitis C viral kinetics in the early days of treatment with pegylated interferon plus ribavirin in HIV/HCV co-infected patients with genotype 1 or 4. J Antimicrob Chemother 67, 202-205, (2012).

13 Restivo, L., Zampino, R. & Guerrera…, B. Steatosis is the predictor of relapse in HCV genotype 3-but not 2-infected patients treated with 12 weeks of pegylated interferon-α-2a plus ribavirin and RVR. J Viral Hepat 19, 346-353, (2012).

14 Rao, H.-Y. et al. Outcome of hepatitis C virus infection in Chinese paid plasma donors: A 12–19-year cohort study. Journal of Gastroenterology and Hepatology 27, 526-532, (2012).

15 Raghwani, J. et al. Origin and evolution of the unique hepatitis C virus circulating recombinant form 2k/1b. J Virol 86, 2212-2220, (2012).

16 Oh, D. J., Park, Y. M., Seo, Y. I., Lee, J. S. & Lee, J. Y. Prevalence of Hepatitis C Virus Infections and Distribution of Hepatitis C Virus Genotypes among Korean Blood Donors. Ann Lab Med 32, 210-215, (2012).

17 Nimer, A. & Mouch, A. Vitamin D improves viral response in hepatitis C genotype 2-3 naive patients. World J Gastroenterol 18, 800-805, (2012).

18 Neukam, K. et al. Efficacy of chronic hepatitis C therapy with pegylated interferon and ribavirin in patients on methadone maintenance treatment. Eur J Clin Microbiol Infect Dis 31, 1225-1232, (2012).

19 Miki, D. et al. Serum PAI-1 is a novel predictor for response to pegylated interferon-α-2b plus ribavirin therapy in chronic hepatitis C virus infection. J Viral Hepat 19, e126-e133, (2012).

20 McGilvray, I. et al. Hepatic cell-type specific gene expression better predicts HCV treatment outcome than IL28B genotype. Gastroenterology 142, 1122-1131.e1121, (2012).

21 Maylin, S. et al. Role of hepatic HCV–RNA level on the severity of chronic hepatitis C and response to antiviral therapy. Journal of Clinical Virology 53, 43-47, (2012).

22 Martinez, A. D. et al. Integrated internist - addiction medicine - hepatology model for hepatitis C management for individuals on methadone maintenance. J Viral Hepat 19, 47-54, (2012).

23 Lee, S. S. et al. Randomised clinical trial: the efficacy of treatment, guided by a shorter duration of response, using peginterferon alfa-2a plus ribavirin for hepatitis C virus other than genotypes 2 or 3. Aliment Pharmacol Ther 35, 37-47, (2012).

24 Le Lan, C. et al. A multi-disciplinary approach to treating hepatitis C with interferon and ribavirin in alcohol-dependent patients with ongoing abuse. J Hepatol 56, 334-340, (2012).

25 Lauck, M. et al. Analysis of hepatitis C virus intrahost diversity across the coding region by ultradeep pyrosequencing. J Virol 86, 3952-3960, (2012).

26 Kumada, H. et al. Telaprevir with peginterferon and ribavirin for treatment-naive patients chronically infected with HCV of genotype 1 in Japan. J Hepatol 56, 78-84, (2012).

27 Klade, C., Schuller, E., Boehm, T., von Gabain, A. & Manns, M. Sustained viral load reduction in treatment-naive HCV genotype 1 infected patients after therapeutic peptide vaccination. Vaccine 30, 2943-2950, (2012).

28 Karatapanis, S. et al. Hepatitis C virus genotyping in Greece: unexpected high prevalence of genotype 5a in a Greek island. J Med Virol 84, 223-228, (2012).

29 Karamitros, T. et al. Detection of specific antibodies to HCV-ARF/CORE+1 protein in patients treated with pegylated interferon plus ribavirin. J Viral Hepat 19, 182-188, (2012).

30 Joukar, F., Khalesi, A. K., Jafarshad, R., Rahimabadi, M. S. & Mansour-Ghanaei, F. Distribution of hepatitis C virus genotypes in

haemodialysis patients of Guilan, northern Islamic

Republic of Iran. Eastern Mediterranean Health Journal 18, 236-240, (2012).

31 Jouan, L. et al. Targeted impairment of innate antiviral responses in the liver of chronic hepatitis C patients. J Hepatol 56, 70-77, (2012).

32 Joshita, S. et al. Association of \IL28B\ gene polymorphism with development of hepatocellular carcinoma in Japanese patients with chronic hepatitis C virus infection. Hum Immunol 73, 298 - 300, (2012).

33 Inokuchi, M. et al. Lymphotropic hepatitis C virus has an interferon-resistant phenotype. J Viral Hepat 19, 254-262, (2012).

34 Hartling, H. et al. CD4+ and CD8+ Regulatory T Cells (Tregs) are Elevated and Display an Active Phenotype in Patients with Chronic HCV Mono-Infection and HIV/HCV Co-Infection. Scand J Immunol 76, 294-305, (2012).

35 Halawani, M. & Bakir, T. M. Determination of Hepatitis C Virus Genotypes in Pruritus Patients in Saudi Arabia. Genetic Testing and Molecular Biomarkers 16, 46-49, (2012).

36 Gidding, H. F. et al. Hepatitis C treatment outcomes in Australian clinics. Medical Journal of Australia 196, 633-637, (2012).

37 Gededzha, M. et al. Introduction of new subtypes and variants of hepatitis C virus genotype 4 in South Africa. J Med Virol 84, 601-607, (2012).

38 Fu, Y. et al. HCV 6a prevalence in Guangdong province had the origin from Vietnam and recent dissemination to other regions of China: phylogeographic analyses. PLoS One 7, e28006, (2012).

39 Forbi, J. C. et al. Epidemic history of hepatitis C virus infection in two remote communities in Nigeria, West Africa. Journal of General Virology 93, 1410-1421, (2012).

40 Fattovich, G., Svegliati Baroni, G. & Pasino…, M. Post-load insulin resistance does not predict virological response to treatment of chronic hepatitis C patients without the metabolic syndrome. Digestive and Liver Disease 44, 419-425, (2012).

41 Fabris, C., Falleti, E., Cussigh, A. & Bitetto…, D. The interleukin 28b rs12979860 c/t polymorphism and serum cholesterol as predictors of fibrosis progression in patients with chronic Hepatitis C and persistently …. J Med Virol 84, 747-755, (2012).

42 Eskander, E. F., Abd-Rabou, A. A., Yahya, S. M., Shaker, O. G. & Mohamed, M. S. Does interferon and ribavirin combination therapy ameliorate growth hormone deficiency in \HCV\ genotype-4 infected patients? Clin Biochem 45, 3 - 6, (2012).

43 Dominguez, S. et al. Erythrocyte and plasma ribavirin concentrations in the assessment of early and sustained virological responses to pegylated interferon-alpha 2a and ribavirin in patients coinfected with hepatitis C virus and HIV. Journal of Antimicrobial Chemotherapy 67, 1449-1452, (2012).

44 de Oliveira Crispim, J. C. et al. Upregulation of soluble and membrane-bound human leukocyte antigen G expression is primarily observed in the milder histopathological stages of chronic hepatitis C virus infection. Hum Immunol 73, 258-262, (2012).

45 De Nicola, S., Aghemo, A. & Grazia Rumi…, M. Interleukin 28B polymorphism predicts pegylated interferon plus ribavirin treatment outcome in chronic hepatitis C genotype 4. Hepatology 55, 336-342, (2012).

46 de Castellarnau, M. et al. Deciphering the interleukin 28B variants that better predict response to pegylated interferon-α and ribavirin therapy in HCV/HIV-1 coinfected patients. PLoS One 7, e31016, (2012).

47 Chung, R. T. et al. Extended therapy with pegylated interferon and weight-based ribavirin for HCV-HIV coinfected patients. HIV Clinical Trials 13, 70-82, (2012).

48 Brahim, I. et al. Morocco underwent a drift of circulating hepatitis C virus subtypes in recent decades. Arch Virol 157, 515-520, (2012).

49 Bolcic, F. et al. Analysis of the PKR-eIF2alpha phosphorylation homology domain (PePHD) of hepatitis C virus genotype 1 in HIV-coinfected patients by ultra-deep pyrosequencing and its relationship to responses to pegylated interferon-ribavirin treatment. Arch Virol 157, 703-711, (2012).

50 Bolcic, F., Laufer, N., Trinchero, J., Jones, L. R. & Quarleri, J. A clustering phenomenon among HCV-1a strains among patients coinfected with HIV from Buenos Aires, Argentina. J. Med. Virol. 84, 570-581, (2012).

51 Beinhardt, S. et al. Serum level of IP-10 increases predictive value of IL28B polymorphisms for spontaneous clearance of acute HCV infection. Gastroenterology 142, 78-85.e72, (2012).

52 Anggorowati, N. et al. Clinical and virological characteristics of hepatitis B or C virus co-infection with HIV in Indonesian patients. J. Med. Virol. 84, 857-865, (2012).

53 Aizawa, Y. et al. Genotype rs8099917 near the IL28B gene and amino acid substitution at position 70 in the core region of the hepatitis C virus are determinants of serum apolipoprotein B-100 concentration in chronic hepatitis C. Molecular Cellular Biochemistry 360, 9-14, (2012).

54 Abdelwahab, S. et al. Risk factors for hepatitis C virus infection among Egyptian healthcare workers in a national liver diseases referral centre. Trans R Soc Trop Med Hyg 106, 98 - 103, (2012).

55 Abdel-Moneim, A., Bamaga, M., Shehab, G., Abu-Elsaad, A. & Farahat, F. HCV infection among Saudi population: high prevalence of genotype 4 and increased viral clearance rate. PLoS One 7, e29781, (2012).

56 Zhang, C. et al. HCV subtype characterization among injection drug users: implication for a crucial role of Zhenjiang in HCV transmission in China. PLoS One 6, e16817, (2011).

57 Yokoyama, K. et al. Identification and characterization of a natural inter-genotypic (2b/1b) recombinant hepatitis C virus in Japan. Arch Virol 156, 1591-1601, (2011).

58 Ydreborg, M. et al. Look-back screening for the identification of transfusion-induced hepatitis C virus infection in Sweden. Scand J Infect Dis 43, 522-527, (2011).

59 Wu, C. et al. Alteration of hepatic nuclear receptor-mediated signaling pathways in hepatitis C virus patients with and without a history of alcohol drinking. Hepatology 54, 1966-1974, (2011).

60 Welker, M.-W. et al. CD81 expression for discrimination between sustained virologic response and relapse in patients with chronic hepatitis C. Scand J Gastroenterol 46, 973-980, (2011).

61 Wang, L. et al. Prevalence of abnormal glycometabolism in patients with chronic hepatitis C and related risk factors in China. Chin Med J (Engl) 124, 183-188, (2011).

62 Vieira, D. S. et al. Distribution of hepatitis c virus (hcv) genotypes in patients with chronic infection from Rondônia, Brazil. Virol J 8, 165, (2011).

63 Venturi, C. et al. Long-term outcome of hepatitis C virus infections acquired after pediatric liver transplantation. Liver Transpl 17, 1474-1480, (2011).

64 Venegas, M., Villanueva, R. A., González, K. & Brahm, J. IL28B polymorphisms associated with therapy response in Chilean chronic hepatitis C patients. World J Gastroenterol 17, 3636-3639, (2011).

65 Vejbaesya, S., Nonnoi, Y., Tanwandee, T. & Srinak, D. Killer cell immunoglobulin-like receptors and response to antiviral treatment in Thai patients with chronic hepatitis C virus genotype 3a. J. Med. Virol. 83, 1733-1737, (2011).

66 Valva, P. et al. The role of serum biomarkers in predicting fibrosis progression in pediatric and adult hepatitis C virus chronic infection. PLoS One 6, e23218, (2011).

67 ur Rehman, L. et al. Active hepatitis C infection and HCV genotypes prevalent among the IDUs of Khyber Pakhtunkhwa. Virol J 8, 327, (2011).

68 Tillmann, H. L. et al. Beneficial IL28B genotype associated with lower frequency of hepatic steatosis in patients with chronic hepatitis C. J Hepatol 55, 1195-1200, (2011).

69 Themistoklis, V. et al. Thyroid Dysfunction and Long-term Outcome during and after Interferon-alpha

Therapy in Patients with Chronic Hepatitis C. Ann Acad Singapore 40, 394-400, (2011).

70 Tateyama, M. et al. Alpha-fetoprotein above normal levels as a risk factor for the development of hepatocellular carcinoma in patients infected with hepatitis C virus. J Gastroenterol 46, 92-100, (2011).

71 Sung, P. et al. Frequency of Killer Cell Immunoglobulin-like Receptors (KIRs) in Korean Patients with Chronic HCV Infection. J Korean Med Sci 26, 1483-1488, (2011).

72 Sultana, C. et al. Molecular Epidemiology of Hepatitis C Virus Strains from Romania. Journal of Gastrointestinal Liver Disease 20, 261-266, (2011).

73 Soverini, V., Persico, M., Bugianesi, E. & Forlani…, G. HBV and HCV infection in type 2 diabetes mellitus: a survey in three diabetes units in different Italian areas. Acta Diabetologica 48, 337-343, (2011).

74 Sixtos-Alonso, M. S. et al. IFN-stimulated gene expression is a useful potential molecular marker of response to antiviral treatment with Peg-IFNα 2b and ribavirin in patients with hepatitis C virus genotype 1. Arch Med Res 42, 28-33, (2011).

75 Sistayanarain, A., Kunthalert, D. & Vipsoongnern, Y. A shift in the Hepatitis C virus genotype dominance in blood donor samples from Thailand. Mol Biol Rep 38, 4287-4290, (2011).

76 Sinn, D. et al. Association of a single nucleotide polymorphism near the interleukin-28B gene with response to hepatitis C therapy in Asian patients. Journal of Gastroenterology and Hepatology 26, 1374-1379, (2011).

77 Shi, S., Lu, F., Yan, L. & Zhuang, H. Intrafamilial viral transmission is not the main cause of the high prevalence of hepatic C virus infection in a village, Putian county, China. Journal of Clinical Virology 51, 110-114, (2011).

78 Schwarz, K. B. et al. The combination of ribavirin and peginterferon is superior to peginterferon and placebo for children and adolescents with chronic hepatitis C. Gastroenterology 140, 450-458.e451, (2011).

79 Sawada, L. et al. Distribution of hepatitis C virus genotypes among different exposure categories in the State of Pará, Brazilian Amazon. Rev Soc Bras Med Trop 44, 8-12, (2011).

80 Sarrazin, C. et al. Importance of IL28B gene polymorphisms in hepatitis C virus genotype 2 and 3 infected patients. J Hepatol 54, 415-421, (2011).

81 Sarrazin, C. et al. Improved Responses to Pegylated Interferon Alfa-2b and Ribavirin by Individualizing Treatment for 24–72 Weeks. Gastroenterology 141, 1656-1664, (2011).

82 Santos, B. F. d. O., de Santana, N. O. & Franca, A. V. C. Prevalence, genotypes and factors associated with HCV infection among prisoners in Northeastern Brazil. World J Gastroenterol 17, 3027-3034, (2011).

83 Sanai, F. et al. Updated thresholds for alanine aminotransferase do not exclude significant histological disease in chronic hepatitis C. Liver International 31, 1039-1046, (2011).

84 Saha, K. et al. Recent pattern of Co-infection amongst HIV seropositive individuals in tertiary care hospital, Kolkata. Virol J 8, 116, (2011).

85 Ruiz-Mateos, E. et al. Hepatitis C virus replication in Caucasian HIV controllers. J Viral Hepat 18, e350-357, (2011).

86 Rosenberg, P. & Hagen, K. Serum B12 levels predict response to treatment with interferon and ribavirin in patients with chronic HCV infection. J Viral Hepat 18, 129-134, (2011).

87 Ridruejo, E. et al. Genetic variation in interleukin-28B predicts SVR in hepatitis C genotype 1 Argentine patients treated with PEG IFN and ribavirin. Ann Hepatol 10, 452-457, (2011).

88 Reis, N., Lopes, C. & Teles…, S. Hepatitis C virus infection in patients with tuberculosis in Central Brazil. Int J Tuberc Lung Dis 15, 1137-1402, (2011).

89 Rehan, H. et al. Diversity of genotype and mode of spread of Hepatitis C virus in Northern India. Saudi J Gastroenterol 17, 241-244, (2011).

90 Ré, V. E. et al. Phylodynamics of hepatitis C virus subtype 2c in the province of Córdoba, Argentina. PLoS One 6, e19471, (2011).

91 Rauf, A. et al. Prevalence of hepatitis B and C in internally displaced persons of war against terrorism in Swat, Pakistan. The European Journal of Public Health 21, 638-642, (2011).

92 Rafi, H. et al. Influence of steatosis on progression of fibrosis and virological response in chronic hepatitis C cases. Arab Journal of Gastroenterology 12, 136 - 138, (2011).

93 Pinto, C. S. et al. Hepatitis C virus infection among pregnant women in Central-Western Brazil, 2005-2007. Rev Saude Publica 45, 974-976, (2011).

94 Pavlidis, C. et al. Serum leptin and ghrelin in chronic hepatitis C patients with steatosis. World J Gastroenterol 17, 5097-5104, (2011).

95 Pascu, O., Gheorghe, L., Voiculescu, M., Ceausu, E. & Mateescu, B. How severe is chronic hepatitis with HCV genotype 1b? A study of 1,220 cases on the waiting list for antiviral therapy in Romania. J Gastrointestin Liver Dis 20, 51-55, (2011).

96 Papić, N. et al. Treatment of chronic hepatitis C in Croatian war veterans: experiences from Croatian reference center for viral hepatitis. Croat Med J 52, 35-40, (2011).

97 Obermeier, M. et al. Acute hepatitis C in persons infected with the human immunodeficiency virus (HIV): the "real-life setting" proves the concept. Eur J Med Res 16, 237-242, (2011).

98 Nkontchou, G. et al. HCV genotype 3 is associated with a higher hepatocellular carcinoma incidence in patients with ongoing viral C cirrhosis. J Viral Hepat 18, e516-e522, (2011).

99 Nischalke, H. et al. The PNPLA3 rs738409 148M/M genotype is a risk factor for liver cancer in alcoholic cirrhosis but shows no or weak association in hepatitis C cirrhosis. PLoS One 6, e27087, (2011).

100 Nicot, F. et al. Influence of HCV genotype 1 subtypes on the virus response to PEG interferon alpha-2a plus ribavirin therapy. J. Med. Virol. 83, 437-444, (2011).

101 Nakatani, S. M. et al. Comparative performance evaluation of hepatitis C virus genotyping based on the 5' untranslated region versus partial sequencing of the NS5B region of brazilian patients with chronic hepatitis C. Virol J 8, 459, (2011).

102 Mishra, P. et al. Molecular surveillance of hepatitis and tuberculosis infections in a cohort exposed to methyl isocyanate. International Journal of Occupational Medicine and Environmental Health 24, 94-101, (2011).

103 Mauss, S. et al. Estimating the likelihood of sustained virological response in chronic hepatitis C therapy. J Viral Hepat 18, e81-90, (2011).

104 Martinez, S. M. et al. Assessment of liver fibrosis before and after antiviral therapy by different serum marker panels in patients with chronic hepatitis C. Aliment Pharmacol Ther 33, 138-148, (2011).

105 Marabita, F., Aghemo, A., De Nicola, S. & Rumi…, M. Genetic variation in the interleukin-28B gene is not associated with fibrosis progression in patients with chronic hepatitis C and known date of infection. Hepatology 54, 1127-1134, (2011).

106 Mach, T. H. et al. Efficacy of pegylated interferon alfa-2a or alfa-2b in combination with ribavirin in the treatment of chronic hepatitis caused by hepatitis C virus genotype 1b. Pol Arch Med Wewn 121, 434-439, (2011).

107 Laufer, N. et al. HCV RNA decline in the first 24 h exhibits high negative predictive value of sustained virologic response in HIV/HCV genotype 1 co-infected patients treated with peginterferon and ribavirin. Antiviral Res 90, 92-97, (2011).

108 Laufer, N. et al. No reduction of HCV viral load in HIV patients co-infected with HCV genotype 1 during a 30 days course of nitazoxanide monotherapy. Antiviral Res 92, 497-499, (2011).

109 Larsen, C. et al. Gaining greater insight into HCV emergence in HIV-infected men who have sex with men: the HEPAIG Study. PLoS One 6, e29322, (2011).

110 Langhans, B. et al. Interferon-lambda serum levels in hepatitis C. J Hepatol 54, 859-865, (2011).

111 Kuniholm, M. H. et al. The relation of HLA genotype to hepatitis C viral load and markers of liver fibrosis in HIV-infected and HIV-uninfected women. J Infect Dis 203, 1807-1814, (2011).

112 Kim, J. et al. Acute hepatitis C in Korea: Different modes of infection, high rate of spontaneous recovery, and low rate of seroconversion. J. Med. Virol. 83, 1195-1202, (2011).

113 Kim, A. Y. et al. Spontaneous control of HCV is associated with expression of HLA-B 57 and preservation of targeted epitopes. Gastroenterology 140, 686-696.e681, (2011).

114 Khan, S. et al. Molecular epidemiology of hcv among health care workers of khyber pakhtunkhwa. Virol J 8, 105, (2011).

115 Kawaoka, T. et al. Predictive value of the \IL28B\ polymorphism on the effect of interferon therapy in chronic hepatitis C patients with genotypes 2a and 2b. J Hepatol 54, 408 - 414, (2011).

116 Jurczyk, K. et al. Risk score based PEG Interferon alpha 2b and Ribavirin treatment response estimation model for genotype 1 chronic hepatitis C patients. Adv Med Sci 56, 165-171, (2011).

117 Inamullah et al. Hepatitis C virus genotypes circulating in district Swat of Khyber Pakhtoonkhaw, Pakistan. Virol J 8, 16, (2011).

118 Ijaz, B. et al. Association of laboratory parameters with viral factors in patients with hepatitis C. Virol J 8, 361, (2011).

119 Hübschen, J. et al. High genetic diversity including potential new subtypes of hepatitis C virus genotype 6 in Lao People's Democratic Republic. Clin Microbiol Infect 17, E30-34, (2011).

120 Hiramatsu, N. et al. Pretreatment prediction of anemia progression by pegylated interferon alpha-2b plus ribavirin combination therapy in chronic hepatitis C infection: decision-tree analysis. Journal of Gastroenterology 46, 1111-1119, (2011).

121 Hayat, A. S., Shaikh, N. & Masood, N. Study for frequency and aetiology of lymphadenopathy during

combination therapy for chronic hepatitis C (pegylated interferon alpha plus ribavirin) at a tertiary care hospital in Hyderabad. Journal of Pakistan Medical Association 61, 986-989, (2011).

122 Hayashi, K. et al. Mutations in the core and NS5A region of hepatitis C virus genotype 1b and correlation with response to pegylated-interferon-alpha 2b and ribavirin combination therapy. J Viral Hepat 18, 280-286, (2011).

123 Hashimoto, Y. et al. Prediction of response to peginterferon-alfa-2b plus ribavirin therapy in Japanese patients infected with hepatitis C virus genotype 1b. J. Med. Virol. 83, 981-988, (2011).

124 Grotto, R. M. T., Corvino, S. M., Padovani, J. L., Siqueira, S. M. d. C. & Pardini, M. I. d. M. C. HCV genotype 4 circulating in the city of Franca, São Paulo state, Brazil. Braz J Infect Dis 15, 300, (2011).

125 Gragnani, L., Piluso, A., Giannini, C. & Caini…, P. Genetic determinants in hepatitis C virus–associated mixed cryoglobulinemia: Role of polymorphic variants of BAFF promoter and Fcγ receptors. Arthritis & Rheumatism 63, 1446-1451, (2011).

126 Germer, J. J., Mandrekar, J. N., Bendel, J. L., Mitchell, P. S. & Yao, J. D. C. Hepatitis C virus genotypes in clinical specimens tested at a national reference testing laboratory in the United States. J Clin Microbiol 49, 3040-3043, (2011).

127 García-Álvarez, M. et al. High plasma fractalkine (CX3CL1) levels are associated with severe liver disease in HIV/HCV co-infected patients with HCV genotype 1. Cytokine 54, 244-248, (2011).

128 Furui, Y. et al. Prevalence of amino acid mutation in hepatitis C virus core region among Japanese volunteer blood donors. J Med Virol 83, 1924-1929, (2011).

129 Fransen van de Putte, D. E. et al. Liver stiffness measurements to assess progression of fibrosis in HCV-infected patients with inherited bleeding disorders. Haemophilia 17, e975-980, (2011).

130 Forestier, N. et al. Antiviral activity of danoprevir (ITMN-191/RG7227) in combination with pegylated interferon α-2a and ribavirin in patients with hepatitis C. J Infect Dis 204, 601-608, (2011).

131 Forestier, N. et al. Treatment of chronic hepatitis C patients with the NS3/4A protease inhibitor danoprevir (ITMN-191/RG7227) leads to robust reductions in viral RNA: a phase 1b multiple ascending dose study. J Hepatol 54, 1130-1136, (2011).

132 Florholmen, J. et al. A rapid chemokine response of macrophage inflammatory protein (MIP)-1α, MIP-1β and the regulated on activation, normal T expressed and secreted chemokine is associated with a sustained virological response in the treatment of chronic hepatitis C. Clin Microbiol Infect 17, 204-209, (2011).

133 Feld, J. J. et al. S-adenosyl methionine improves early viral responses and interferon-stimulated gene induction in hepatitis C nonresponders. Gastroenterology 140, 830-839, (2011).

134 Fattovich, G. et al. The homeostasis model assessment of the insulin resistance score is not predictive of a sustained virological response in chronic hepatitis C patients. Liver Int 31, 66-74, (2011).

135 Fattovich, G., Covolo, L. & Bibert…, S. IL28B polymorphisms, IP-10 and viral load predict virological response to therapy in chronic hepatitis C. Aliment Pharmacol Ther 33, 1162-1172, (2011).

136 Falleti, E., Bitetto, D., Fabris, C. & Cussigh…, A. Role of interleukin 28B rs12979860 C/T polymorphism on the histological outcome of chronic hepatitis C: relationship with gender and viral genotype. J Clin Immunol 31, 891-899, (2011).

137 Fabris, C., Vandelli, C. & Toniutto…, P. Apolipoprotein E genotypes modulate fibrosis progression in patients with chronic hepatitis C and persistently normal transaminases. J Gastroenterol Hepatol 26, 328-333, (2011).

138 Eurich, D. et al. Relationship between the interleukin-28b gene polymorphism and the histological severity of hepatitis C virus-induced graft inflammation and the response to antiviral therapy after liver transplantation. Liver Transpl 17, 289-298, (2011).

139 Eurich, D. et al. Transforming growth factor β1 polymorphisms and progression of graft fibrosis after liver transplantation for hepatitis C virus--induced liver disease. Liver Transpl 17, 279-288, (2011).

140 Ergünay, K., Şener, B., Alp, A., Karakaya, J. & Hasçelik, G. Utility of a commercial quantitative hepatitis C virus core antigen assay in a diagnostic laboratory setting. Diagn Microbiol Infect Dis 70, 486-491, (2011).

141 Dzyublyk, I. et al. Controlled release recombinant human interferon-α2b for treating patients with chronic hepatitis C genotype 1: a phase 2a clinical trial. J Viral Hepat 18, 271-279, (2011).

142 Dias, P. T. et al. Temporal changes in HCV genotype distribution in three different high risk populations in San Francisco, California. BMC Infect Dis 11, 208, (2011).

143 Dias, P., Hahn, J., Delwart, E. & Edlin…, B. Temporal changes in HCV genotype distribution in three different high risk populations in San Francisco, California. BMC Infect Dis 11, 208, (2011).

144 Dencs, Á. et al. Phylogenetic investigation of nosocomial transmission of hepatitis C virus in an oncology ward. J. Med. Virol. 83, 428-436, (2011).

145 Deltenre, P. et al. Usefulness of viral kinetics for early prediction of a sustained virological response in HCV-1 non-responders re-treated with pegylated interferon and ribavirin. J Hepatol 55, 989-995, (2011).

146 Coto-Llerena, M. et al. Donor and recipient IL28B polymorphisms in HCV-infected patients undergoing antiviral therapy before and after liver transplantation. Am J Transplant 11, 1051-1057, (2011).

147 Cortes-Mancera, F. et al. Etiology and Viral Genotype in Patients with End-Stage Liver Diseases admitted to a Hepatology Unit in Colombia. Hepat Res Treat 2011, 363205, (2011).

148 Clausen, L. et al. Correlates of spontaneous clearance of hepatitis C virus in a Danish human immunodeficiency virus type 1 cohort. Scand J Infect Dis 43, 798-803, (2011).

149 Ciccozzi, M. et al. Molecular analysis of hepatitis C virus infection in Bulgarian injecting drug users. J. Med. Virol. 83, 1565-1570, (2011).

150 Chevaliez, S. et al. High-Dose Pegylated Interferon-α and Ribavirin in Nonresponder Hepatitis C Patients and Relationship With IL-28B Genotype (SYREN Trial). Gastroenterology 141, 119-127, (2011).

151 Chehadeh, W. et al. Hepatitis C virus infection in a population with high incidence of type 2 diabetes: Impact on diabetes complications. J Infect Public Health 4, 200 - 206, (2011).

152 Chayama, K. et al. Factors predictive of sustained virological response following 72 weeks of combination therapy for genotype 1b hepatitis C. Journal of Gastroenterology 46, 545-555, (2011).

153 Charlton, M. R. et al. Interleukin-28B polymorphisms are associated with histological recurrence and treatment response following liver transplantation in patients with hepatitis C virus infection. Hepatology 53, 317-324, (2011).

154 Chan, D., Lee, S. & Lee, K. The effects of widespread methadone treatment on the molecular epidemiology of hepatitis C virus infection among injection drug users in Hong Kong. J. Med. Virol. 83, 1187-1194, (2011).

155 Calado, R. A. et al. Hepatitis C virus subtypes circulating among intravenous drug users in Lisbon, Portugal. J. Med. Virol. 83, 608-615, (2011).

156 Burguete-García, A. I. et al. Hepatitis C seroprevalence and correlation between viral load and viral genotype among primary care clients in Mexico. Salud Publica Mex 53 Suppl 1, S7-12, (2011).

157 Budkowska, A. et al. Synonymous mutations in the core gene are linked to unusual serological profile in hepatitis C virus infection. PLoS One 6, e15871, (2011).

158 Bonardi, R. et al. Short duration treatment in genotype 1 chronic hepatitis C patients with rapid virologic response to pegylated interferon plus ribavirin. Biomed Pharmacother 65, 303-306, (2011).

159 Bitetto, D., Fattovich, G., Fabris, C., Ceriani, E. & Falleti…, E. Complementary role of vitamin D deficiency and the interleukin-28B rs12979860 C/T polymorphism in predicting antiviral response in chronic hepatitis C. Hepatology 53, 1118-1126, (2011).

160 Bhattacharya, D., Accola, M. A., Ansari, I. H., Striker, R. & Rehrauer, W. M. Naturally occurring genotype 2b/1a hepatitis C virus in the United States. Virol J 8, 458, (2011).

161 Banerjee, A. et al. Transcriptional repression of C4 complement by hepatitis C virus proteins. J Virol 85, 4157-4166, (2011).

162 Aziz, H. et al. Predictors of response to antiviral therapy in patients with chronic hepatitis C from Pakistani population. Chin Med J (Engl) 124, 1333-1337, (2011).

163 Amanzada, A. et al. Ultra-rapid virological response, young age, low γ-GT/ALT-ratio, and absence of steatosis identify a subgroup of HCV Genotype 3 patients who achieve SVR with IFN-α(2a) monotherapy. Dig Dis Sci 56, 3296-3304, (2011).

164 Alvarez, G. C. et al. Danazol improves thrombocytopenia in HCV patients treated with peginterferon and ribavirin. Ann Hepatol 10, 458-468, (2011).

165 Alfaresi, M. Prevalence of hepatitis C virus (HCV) genotypes among positive UAE patients. Mol Biol Rep 38, 2719-2722, (2011).

166 Al-Enzi, S. A., Ismail, W. A., Alsurayei, S. A. & Ismail, A. E. Peginterferon alfa-2b and ribavirin therapy in Kuwaiti patients with chronic hepatitis C virus infection. Eastern Mediterranean Health Journal 17, 669-678, (2011).

167 Akram, M. et al. Effects of host and virus related factors on interferon-α+ribavirin and pegylated-interferon+ribavirin treatment outcomes in chronic Hepatitis C patients. Virol J 8, 234, (2011).

168 Akkarathamrongsin, S. et al. Seroprevalence and genotype of hepatitis C virus among immigrant workers from Cambodia and Myanmar in Thailand. Intervirology 54, 10-16, (2011).

169 Akbar, H. et al. High baseline interleukine-8 level is an independent risk factor for the achievement of sustained virological response in chronic HCV patients. Infect Genet Evol 11, 1301-1305, (2011).

170 Ahmad, W. et al. A brief review on molecular, genetic and imaging techniques for HCV fibrosis evaluation. Virol J 8, 53, (2011).

171 Ackerman, Z., Pappo, O. & Ben-Dov, I. The prognostic value of changes in serum ferritin levels during therapy for hepatitis C virus infection. J Med Virol 83, 1262-1268, (2011).

172 Abu-Mouch, S., Fireman, Z., Jarchovsky, J., Zeina, A. & Assy, N. Vitamin D supplementation improves sustained virologic response in chronic hepatitis C (genotype 1)-naive patients. World J Gastroenterol 17, 5184-5190, (2011).

173 Abreha, T. et al. Genotypes and viral load of hepatitis C virus among persons attending a voluntary counseling and testing center in Ethiopia. J Med Virol 83, 776-782, (2011).

174 Abreha, T. et al. Genotypes and viral load of hepatitis C virus among persons attending a voluntary counseling and testing center in Ethiopia. J Med Virol 83, 776-782, (2011).

175 Abdel-Hady, M. et al. Chronic hepatitis C in children--review of natural history at a National Centre. J Viral Hepat 18, e535-540, (2011).

176 Yue, Q.-h. et al. Anti-HCV reactive volunteer blood donors distribution character and genotypes switch in Xi'an, China. Virol J 7, 186-186, (2010).

177 Vidal-Castiñeira, J. R. et al. Effect of killer immunoglobulin-like receptors in the response to combined treatment in patients with chronic hepatitis C virus infection. J Virol 84, 475-481, (2010).

178 Viazov, S. et al. Hepatitis C virus recombinants are rare even among intravenous drug users. J Med Virol 82, 232-238, (2010).

179 Verbeeck, J. et al. HCV genotype distribution in Flanders and Brussels (Belgium): unravelling the spread of an uncommon HCV genotype 5a cluster. Eur J Clin Microbiol Infect Dis 29, 1427-1434, (2010).

180 van Soest, H. et al. No beneficial effects of amantadine in treatment of chronic hepatitis C patients. Dig Liver Dis 42, 496-502, (2010).

181 Van den Eynde, E. et al. Ability of treatment week 12 viral response to predict long-term outcome in genotype 1 hepatitis C virus/HIV coinfected patients. AIDS 24, 975-982, (2010).

182 Valva, P., De Matteo, E., Galoppo, M. C., Gismondi, M. I. & Preciado, M. V. Apoptosis markers related to pathogenesis of pediatric chronic hepatitis C virus infection: M30 mirrors the severity of steatosis. J. Med. Virol. 82, 949-957, (2010).

183 Vahdat, K. et al. Molecular epidemiology of hepatitis C virus genotypes in Bushehr province, Iran. Eur Rev Med Pharmacol Sci 14, 861-864, (2010).

184 Tanimoto, T. et al. Multiple routes of hepatitis C virus transmission among injection drug users in Hai Phong, Northern Vietnam. J. Med. Virol. 82, 1355-1363, (2010).

185 Suwantarat, N., Tice, A. D., Khawcharoenporn, T. & Chow, D. C. Weight loss, leukopenia and thrombocytopenia associated with sustained virologic response to Hepatitis C treatment. Int J Med Sci 7, 36-42, (2010).

186 Sulbarán, M. Z. et al. Genetic history of hepatitis C virus in Venezuela: high diversity and long time of evolution of HCV genotype 2. PLoS One 5, e14315, (2010).

187 Sosa-Jurado, F. et al. Hepatitis C virus infection in blood donors from the state of Puebla, Mexico. Virol J 7, 18, (2010).

188 Sood, A. et al. How sustained is sustained viral response in patients with hepatitis C virus infection? Indian J Gastroenterol 29, 112-115, (2010).

189 Singhal, A., Jain, A. B., Burke, M. & Black, M. Aggressive use of ribavirin and prolonged course of peginterferon to improve the rate of viral response in liver transplant patients with recurrent hepatitis C viral infection. Exp Clin Transplant 8, 214-219, (2010).

190 Shang, H. et al. High prevalence and genetic diversity of HCV among HIV-1 infected people from various high-risk groups in China. PLoS One 5, e10631, (2010).

191 Seremba, E. et al. Poor performance of hepatitis C antibody tests in hospital patients in Uganda. J. Med. Virol. 82, 1371-1378, (2010).

192 Sedeño-Monge, V. et al. Quantitative analysis of interferon alpha receptor subunit 1 and suppressor of cytokine signaling 1 gene transcription in blood cells of patients with chronic hepatitis C. Virol J 7, 243, (2010).

193 Salim, F. B. et al. Distribution of different hepatitis C virus genotypes in patients with hepatitis C virus infection. World J Gastroenterol 16, 2005-2009, (2010).

194 Rumi, M., Aghemo, A., Prati, G. & D'Ambrosio…, R. Randomized study of peginterferon-α2a plus ribavirin vs peginterferon-α2b plus ribavirin in chronic hepatitis C. Gastroenterology 138, 108-115, (2010).

195 Roomer, R., Hansen, B. E., Janssen, H. L. A. & de Knegt, R. J. Risk factors for infection during treatment with peginterferon alfa and ribavirin for chronic hepatitis C. Hepatology 52, 1225-1231, (2010).

196 Romano, C. M. et al. Social networks shape the transmission dynamics of hepatitis C virus. PLoS One 5, e11170, (2010).

197 Rodrigue-Gervais, I. G. et al. Dendritic cell inhibition is connected to exhaustion of CD8+ T cell polyfunctionality during chronic hepatitis C virus infection. J Immunol 184, 3134-3144, (2010).

198 Ridruejo, E., Adrover, R., Cocozzella, D., Fernández, N. & Reggiardo, M. V. Efficacy, tolerability and safety in the treatment of chronic hepatitis C with combination of PEG-Interferon - Ribavirin in daily practice. Ann Hepatol 9, 46-51, (2010).

199 Reynoso, R., Laufer, N., Bolcic, F. & Quarleri, J. Telomerase activity in peripheral blood mononuclear cells from HIV and HIV-HCV coinfected patients. Virus Res 147, 284-287, (2010).

200 Reiberger, T. et al. HIV-HCV co-infected patients with low CD4+ cell nadirs are at risk for faster fibrosis progression and portal hypertension. J Viral Hepat 17, 400-409, (2010).

201 Piroth, L. et al. Treatment of acute hepatitis C in human immunodeficiency virus-infected patients: the HEPAIG study. Hepatology 52, 1915-1921, (2010).

202 Pineda, J. A. et al. Prevalence and factors associated with significant liver fibrosis assessed by transient elastometry in HIV/hepatitis C virus-coinfected patients. J Viral Hepat 17, 714-719, (2010).

203 Pépin, J. et al. Risk Factors for Hepatitis C Virus Transmission in Colonial Cameroon. Clinical Infectious Diseases 51, 768-776, (2010).

204 Pépin et al. Iatrogenic Transmission of Human T Cell Lymphotropic Virus Type 1 and Hepatitis C Virus through Parenteral Treatment and Chemoprophylaxis of Sleeping Sickness in Colonial Equatorial Africa. Clinical Infectious Diseases 51, 768-776, (2010).

205 Pattullo, V., Ravindran, N. C., Mazzulli, T., Wong, D. K. H. & Heathcote, E. J. Pegylated interferon plus optimized weight-based ribavirin dosing negate the influence of weight and body mass index on early viral kinetics and sustained virological response in chronic hepatitis C. J Viral Hepat 17, 834-838, (2010).

206 Panduro, A. et al. Molecular epidemiology of hepatitis C virus genotypes in west Mexico. Virus Res 151, 19-25, (2010).

207 Panasiuk, A., Parfieniuk, A., Zak, J. & Flisiak, R. Association among Fas expression in leucocytes, serum Fas and Fas-ligand concentrations and hepatic inflammation and fibrosis in chronic hepatitis C. Liver Int 30, 472-478, (2010).

208 Olsen, K. et al. Increased risk of transmission of hepatitis C in open heart surgery compared with vascular and pulmonary surgery. Ann Thorac Surg 90, 1425-1431, (2010).

209 Nilsson, J. & Weiland, O. Effect of control selection on sustained viral response rates in genotype 2/3 HCV mono-infected versus HIV/HCV co-infected patients. Scand J Infect Dis 42, 533-539, (2010).

210 Ngo-Giang-Huong, N. et al. Human immunodeficiency virus-hepatitis C virus co-infection in pregnant women and perinatal transmission to infants in Thailand. Int J Infect Dis 14, e602-607, (2010).

211 Nakamoto, S. et al. Association between mutations in the core region of hepatitis C virus genotype 1 and hepatocellular carcinoma development. J Hepatol 52, 72 - 78, (2010).

212 Nakagawa, M. et al. Mutations in the interferon sensitivity determining region and virological response to combination therapy with pegylated-interferon alpha 2b plus ribavirin in patients with chronic hepatitis C-1b infection. J Gastroenterol 45, 656-665, (2010).

213 Moucari, R. et al. Danoprevir, an HCV NS3/4A protease inhibitor, improves insulin sensitivity in patients with genotype 1 chronic hepatitis C. Gut 59, 1694-1698, (2010).

214 Moretti, F. et al. The hepatitis C virus 5'UTR genomic region remains highly conserved under HAART: a 4- to 8-year longitudinal study from HCV/HIV co-infected patients. AIDS Res Hum Retroviruses 26, 527-532, (2010).

215 Morel, V. et al. Emergence of a genomic variant of the recombinant 2k/1b strain during a mixed Hepatitis C infection: A case report. Journal of Clinical Virology 47, 382-386, (2010).

216 Mora, M. V. A. et al. Molecular characterization, distribution, and dynamics of hepatitis C virus genotypes in blood donors in Colombia. J. Med. Virol. 82, 1889-1898, (2010).

217 Montes-Cano, M. A. et al. Interleukin-28B genetic variants and hepatitis virus infection by different viral genotypes. Hepatology 52, 33-37, (2010).

218 Micheloud, D. et al. CD81 expression in peripheral blood lymphocytes before and after treatment with interferon and ribavirin in HIV/HCV coinfected patients. HIV Medicine 11, 161-169, (2010).

219 Méndez-Navarro, J. et al. A randomized controlled trial of double versus triple therapy with amantadine for genotype 1 chronic hepatitis C in Latino patients. Dig Dis Sci 55, 2629-2635, (2010).

220 Mehta, S. et al. Epidemiology of hepatitis C virus infection & liver disease among injection drug users (IDUs) in Chennai, India. Indian J Med Res 132, 706-714, (2010).

221 Mecenate, F. et al. Short versus standard treatment with pegylated interferon alfa-2A plus ribavirin in patients with hepatitis C virus genotype 2 or 3: the cleo trial. BMC Gastroenterol 10, 21, (2010).

222 Maieron, A. et al. Chronic hepatitis C in Austria, 1992-2006: genotype distribution and demographic factors. Euro Surveill 15, 19492, (2010).

223 Mahfoud, Z., Kassak, K., Kreidieh, K., Shamra, S. & Ramia, S. Distribution of hepatitis C virus genotypes among injecting drug users in Lebanon. Virol J 7, 1-5, (2010).

224 Mahfoud, Z., Kassak, K., Kreidieh, K., Shamra, S. & Ramia, S. Prevalence of antibodies to human Immunodeficiency virus (HIV), hepatitis B and hepatitis C and risk factors in prisoners in Lebanon. J Infect Dev Ctries 4, 144-149, (2010).

225 Loko, M. et al. The French national prospective cohort of patients co-infected with HIV and HCV (ANRS CO13 HEPAVIH): early findings, 2006-2010. BMC Infect Dis 10, 303, (2010).

226 Lee, Y.-M. et al. Molecular epidemiology of HCV genotypes among injection drug users in Taiwan: Full-length sequences of two new subtype 6w strains and a recombinant form_2b6w. J. Med. Virol. 82, 57-68, (2010).

227 Le Pogam, S. et al. RG7128 alone or in combination with pegylated interferon-α2a and ribavirin prevents hepatitis C virus (HCV) Replication and selection of resistant variants in HCV-infected patients. J Infect Dis 202, 1510-1519, (2010).

228 Larsen, C., Bousquet, V., Delarocque-Astagneau, E., Pioche, C. & Roudot-Thoraval, F. Hepatitis C virus genotype 3 and the risk of severe liver disease in a large population of drug users in France. J. Med. Virol. 82, 1647-1654, (2010).

229 Lampe, E., Espirito-Santo, M. P., Martins, R. M. B. & Bello, G. Epidemic history of Hepatitis C virus in Brazil. Infect Genet Evol 10, 886-895, (2010).

230 Lam, K. D. et al. Comparison of surrogate and direct measurement of insulin resistance in chronic hepatitis C virus infection: impact of obesity and ethnicity. Hepatology 52, 38-46, (2010).

231 Krssák, M. et al. Non-invasive assessment of hepatic fat accumulation in chronic hepatitis C by 1H magnetic resonance spectroscopy. Eur J Radiol 74, e60-66, (2010).

232 Keskin, F., Ciftci, S., Turkoglu, S. & Badur, S. Transmission routes of chronic hepatitis C and their relation to HCV genotypes. Turkish Journal of Gastroenterology 21, 396-400, (2010).

233 Kainuma, M. et al. Pegylated interferon α-2b plus ribavirin for older patients with chronic hepatitis C. World J Gastroenterol 16, 4400-4409, (2010).

234 Jobarteh, M. et al. Seroprevalence of hepatitis B and C virus in HIV-1 and HIV-2 infected Gambians. Virol J 7, 1-9, (2010).

235 Jimenez-Mendez, R., Uribe-Salas, F., López-Guillen, P., Cisneros-Garza, L. & Castañeda-Hernandez, G. Distribution of HCV genotypes and HCV RNA viral load in different regions of Mexico. Ann Hepatol 9, 33-39, (2010).

236 Jaroszewicz, J., Rogalska, M., Flisiak, I. & Flisiak, R. Successful antiviral therapy is associated with a decrease of serum prohepcidin in chronic hepatitis C. World J Gastroenterol 16, 1747-1752, (2010).

237 Honda, T. et al. Efficacy of peginterferon-α-2b plus ribavirin in patients aged 65 years and older with chronic hepatitis C. Liver International 30, 527-537, (2010).

238 Halima, S. et al. Serological and molecular expression of Hepatitis B infection in patients with chronic Hepatitis C from Tunisia, North Africa. Virol J 7, 229, (2010).

239 Gonvers, J.-J. et al. Treatment of hepatitis C in HCV mono-infected and in HIV-HCV co-infected patients: an open-labelled comparison study. Swiss Medical Weekly 140, w13055, (2010).

240 Golemba, M. D. et al. High prevalence of hepatitis C virus genotype 1b infection in a small town of Argentina. Phylogenetic and Bayesian coalescent analysis. PLoS One 5, e8751, (2010).

241 Freitas, N. R. et al. Hepatitis C virus infection in Brazilian long-distance truck drivers. Virol J 7, 205, (2010).

242 Fischer, G. E. et al. Hepatitis C virus infections from unsafe injection practices at an endoscopy clinic in Las Vegas, Nevada, 2007-2008. Clin Infect Dis 51, 267-273, (2010).

243 Feld, J. J. et al. Ribavirin improves early responses to peginterferon through improved interferon signaling. Gastroenterology 139, 154-162.e154, (2010).

244 Elasifer, H., Agnnyia, Y., Al-Alagi, B. & Daw, M. Epidemiological manifestations of hepatitis C virus genotypes and its association with potential risk factors among Libyan patients. Virol J 7, 317, (2010).

245 Duarte, C. A. B. et al. A novel hepatitis C virus genotyping method based on liquid microarray. PLoS One 5, (2010).

246 Dore, G. J. et al. Effective Treatment of Injecting Drug Users With Recently Acquired Hepatitis C Virus Infection. Gastroenterology 138, 123 - 135.e122, (2010).

247 Derbala, M., Shebl, F., Rashid, A., Amer, A. & Bener, A. Microalbuminuria in hepatitis C-genotype 4: effect of pegylated interferon and ribavirin. World J Gastroenterol 16, 1226-1231, (2010).

248 de Carvalho-Mello, I. M. V. G. et al. Molecular evidence of horizontal transmission of hepatitis C virus within couples. J Gen Virol 91, 691-696, (2010).

249 Cuenca, F. et al. Predictive baseline criteria of primary therapeutic failure in chronic hepatitis C genotype 1. Rev Esp Enferm Dig 102, 234-238, (2010).

250 Cross, T. J. S., Quaglia, A., Nolan, J., Hughes, S. & Harrison, P. M. Do steatosis and steatohepatitis impact on sustained virological response (SVR) rates in patients receiving pegylated interferon and ribavirin for chronic hepatitis C infection? J. Med. Virol. 82, 958-964, (2010).

251 Costelloe, S. J. et al. Thyroid dysfunction in a UK hepatitis C population treated with interferon-alpha and ribavirin combination therapy. Clin Endocrinol (Oxf) 73, 249-256, (2010).

252 Cooper, C. L., Giordano, C., Mackie, D. & Mills, E. J. Equitable access to HCV care in HIV-HCV co-infection can be achieved despite barriers to health care provision. Ther Clin Risk Manag 6, 207-212, (2010).

253 Cantaloube, J. et al. Analysis of hepatitis C virus strains circulating in Republic of the Congo. J Med Virol 82, 562-567, (2010).

254 Brant, L. J. et al. Planning for the healthcare burden of hepatitis C infection: Hepatitis C genotypes identified in England, 2002-2007. J Clin Virol 48, 115-119, (2010).

255 Bottieau, E., Apers, L., Van-Esbroeck, M., Vandenbruaene, M. & Florence, E. Hepatitis C virus infection in HIV-infected men who have sex with men: sustained rising incidence in Antwerp, Belgium, 2001–2009. Euro Surveill 15, 19673, (2010).

256 Bortoletto, G., Scribano, L. & Realdon…, S. Hyperinsulinaemia reduces the 24-h virological response to PEG-interferon therapy in patients with chronic hepatitis C and insulin resistance. J Viral Hepat 17, 475-480, (2010).

257 Bodlaj, G. et al. Alkaline phosphatase predicts relapse in chronic hepatitis C patients with end-of-treatment response. World J Gastroenterol 16, 2407-2410, (2010).

258 Bittar, C. et al. Genetic diversity of NS5A protein from hepatitis C virus genotype 3a and its relationship to therapy response. BMC Infect Dis 10, 36, (2010).

259 Bertino, G. et al. Epoetin alpha improves the response to antiviral treatment in HCV-related chronic hepatitis. Eur J Clin Pharmacol 66, 1055-1063, (2010).

260 Ascione, A., De Luca, M., Tartaglione, M. & Lampasi…, F. Peginterferon alfa-2a plus ribavirin is more effective than peginterferon alfa-2b plus ribavirin for treating chronic hepatitis C virus infection. Gastroenterology 138, 116-122, (2010).

261 Aparicio, E. et al. IL28B SNP rs8099917 is strongly associated with pegylated interferon-α and ribavirin therapy treatment failure in HCV/HIV-1 coinfected patients. PLoS One 5, e13771, (2010).

262 Amorim, R. M. S. et al. Hepatitis C virus genotypes in hemodialysis patients in the Federal District, Brazil. Rev Inst Med Trop Sao Paulo 52, 57-60, (2010).

263 ALI, A., Ahmed, H. & Idrees, M. Molecular epidemiology of Hepatitis C virus genotypes in Khyber Pakhtoonkhaw of Pakistan. Virol J 7, 203, (2010).

264 Akkarathamrongsin, S. et al. Geographic distribution of hepatitis C virus genotype 6 subtypes in Thailand. J Med Virol 82, 257-262, (2010).

265 Abro, A. H., Al-Dabai, L. & Younis, N. J. Distribution of Hepatitis C virus genotypes in Dubai, United Arab Emirates. Journal of Pakistan Medical Association 60, 987-990, (2010).

266 Zopf, S., Herold, C., Hahn, E. & Ganslmayer, M. Peginterferon alfa-2a relapse rates depend on weight-based ribavirin dosage in HCV-infected patients with genotype 1: results of a retrospective evaluation. Scand J Gastroenterol 44, 486-490, (2009).

267 Zhou, Y. et al. Changes in modes of hepatitis C infection acquisition and genotypes in southwest China. Journal of Clinical Virology 46, 230-233, (2009).

268 Youssef, A. et al. Molecular epidemiological study of hepatitis viruses in Ismailia, Egypt. Intervirology 52, 123-131, (2009).

269 Yoshida, E. M. et al. Re-treatment with peginterferon alfa-2a and ribavirin in patients with chronic hepatitis C who have relapsed or not responded to a first course of pegylated interferon-based therapy. Can J Gastroenterol 23, 180-184, (2009).

270 Weseslindtner, L. et al. Acute infection with a single hepatitis C virus strain in dialysis patients: Analysis of adaptive immune response and viral variability. J Hepatol 50, 693-704, (2009).

271 Wedemeyer, I. et al. Adiponectin inhibits steatotic CD95/Fas up-regulation by hepatocytes: Therapeutic implications for hepatitis C. J Hepatol 50, 140-149, (2009).

272 Wang, Y. et al. A new HCV genotype 6 subtype designated 6v was confirmed with three complete genome sequences. Journal of Clinical Virology 44, 195-199, (2009).

273 Veras, K. N. et al. Chronic hepatitis C virus in the state of Piauí, northeastern Brazil. Braz J Infect Dis 13, 125-129, (2009).

274 Vera-Otarola, J. et al. Hepatitis C virus quasispecies in plasma and peripheral blood mononuclear cells of treatment naïve chronically infected patients. J Viral Hepat 16, 633-643, (2009).

275 van den Berg, C. H. S. B. et al. Never injected, but hepatitis C virus-infected: a study among self-declared never-injecting drug users from the Amsterdam Cohort Studies. J Viral Hepat 16, 568-577, (2009).

276 Urbanus, A. T. et al. Hepatitis C virus infections among HIV-infected men who have sex with men: an expanding epidemic. AIDS 23, F1-7, (2009).

277 TSOCHATZIS, E. et al. Serum HCV RNA levels and HCV genotype do not affect insulin resistance in nondiabetic patients with chronic hepatitis C: a multicentre study. Aliment Pharmacol Ther 30, 947-954, (2009).

278 Sheridan, D. A. et al. Apolipoprotein B-associated cholesterol is a determinant of treatment outcome in patients with chronic hepatitis C virus infection receiving anti-viral agents interferon-alpha and ribavirin. Aliment Pharmacol Ther 29, 1282-1290, (2009).

279 Sharvadze, L., Karchava, M., Bolokadze, N., Gatserelia, L. & Tsertsvadze, T. Safety and efficacy of systematic administration of Filgrastim to prevent neutropenia and infections in patient with hepatitis C. Georgian Med News, 32-35, (2009).

280 Sereno, S., Perinelli, P. & Laghi, V. Changes in the prevalence of hepatitis C virus genotype among Italian injection drug users—Relation to period of injection started. Journal of Clinical Virology 45, 354-357, (2009).

281 Senn, O., Seidenberg, A. & Rosemann, T. Determinants of successful chronic hepatitis C case finding among patients receiving opioid maintenance treatment in a primary care setting. Addiction 104, 2033-2038, (2009).

282 Seme, K. et al. Hepatitis C virus genotypes in 1,504 patients in Slovenia, 1993-2007. J. Med. Virol. 81, 634-639, (2009).

283 Seme, K. et al. Low prevalence of hepatitis B and C infections among HIV-infected individuals in Slovenia: a nation-wide study, 1986-2008. Acta Dermatovenerol Alp Panonica Adriat 18, 153-156, (2009).

284 Selzner, N. et al. Antiviral treatment of recurrent hepatitis C after liver transplantation: predictors of response and long-term outcome. Transplantation 88, 1214-1221, (2009).

285 Ryu, S. et al. Lack of association between genotypes and subtypes of HCV and occurrence of hepatocellular carcinoma in Egypt. J. Med. Virol. 81, 844-847, (2009).

286 Riva, E., Maggi, F., Abbruzzese, F. & Bellomi…, F. Immune complexed (IC) hepatitis C virus (HCV) in chronically and acutely HCV-infected patients. Med Mircobiol Immunol 198, 13-18, (2009).

287 Rauch, A. et al. Divergent adaptation of hepatitis C virus genotypes 1 and 3 to human leukocyte antigen–restricted immune pressure. Hepatology 50, 1017-1029, (2009).

288 Rahmani, M. et al. Clinical outcome of interferon and ribavirin combination treatment in hepatitis C virus infected patients with congenital bleeding disorders in Iran. Haemophilia 15, 1097-1103, (2009).

289 Qureshi, S. et al. Response rates to standard interferon treatment in HCV genotype 3a. J Ayub Med Coll Abbottabad 21, 10-14, (2009).

290 Pizzillo, P., Almasio, P., Ferraro, D. & Craxì…, A. HCV genotypes in Sicily: is there any evidence of a shift? J Med Virol 81, 1040-1046, (2009).

291 Pham, D. A. et al. High Prevalence of Hepatitis C Virus Genotype 6 in Vietnam. Asian Pac J Allegery Immunol 27, 153-160, (2009).

292 Petta, S. et al. Insulin resistance is a major determinant of liver stiffness in nondiabetic patients with HCV genotype 1 chronic hepatitis. Aliment Pharmacol Ther 30, 603-613, (2009).

293 Paintsil, E. et al. Hepatitis C virus infection among drug injectors in St Petersburg, Russia: social and molecular epidemiology of an endemic infection. Addiction 104, 1881-1890, (2009).

294 Osztovits, J. et al. Chronic hepatitis C virus infection associated with autonomic dysfunction. Liver International 29, 1473-1478, (2009).

295 Oliveira, M. L. A. et al. Trends in HCV prevalence, risk factors and distribution of viral genotypes in injecting drug users: findings from two cross-sectional studies. Epidemiol Infect 137, 970-979, (2009).

296 Novais, A. C. M. et al. Prevalence of hepatitis C virus infection and associated factors among male illicit drug users in Cuiabá, Mato Grosso, Brazil. Mem Inst Oswaldo Cruz 104, 892-896, (2009).

297 Nieminen, U., Arkkila, P., Kärkkäinen, P. & Färkkilä, M. Effect of steatosis and inflammation on liver fibrosis in chronic hepatitis C. Liver Int 29, 153-158, (2009).

298 Ndong-Atome, G.-R. et al. Absence of intrafamilial transmission of hepatitis C virus and low risk for sexual transmission in rural central Africa indicate a cohort effect. Journal of Clinical Virology 45, 349 - 353, (2009).

299 Narahari, S., Juwle, A., Basak, S. & Saranath, D. Prevalence and geographic distribution of Hepatitis C Virus genotypes in Indian patient cohort. Infection, Genetics and Evolution 9, 643 - 645, (2009).

300 Mori, N. et al. Randomized trial of high-dose interferon-α-2b combined with ribavirin in patients with chronic hepatitis C: Correlation between amino acid substitutions in the core/NS5A region and virological response to interferon therapy. J. Med. Virol. 81, 640-649, (2009).

301 Moreno, P. et al. Evidence of recombination in Hepatitis C Virus populations infecting a hemophiliac patient. Virol J 6, 203, (2009).

302 Merchante, N. et al. Insulin resistance is not a relevant predictor of sustained virological response to pegylated interferon plus ribavirin in HIV/HCV co-infected patients. J Hepatol 50, 684-692, (2009).

303 Mello, I. M. V. G. C. et al. Conservation of hepatitis C virus nonstructural protein 3 amino acid sequence in viral isolates during liver transplantation. J Viral Hepat 16, 732-737, (2009).

304 McGovern, B. H. et al. Rate of sustained virologic response in relation to baseline hepatitis C virus (HCV) RNA level and rapid virologic clearance in persons with acute HCV infection. J Infect Dis 200, 877-881, (2009).

305 Matos, M. A. D. et al. Epidemiological study of hepatitis A, B and C in the largest Afro-Brazilian isolated community. Trans R Soc Trop Med Hyg 103, 899-905, (2009).

306 Markov, P. V. et al. Phylogeography and molecular epidemiology of hepatitis C virus genotype 2 in Africa. Journal of General Virology 90, 2086-2096, (2009).

307 Mangia, A., Minerva, N., Bacca, D. & Cozzolongo…, R. Determinants of relapse after a short (12 weeks) course of antiviral therapy and re-treatment efficacy of a prolonged course in patients with chronic hepatitis C …. Hepatology 49, 358-363, (2009).

308 Lopes, C. L. R. et al. Prevalence, risk factors and genotypes of hepatitis C virus infection among drug users, Central-Western Brazil. Rev Saude Publica 43 Suppl 1, 43-50, (2009).

309 Lin, L. et al. Quantitation of replication of the HCV genome in human livers with end-stage cirrhosis by strand-specific real-time RT-PCR assays: Methods and clinical relevance. J. Med. Virol. 81, 1569-1575, (2009).

310 Lidman, C. et al. Hepatitis C infection among injection drug users in Stockholm Sweden: prevalence and gender. Scand J Infect Dis 41, 679-684, (2009).

311 Li, C. et al. Complete genomic sequences for hepatitis C virus subtypes 4b, 4c, 4d, 4g, 4k, 4l, 4m, 4n, 4o, 4p, 4q, 4r and 4t. J Gen Virol 90, 1820-1826, (2009).

312 Legrand-Abravanel, F. et al. Influence of the HCV subtype on the virological response to pegylated interferon and ribavirin therapy. J. Med. Virol. 81, 2029-2035, (2009).

313 Lapinski, T. W., Parfieniuk, A., Rogalska-Plonska, M., Czajkowska, J. & Flisiak, R. Prevalence of cryoglobulinaemia in hepatitis C virus- and hepatitis C virus/human immunodeficiency virus-infected individuals: implications for renal function. Liver Int 29, 1158-1161, (2009).

314 Laguno, M. et al. Randomized trial comparing pegylated interferon alpha-2b versus pegylated interferon alpha-2a, both plus ribavirin, to treat chronic hepatitis C in human immunodeficiency virus patients. Hepatology 49, 22-31, (2009).

315 Koskinas, J. et al. Granulocyte colony stimulating factor in HCV genotype-1 patients who develop Peg-IFN-α2b related severe neutropenia: A preliminary report on treatment, safety and efficacy. J. Med. Virol. 81, 848-852, (2009).

316 Kose, S., Gurkan, A., Akman, F., Kelesoglu, M. & Uner, U. Treatment of hepatitis C in hemodialysis patients using pegylated interferon α-2a in Turkey. Journal of Gastroenterology 44, 353-358, (2009).

317 Khan, A. et al. Epidemic spread of hepatitis C virus genotype 3a and relation to high incidence of hepatocellular carcinoma in Pakistan. J Med Virol 81, 1189-1197, (2009).

318 Karchava, M., Sharvadze, L., Gatserelia, L., Badridze, N. & Tsertsvadze, T. Prevailing HCV genotypes and subtypes among hiv infected patients in Georgia. Georgian Med News, 51-55, (2009).

319 Kageyama, S. et al. Tracking the entry routes of hepatitis C virus as a surrogate of HIV in an HIV-low prevalence country, the Philippines. J Med Virol 81, 1157-1162, (2009).

320 Kaabia, N. et al. Association of hepatitis C virus infection and diabetes in central Tunisia. World J Gastroenterol 15, 2778-2781, (2009).

321 Jutavijittum, P. et al. Genotypic distribution of hepatitis C virus in voluntary blood donors of northern Thailand. Southeast Asian J Trop Med Public Health 40, 471-479, (2009).

322 Jia, L. et al. HCV Antibody Response and Genotype Distribution in Different Areas and Races of China. Int J Biol Sci 5, 421-427, (2009).

323 Jardim, A. C. G. et al. Quasispecies of hepatitis C virus genotype 1 and treatment outcome with peginterferon and ribavirin. Infect Genet Evol 9, 689-698, (2009).

324 Jack, K., Willott, S., Manners, J., Varnam, M. A. & Thomson, B. J. Clinical trial: a primary-care-based model for the delivery of anti-viral treatment to injecting drug users infected with hepatitis C. Aliment Pharmacol Ther 29, 38-45, (2009).

325 Idrees, M. & Riazuddin, S. A study of best positive predictors for sustained virologic response to interferon alpha plus ribavirin therapy in naive chronic hepatitis C patients. BMC Gastroenterol 9, 5, (2009).

326 Idrees, M. et al. Hepatitis C virus genotype 3a infection and hepatocellular carcinoma: Pakistan experience. World J Gastroenterol 15, 5080-5085, (2009).

327 Hansen, N. et al. Predictors of antiviral treatment initiation in hepatitis C virus-infected patients: a Danish cohort study. J Viral Hepat 16, 659-665, (2009).

328 Grigorescu, M. HCV Genotype 1 is Almost Exclusively Present in Romanian Patients with Chronic Hepatitis C. Journal of Gastrointestinal Liver Disease 18, 45-50, (2009).

329 Grasso, A., Malfatti, F., Leo, P., Martines, H. & Fabris…, P. Insulin resistance predicts rapid virological response in non-diabetic, non-cirrhotic genotype 1 HCV patients treated with peginterferon alpha-2b plus ribavirin. J Hepatol 51, 984-990, (2009).

330 Giugliano, S. et al. Degree of cross-genotype reactivity of hepatitis C virus–specific CD8+ T cells directed against NS3. Hepatology 50, 707-716, (2009).

331 Gismondi, M. I. et al. Evolution of hepatitis C virus hypervariable region 1 in immunocompetent children born to HCV-infected mothers. J Viral Hepat 16, 332-339, (2009).

332 Giannini, E. G., Basso, M., Savarino, V. & Picciotto, A. Predictive value of on-treatment response during full-dose antiviral therapy of patients with hepatitis C virus cirrhosis and portal hypertension. J Intern Med 266, 537-546, (2009).

333 Gheorghe, L. et al. High sustained virological response rate to combination therapy in genotype 1 patients with histologically mild hepatitis C. J Gastrointestin Liver Dis 18, 51-56, (2009).

334 George, S. L. et al. Clinical, virologic, histologic, and biochemical outcomes after successful HCV therapy: a 5-year follow-up of 150 patients. Hepatology 49, 729-738, (2009).

335 Gazdik, F. et al. High virologic sustained response for former young intravenous drug users with chronic hepatitis C treated by pegylated interferon-alpha plus ribavirin. Bratisl Lek Listy 110, 77-84, (2009).

336 Fierbinteanu-Braticevici, C. et al. Role of oxidative stress in the pathogenesis of chronic hepatitis C (CHC). Rom J Morphol Embryol 50, 407-412, (2009).

337 Elkady, A. et al. Genetic variability of hepatitis C virus in South Egypt and its possible clinical implication. J. Med. Virol. 81, 1015-1023, (2009).

338 Elefsiniotis, I., Vezali, E., Mihas, C. & Saroglou, G. Predictive value of complete and partial early virological response on sustained virological response rates of genotype-4 chronic hepatitis C patients treated with PEG-interferon plus ribavirin. Intervirology 52, 247-251, (2009).

339 Elefsiniotis, I. et al. Differential viral kinetics in treated genotype 4 chronic hepatitis C patients according to ethnicity. J Viral Hepat 16, 738-742, (2009).

340 Durante-Mangoni, E. & Zampino…, R. Correlates and prognostic value of the first-phase hepatitis C virus RNA kinetics during treatment. Clin Infect Dis 49, 498-506, (2009).

341 Dryer, P. D. et al. Screening for hepatitis C virus non-nucleotide resistance mutations in treatment-naive women. J Antimicrob Chemother 64, 945-948, (2009).

342 Di Lello, F. A., Piñeiro Y Leone, F. G., Muñoz, G. & Campos, R. H. Diversity of hepatitis B and C viruses in Chile. J. Med. Virol. 81, 1887-1894, (2009).

343 Demetriou, V., van de Vijver, D., null & Kostrikis, L. Molecular epidemiology of hepatitis C infection in Cyprus: evidence of polyphyletic infection. J Med Virol 81, 238-248, (2009).

344 de Bruijne, J. et al. Emergence of hepatitis C virus genotype 4: phylogenetic analysis reveals three distinct epidemiological profiles. J Clin Microbiol 47, 3832-3838, (2009).

345 Dahlan, Y., Ather, H., Al-ahmadi, M., Batwa, F. & Al-hamoudi, W. Sustained virological response in a predominantly hepatitis C virus genotype 4 infected population. World J Gastroenterol 15, 4429-4433, (2009).

346 Cozzolongo, R., Osella, A., Elba, S. & Petruzzi…, J. Epidemiology of HCV infection in the general population: a survey in a southern Italian town. Am J Gastroenterol 104, 2740-2746, (2009).

347 Cooper, C. et al. Evaluation of VCH-759 monotherapy in hepatitis C infection. J Hepatol 51, 39-46, (2009).

348 Chuang, J. Y. et al. IL-10 promoter gene polymorphisms and sustained response to combination therapy in Taiwanese chronic hepatitis C patients. Digestive and Liver Disease 41, 424-430, (2009).

349 Chew, K. W., Allen, S. A., Taylor, L. E., Rich, J. D. & Feller, E. Treatment outcomes with pegylated interferon and ribavirin for male prisoners with chronic hepatitis C. J Clin Gastroenterol 43, 686-691, (2009).

350 Cavlek, T., Margan, I., Lepej, S., Kolaric, B. & Vince, A. Seroprevalence, risk factors, and hepatitis C virus genotypes in groups with high-risk sexual behavior in Croatia. J. Med. Virol. 81, 1348-1353, (2009).

351 Cavalheiro, N. d. P. et al. Hepatitis C: sexual or intrafamilial transmission? Epidemiological and phylogenetic analysis of hepatitis C virus in 24 infected couples. Rev Soc Bras Med Trop 42, 239-244, (2009).

352 Carvalho, F. H. P. d., Coêlho, M. R. C. D., Vilella, T. d. A. S., Silva, J. L. A. & Melo, H. R. d. L. [HIV/HCV coinfection at an university hospital in Recife, Brazil]. Rev Saude Publica 43, 133-139, (2009).

353 Calderón, G. M. et al. Prevalence and risk factors of hepatitis C virus, hepatitis B virus, and human immunodeficiency virus in multiply transfused recipients in Mexico. Transfusion 49, 2200-2207, (2009).

354 Cacopardo, B., Nunnari, G., Benanti, F. & Cappellani…, A. Leukocyte interferon alpha early retreatment for Child A HCV genotype 1b-infected cirrhotics intolerant to pegylated interferons. Infection 37, 210-215, (2009).

355 BREILH, D. et al. Impact of ribavirin plasma level on sustained virological response in patients treated with pegylated interferon and ribavirin for chronic hepatitis C. Aliment Pharmacol Ther 30, 487-494, (2009).

356 Bessa, M. et al. Limited evidence of HCV transmission in stable heterosexual couples from Bahia, Brazil. Braz J Infect Dis 13, 262-265, (2009).

357 Barbosa, A. d. J. et al. Analysis of GB virus C infection among HIV-HCV coinfected patients. Rev Soc Bras Med Trop 42, 591-593, (2009).

358 Assarehzadegan, M., Shakerinejad, G., Noroozkohnejad, R., Amini, A. & Rahim Rezaee, S. Prevalence of hepatitis C and B infection and HC V genotypes among hemodialysis patients in Khuzestan province, southwest Iran. Saudi J Kidney Dis Transpl 20, 681-684, (2009).

359 Askar, E. et al. TLR3 gene polymorphisms and liver disease manifestations in chronic hepatitis C. J Med Virol 81, 1204-1211, (2009).

360 Araújo, F. M. G., Machado-Lima, A., Durham, A. M., Teixeira, R. & Oliveira, G. Sequence and structural analysis of the 5' noncoding region of hepatitis C virus in patients with chronic infection. J. Med. Virol. 81, 1212-1219, (2009).

361 ANTAKI, N. et al. The unexpected discovery of a focus of hepatitis C virus genotype 5 in a Syrian province. Epidemiology & Infection 137, 79-84 M73 - 10.1017/S095026880800054X, (2009).

362 Andriulli, A. et al. Early discontinuation of ribavirin in HCV-2 and HCV-3 patients responding to Peg-interferon alpha-2a and ribavirin. J Viral Hepat 16, 28-35, (2009).

363 Alzahrani, A. et al. Molecular detection of hepatitis B, hepatitis C, and torque teno viruses in drug users in Saudi Arabia. J. Med. Virol. 81, 1343-1347, (2009).

364 Alavian, S.-M. et al. Distribution of hepatitis C virus genotype in Iranian multiply transfused patients with thalassemia. Transfusion 49, 2195-2199, (2009).

365 Akhavan, S. et al. Natural Variability of NS3 Protease in Patients Infected with Genotype 4 Hepatitis C Virus (HCV): Implications for Antiviral Treatment Using Specifically Targeted Antiviral Therapy for HCV. Journal of Infectious Diseases 200, 524-527, (2009).

366 Aghemo, A., Rumi, M., Monico, S. & Prati…, G. The pattern of pegylated interferon-alpha2b and ribavirin treatment failure in cirrhotic patients depends on hepatitis C virus genotype. Antivir Ther 18, 577-584, (2009).

367 Xia, X. et al. The unique HCV genotype distribution and the discovery of a novel subtype 6u among IDUs co-infected with HIV-1 in Yunnan, China. J. Med. Virol. 80, 1142-1152, (2008).

368 Weiland, O. et al. Lower-than-standard dose peg-IFN alfa-2a for chronic hepatitis C caused by genotype 2 and 3 is sufficient when given in combination with weight-based ribavirin. J Viral Hepat 15, 641-645, (2008).

369 von Wagner, M. et al. Placebo-controlled trial of 400 mg amantadine combined with peginterferon alfa-2a and ribavirin for 48 weeks in chronic hepatitis C virus-1 infection. Hepatology 48, 1404-1411, (2008).

370 Vidali, M. et al. Interplay between oxidative stress and hepatic steatosis in the progression of chronic hepatitis C. J Hepatol 48, 399-406, (2008).

371 Verma, V., Chakravarti, A. & Kar, P. Genotypic characterization of hepatitis C virus and its significance in patients with chronic liver disease from Northern India. Diagn Microbiol Infect Dis 61, 408-414, (2008).

372 Tural, C. et al. Differences in virological response to pegylated interferon and ribavirin between hepatitis C virus (HCV)-monoinfected and HCV-HIV-coinfected patients. Antivir Ther 13, 1047-1055, (2008).

373 Tsertsvadze, T., Sharvadze, L., Dzigua, L., Dolmazashvili, E. & Nelson, K. Acute/recent HCV infection. Clinical course, viral replication kunetic and disease outcome. Georgian Med News, 43-49, (2008).

374 Tan, Y. et al. Molecular epidemiology of HCV monoinfection and HIV/HCV coinfection in injection drug users in Liuzhou, Southern China. PLoS One 3, e3608, (2008).

375 Syed, E. et al. Pegylated interferon and ribavirin combination therapy for chronic hepatitis C virus infection in patients with Child-Pugh Class A liver cirrhosis. Scand J Gastroenterol 43, 1378-1386, (2008).

376 Somi, M. H., Keivani, H., Ardalan, M. R., Farhang, S. & Pouri, A. A. Hepatitis C virus Genotypes in Patients with End-Stage Renal Disease in East Azerbaijan, Iran. Saudi J Kidney Dis Transpl 19, 461-465, (2008).

377 Sobesky, R. et al. Pathological evolution of hepatitis C virus-"Healthy carriers". World J Gastroenterol 14, 3861-3865, (2008).

378 Slavenburg, S. et al. Prevalence of hepatitis C in the general population in the Netherlands. Neth J Med 66, 13-17, (2008).

379 Simanis, R. et al. Natural clearance of hepatitis C virus in hemophilia patients. Medicina Kaunas 44, 15-21, (2008).

380 Sikuler, E. et al. Sustained Virologic Response to Treatment in 100% of Patients Recently Infected, Nosocomially, With HCV Genotype 2. J Clin Gastroenterol 42, (2008).

381 Sharvadze, L., Nelson, K., Imnadze, P., Karchava, M. & Tsertsvadze, T. Prevalence of HCV and genotypes distribution in general population of Georgia. Georgian Med News, 71-77, (2008).

382 Senevirathna, D. et al. GENOTYPES OF HEPATITIS C VIRUS (HCV) IN LIVER

DISEASE PATIENTS IN SRI LANKA. Southeast Asian J Trop Med Public Health 39, 1054-1056, (2008).

383 Scherzer, T. et al. Efficacy and safety of antiviral therapy in patients with Crohn's disease and chronic hepatitis C. Aliment Pharmacol Ther 28, 742-748, (2008).

384 Romero-Gómez, M. et al. Effect of sustained virological response to treatment on the incidence of abnormal glucose values in chronic hepatitis C. J Hepatol 48, 721-727, (2008).

385 Roman, F. et al. Hepatitis C virus genotypes distribution and transmission risk factors in Luxembourg from 1991 to 2006. World J Gastroenterol 14, 1237-1243, (2008).

386 Roffi, L., Colloredo, G., Pioltelli, P., Bellati, G. & Pozzpi…, M. Pegylated interferon-alpha2b plus ribavirin: an efficacious and well-tolerated treatment regimen for patients with hepatitis C virus related histologically proven …. Antivir Ther 13, 663-673, (2008).

387 Rivas-Estilla, A. M. et al. Genotyping of hepatitis C virus (HCV) in infected patients from Northeast Mexico. Ann Hepatol 7, 144-147, (2008).

388 Reiberger, T. et al. Efficacy of interferon in immunocompromised HCV patients after liver transplantation or with HIV co-infection. Eur J Clin Invest 38, 421-429, (2008).

389 Reiberger, T. et al. IP-10 correlates with hepatitis C viral load, hepatic inflammation and fibrosis and predicts hepatitis C virus relapse or non-response in HIV-HCV coinfection. Antivir Ther 13, 969-976, (2008).

390 Ré, V. et al. Hepatitis C and HIV coinfection in central region of Argentina: prevalence, genotype characterization and risk factors. Enferm Infecc Microbiol Clin 26, 423-425, (2008).

391 Ramarokoto, C. et al. Seroprevalence of hepatitis C and associated risk factors in urban areas of Antananarivo, Madagascar. BMC Infect Dis 8, 25, (2008).

392 Prabdial-Sing, N., Puren, A., Mahlangu, J., Barrow, P. & Bowyer, S. Hepatitis C virus genotypes in two different patient cohorts in Johannesburg, South Africa. Arch Virol 153, 2049-2058, (2008).

393 Poo, J. L. et al. Efficacy of triple therapy with thymalfasin, peginterferon alpha-2a, and ribavirin for the treatment of hispanic chronic HCV nonresponders. Ann Hepatol 7, 369-375, (2008).

394 Pham, T. N. Q. et al. Hepatitis C virus replicates in the same immune cell subsets in chronic hepatitis C and occult infection. Gastroenterology 134, 812-822, (2008).

395 Petta, S., Cammà, C., Di Marco, V. & Alessi…, N. Insulin resistance and diabetes increase fibrosis in the liver of patients with genotype 1 HCV infection. Am J Gastroenterol 103, 1136-1144, (2008).

396 Pessôa, M. G. et al. Post-transplant recurrent hepatitis C: immunohistochemical detection of hepatitis C virus core antigen and possible pathogenic implications. Liver Int 28, 807-813, (2008).

397 Persico, M., Capasso, M., Russo, R., Persico, E. & Croce…, L. Elevated expression and polymorphisms of SOCS3 influence patient response to antiviral therapy in chronic hepatitis C. Gut 57, 507-515, (2008).

398 Olinger, C., Lazouskaya, N., Eremin, V. & Muller, C. Multiple genotypes and subtypes of hepatitis B and C viruses in Belarus: similarities with Russia and western European influences. Clin Microbiol Infect 14, 575-581, (2008).

399 Noppornpanth, S. et al. Complete genome analysis of hepatitis C virus subtypes 6t and 6u. J Gen Virol 89, 1276-1281, (2008).

400 Nguyen, M. H. et al. Higher rate of sustained virologic response in chronic hepatitis C genotype 6 treated with 48 weeks versus 24 weeks of peginterferon plus ribavirin. Am J Gastroenterol 103, 1131-1135, (2008).

401 Ndong-Atome, G.-R. et al. Hepatitis C virus prevalence and genetic diversity among pregnant women in Gabon, central Africa. BMC Infect Dis 8, 1-7, (2008).

402 Nattermann, J. et al. The transforming growth factor-β high-producer genotype is associated with response to hepatitis C virus-specific therapy in HIV-positive patients with acute hepatitis C. AIDS 22, (2008).

403 Musaya, T. et al. Prevalence of hePatitis c virus and its genotyPes among a cohort of

drug users in Kenya. East Afr Med J, 1-8, (2008).

404 Muhanna, N. et al. Activation of hepatic stellate cells after phagocytosis of lymphocytes: A novel pathway of fibrogenesis. Hepatology 48, 963-977, (2008).

405 Moucari, R. et al. Insulin Resistance in Chronic Hepatitis C: Association With Genotypes 1 and 4, Serum HCV RNA Level, and Liver Fibrosis. Gastroenterology 134, 416-423, (2008).

406 Moreau, I., Levis, J., Crosbie, O., Kenny-Walsh, E. & Fanning, L. Correlation between pre-treatment quasispecies complexity and treatment outcome in chronic HCV genotype 3a. Virol J 5, 78, (2008).

407 Mira, J. A. et al. Efficacy of pegylated interferon plus ribavirin treatment in HIV/hepatitis C virus co-infected patients receiving abacavir plus lamivudine or tenofovir plus either lamivudine or emtricitabine as nucleoside analogue backbone. J Antimicrob Chemother 62, 1365-1373, (2008).

408 Micalessi, M. et al. Distribution of hepatitis C virus genotypes among injecting drug users in contact with treatment centers in Belgium, 2004-2005. J Med Virol 80, 640-645, (2008).

409 Mehta, S. H. et al. Limited uptake of hepatitis C treatment among injection drug users. J Community Health 33, 126-133, (2008).

410 McPherson, S. et al. Investigation of the role of SREBP-1c in the pathogenesis of HCV-related steatosis. J Hepatol 49, 1046 - 1054, (2008).

411 Maticic, M., Poljak, M., Lunder, T., Rener-Sitar, K. & Stojanovic, L. Lichen planus and other cutaneous manifestations in chronic hepatitis C: pre- and post-interferon-based treatment prevalence vary in a cohort of patients from low hepatitis C virus endemic area. J Eur Acad Dermatol Venereol 22, 779-788, (2008).

412 Matheï, C. et al. The epidemic history of hepatitis C among injecting drug users in Flanders, Belgium. J Viral Hepat 15, 399-408, (2008).

413 Mangia, A. et al. Individualized treatment duration for hepatitis C genotype 1 patients: A randomized controlled trial. Hepatology 47, 43-50, (2008).

414 Makhoul, N. J. et al. Distribution of hepatitis C virus (HCV) genotypes among HCV infection risk groups in Lebanon. Journal of Clinical Virology 41, 166-167, (2008).

415 Lu, L. et al. Complete genomes of three subtype 6t isolates and analysis of many novel hepatitis C virus variants within genotype 6. J Gen Virol 89, 444-452, (2008).

416 Lopez-Cortes, L. F. et al. Role of pegylated interferon-alpha-2a and ribavirin concentrations in sustained viral response in HCV/HIV-coinfected patients. Clin Pharmacol Ther 84, 573-580, (2008).

417 Loguercio, C., Federico, A. & Masarone…, M. The impact of diet on liver fibrosis and on response to interferon therapy in patients with HCV-related chronic hepatitis. Am J Gastroenterol 103, 3159-3166, (2008).

418 Liu, P. et al. Molecular Epidemiology of Human Immunodeficiency Virus Type 1 and Hepatitis C Virus in Former Blood Donors in Central China. AIDS Res Hum Retroviruses 24, 1-6, (2008).

419 Liu, J. et al. Extremely high prevalence and genetic diversity of hepatitis C virus infection among HIV-infected injection drug users in Taiwan. Clin Infect Dis 46, 1761-1768, (2008).

420 Lemos, L. B. et al. Clinical and laboratory characteristics of acute hepatitis C in patients with end-stage renal disease on hemodialysis. J Clin Gastroenterol 42, 208-211, (2008).

421 Lemos, L. B. et al. Hepatitis C among predialysis patients: prevalence and characteristics in a large cohort of patients. Nephron Clin Pract 108, c135-140, (2008).

422 Lampe, E., Yoshida, C. F. T., De Oliveira, R. V., Lauer, G. M. & Lewis-Ximenez, L. L. Molecular analysis and patterns of ALT and hepatitis C virus seroconversion in haemodialysis patients with acute hepatitis. Nephrology (Carlton) 13, 186-192, (2008).

423 Ladero, J. M. et al. "12 weeks' stopping rule" in the treatment of genotype 1 chronic hepatitis C: two prognostic categories under the same label? Scand J Gastroenterol 43, 979-983, (2008).

424 Kurbanov, F. et al. Molecular epidemiology and interferon susceptibility of the natural recombinant hepatitis C virus strain RF1_2k/1b. J Infect Dis 198, 1448-1456, (2008).

425 Kuntzen, T. et al. Naturally occurring dominant resistance mutations to hepatitis C virus protease and polymerase inhibitors in treatment-naïve patients. Hepatology 48, 1769-1778, (2008).

426 Koutsounaki, E. et al. Mannose-binding lectin MBL2 gene polymorphisms and outcome of hepatitis C virus-infected patients. J Clin Immunol 28, 495-500, (2008).

427 Khan, A. et al. Epidemiological and clinical evaluation of hepatitis B, hepatitis C, and delta hepatitis viruses in Tajikistan. J. Med. Virol. 80, 268-276, (2008).

428 Katsarou, O. et al. Pegylated interferon plus ribavirin combination therapy for chronic hepatitis C in patients with congenital coagulation disorders. Acta Haematol 120, 63-69, (2008).

429 Kamal, S. & Nasser, I. Hepatitis C genotype 4: What we know and what we don't yet know. Hepatology 47, 1371-1383, (2008).

430 Jonsson, J., Barrie, H., O'Rourke, P., Clouston, A. & Powell, E. Obesity and steatosis influence serum and hepatic inflammatory markers in chronic hepatitis C. Hepatology 48, 80-87, (2008).

431 Jessner, W. et al. Very early viral kinetics on interferon treatment in chronic hepatitis C virus genotype 4 infection. Antivir Ther 13, 581-589, (2008).

432 Jamieson, D. J. et al. Infection with Hepatitis C Virus among HIV-Infected Pregnant Women in Thailand. Infect Dis Obstet Gynecol Article ID 840948, 7, (2008).

433 Idrees, M. & Riazuddin, S. Frequency distribution of hepatitis C virus genotypes in different geographical regions of Pakistan and their possible routes of transmission. BMC Infect Dis 8, 69, (2008).

434 Iacovazzi, P. A. et al. Serum 90K/Mac-2 binding protein (Mac-2BP) as a response predictor to peginterferon and ribavirin combined treatment in HCV chronic patients. Immunopharmacol Immunotoxicol 30, 687-700, (2008).

435 Hézode, C. et al. Daily Cannabis Use: A Novel Risk Factor of Steatosis Severity in Patients With Chronic Hepatitis C. Gastroenterology 134, 432-439, (2008).

436 Goodman, Z. D. et al. Pathology of chronic hepatitis C in children: liver biopsy findings in the Peds-C Trial. Hepatology 47, 836-843, (2008).

437 Gigi, E. et al. Cytokine mRNA expression in hepatitis C virus infection: TH1 predominance in patients with chronic hepatitis C and TH1-TH2 cytokine profile in subjects with self-limited disease. J Viral Hepat 15, 145-154, (2008).

438 Gao, D. et al. Assessment of specific antibodies to F protein in serum samples from Chinese hepatitis C patients treated with interferon plus ribavarin. J Clin Microbiol 46, 3746-3751, (2008).

439 Gad, R. et al. Predictors of a sustained virological response in patients with genotype 4 chronic hepatitis C. Liver International 28, 1112-1119, (2008).

440 Fried, R. et al. Swiss multicenter study evaluating the efficacy, feasibility and safety of peginterferon-alfa-2a and ribavirin in patients with chronic hepatitis C in official opiate substitution programs. Digestion 78, 123-130, (2008).

441 Fried, M. W. et al. Improved outcomes in patients with hepatitis C with difficult-to-treat characteristics: randomized study of higher doses of peginterferon alpha-2a and ribavirin. Hepatology 48, 1033-1043, (2008).

442 Freitas, S. Z. et al. Prevalence, genotypes and risk factors associated with hepatitis C virus infection in hemodialysis patients in Campo Grande, MS, Brazil. Mem Inst Oswaldo Cruz 103, 405-408, (2008).

443 Fortunato, G. & Calcagno…, G. Multiple sclerosis and hepatitis C virus infection are associated with single nucleotide polymorphisms in interferon pathway genes. J Interferon Cytokine Res 28, 141-152, (2008).

444 Floreani, A. et al. Pegylated interferon alpha-2b plus ribavirin for naive patients with HCV-related cirrhosis. J Clin Gastroenterol 42, 734-737, (2008).

445 Ferenci, P. et al. Silibinin is a potent antiviral agent in patients with chronic hepatitis C not responding to pegylated interferon/ribavirin therapy. Gastroenterology 135, 1561-1567, (2008).

446 Ferenci, P. et al. Peginterferon Alfa-2a and Ribavirin for 24 Weeks in Hepatitis C Type 1 and 4 Patients With Rapid Virological Response. Gastroenterology 135, 451 - 458, (2008).

447 Fabris, P., Baldo, V., Baldovin, T. & Bellotto…, E. Changing epidemiology of HCV and HBV infections in Northern Italy: a survey in the general population. J Clin Gastroenterol 42, 527-532, (2008).

448 Escudero, A. et al. Pegylated alpha-interferon-2a plus ribavirin compared with pegylated alpha-interferon-2b plus ribavirin for initial treatment of chronic hepatitis C virus: prospective, non-randomized study. Journal of Gastroenterology and Hepatology 23, 861-866, (2008).

449 Elefsiniotis, I. et al. Patient's age modifies the impact of the proposed predictors of sustained virological response in chronic hepatitis C patients treated with PEG-interferon plus ribavirin. Eur J Intern Med 19, 266-270, (2008).

450 Economou, M. et al. Baseline cholesterol is associated with the response to antiviral therapy in chronic hepatitis C. J Gastroenterol Hepatol 23, 586-591, (2008).

451 Dzekova-Vidimliski, P. et al. Patterns of viraemia in haemodialysis patients with hepatitis C. Prilozi 29, 201-211, (2008).

452 Djebbi, A. et al. Genetic variability of genotype 1 hepatitis C virus isolates from Tunisian haemophiliacs. New Microbiologica 31, 473-480, (2008).

453 Di Lello, F., Garcia, G., Kott, V., Sookoian, S. & Campos, R. Diversity of hepatitis C virus genotype 1b in Buenos Aires, Argentina: description of a new cluster associated with response to treatment. J. Med. Virol. 80, 619-627, (2008).

454 Desbois, D., Vaghefi, P., Savary, J., Dussaix, E. & Roque-Afonso, A.-M. Sensitivity of a rapid immuno-chromatographic test for Hepatitis C antibodies detection. Journal of Clinical Virology 41, 129-133, (2008).

455 D'Errico-Grigioni, A., Fiorentino, M. & Vasuri…, F. Tissue hepatitis C virus RNA quantification and protein expression help identify early hepatitis C virus recurrence after liver transplantation. Liver Transpl 14, 313-320, (2008).

456 Delladetsima, I. et al. Apoptosis and hepatitis C virus infection in renal transplant recipients. Am J Clin Pathol 129, 744-748, (2008).

457 Chong, V. & Zinna, H. Hepatitis C virus infection and haemodialysis: experience of a district general hospital in Brunei Darussalam. Singapore Med J 49, 916-920, (2008).

458 Chlabicz, S. et al. High prevalence of genotype 4 among hepatitis C virus-infected intravenous drug users in north-eastern Poland. J. Med. Virol. 80, 615-618, (2008).

459 Chlabicz, S. et al. Changing HCV genotypes distribution in Poland--relation to source and time of infection. J Clin Virol 42, 156-159, (2008).

460 Cavalheiro, N. d. P., Santos, A. C. d. O., Melo, C. E., Morimitsu, S. R. & Barone, A. A. Hepatitis C virus detection in the semen of infected patients. Braz J Infect Dis 12, 358-361, (2008).

461 Castelain, S. et al. Low Levels of Hepatitis C Virus (HCV) Neutralizing Antibodies in Patients Coinfected with HCV and Human Immunodeficiency Virus. Journal of Infectious Diseases 198, 332-335, (2008).

462 Cantaloube, J. et al. Molecular characterization of genotype 2 and 4 hepatitis C virus isolates in French blood donors. J Med Virol 80, 1732-1739, (2008).

463 Cakaloğlu, Y. et al. Prevalence and clinical significance of SEN-H virus in chronic hepatitis B, C and delta infections in Turkey. Turk J Gastroenterol 19, 104-108, (2008).

464 Botelho, S. M. et al. Epidemiological aspects of hepatitis C virus infection among renal transplant recipients in Central Brazil. Mem Inst Oswaldo Cruz 103, 472-476, (2008).

465 Borghi, V., Puoti, M., Mussini, C., Bellelli, S. & Angeletti…, C. HIV coinfection and antiretroviral therapy enhances liver steatosis in patients with hepatitis C, but only in those infected by HCV genotype other than 3. Antivir Ther 13, 1057-1065, (2008).

466 Bart, G. et al. Markers for hepatitis A, B and C in methadone maintained patients: an unexpectedly high co-infection with silent hepatitis B. Addiction 103, 681-686, (2008).

467 Barría, M. I. et al. Influence of extrahepatic viral infection on the natural history of hepatitis C. Ann Hepatol 7, 136-143, (2008).

468 Bain, V. G. et al. Clinical trial: exposure to ribavirin predicts EVR and SVR in patients with HCV genotype 1 infection treated with peginterferon alpha-2a plus ribavirin. Aliment Pharmacol Ther 28, 43-50, (2008).

469 Baatarkhuu, O. et al. Prevalence and genotype distribution of hepatitis C virus among apparently healthy individuals in Mongolia: a population-based nationwide study. Liver International 28, 1389-1395, (2008).

470 Arrais, T. C. et al. Change in hepatitis C virus genotype in hemodialysis patients after end-of-treatment response to interferon monotherapy--relapse or re-infection? J. Med. Virol. 80, 80-86, (2008).

471 Amador-Cañizares, Y. et al. Induction of IgA and sustained deficiency of cell proliferative response in chronic hepatitis C. World J Gastroenterol 14, 6844-6852, (2008).

472 Alves Pedroso, M. L. et al. Mannan-binding lectin MBL2 gene polymorphism in chronic hepatitis C: association with the severity of liver fibrosis and response to interferon therapy. Clin Exp Immunol 152, 258-264, (2008).

473 Altuglu, I., Soyler, I., Ozacar, T. & Erensoy, S. Distribution of hepatitis C virus genotypes in patients with chronic hepatitis C infection in Western Turkey. Int J Infect Dis 12, 239-244, (2008).

474 Akuta, N. et al. Efficacy of low-dose intermittent interferon-alpha monotherapy in patients infected with hepatitis C virus genotype 1b who were predicted or failed to respond to pegylated interferon plus ribavirin combination therapy. J. Med. Virol. 80, 1363-1369, (2008).

475 Akhan, S. C., Kalender, B. & Ruzgar, M. The Response to Pegylated Interferon Alpha 2a in Haemodialysis Patients with Hepatitis C Virus Infection. Infection 36, 341-344, (2008).

476 Yu, J., Wang, G., Sun, L., Li, X. & Li, S. Predictive value of rapid virological response and early virological response on sustained virological response in HCV patients treated with pegylated interferon alpha-2a and ribavirin. J Gastroenterol Hepatol 22, 832-836, (2007).

477 Witthöft, T. et al. Safety, tolerability and efficacy of peginterferon alpha-2a and ribavirin in chronic hepatitis C in clinical practice: The German Open Safety Trial. J Viral Hepat 14, 788-796, (2007).

478 Wietzke-Braun, P. et al. Spontaneous elimination of hepatitis C virus infection: a retrospective study on demographic, clinical, and serological correlates. World J Gastroenterol 13, 4224-4229, (2007).

479 Tsatsralt-Od, B. et al. Prevalence of hepatitis B, C, and delta virus infections among children in Mongolia: progress in childhood immunization. J Med Virol 79, 1064-1074, (2007).

480 Thomas, F. et al. Genetic diversity of HCV genotype 2 strains in south western France. J Med Virol 79, 26-34, (2007).

481 Tallo, T. et al. Genetic characterization of hepatitis C virus strains in Estonia: fluctuations in the predominating subtype with time. J Med Virol 79, 374-382, (2007).

482 Svirtlih, N. et al. Hepatitis C virus genotypes in Serbia and Montenegro: the prevalence and clinical significance. World J Gastroenterol 13, 355-360, (2007).

483 Sunanchaikarn, S. et al. Seroepidemiology and Genotypes of Hepatitis C Virus in Thailand. Asian Pac J Allegery Immunol 25, 175-182, (2007).

484 Stefanova-Petrova, D. et al. Chronic hepatitis C virus infection: prevalence of extrahepatic manifestations and association with cryoglobulinemia in Bulgarian patients. World J Gastroenterol 13, 6518-6528, (2007).

485 Siagris, D. et al. Serum adiponectin in chronic hepatitis C and B. J Viral Hepat 14, 577-583, (2007).

486 Sharvadze, L., Gochitashvili, N., Tophuria, A., Bolokadze, N. & Tsertsvadze, T. IFN/RBV treatment induced neutropenia and its correction with neupogen in patients with hepatitis C. Georgian Med News 147, 52-55, (2007).

487 Segat, L. et al. Association of polymorphisms in the first exon of mannose binding lectin gene (MBL2) in Brazilian patients with HCV infection. Clin Immunol 124, 13-17, (2007).

488 Schott, E. et al. A Toll-like receptor 7 single nucleotide polymorphism protects from advanced inflammation and fibrosis in male patients with chronic HCV-infection. J Hepatol 47, 203-211, (2007).

489 Schäfer, A., Scheurlen, M., Weissbrich, B., Schöttker, K. & Kraus, M. Sustained virological response in the antiviral therapy of chronic hepatitis C: is there a predictive value of interferon-induced depression? Chemotherapy 53, 292-299, (2007).

490 Schaefer, M. et al. Hepatitis C treatment in "difficult-to-treat" psychiatric patients with pegylated interferon-alpha and ribavirin: response and psychiatric side effects. Hepatology 46, 991-998, (2007).

491 Sarmento-Castro, R. et al. Impact of peginterferon alpha-2b and ribavirin treatment on liver tissue in patients with HCV or HCV-HIV co-infection. J Infect 54, 609-616, (2007).

492 Sánchez-Avila, J. F., González, E., Vázquez, V., Suárez, S. & Uribe, M. Geographical distribution of HCV genotypes in Mexico. Ann Hepatol 6, 156-160, (2007).

493 Samimi-Rad, K. & Shahbaz, B. Hepatitis C virus genotypes among patients with thalassemia and inherited bleeding disorders in Markazi province, Iran. Haemophilia 13, 156-163, (2007).

494 Rumi, M., Aghemo, A., D'Ambrosio, R. & Ronchi…, G. Lack of rapid virological response predicts interferon-alpha 2b/ribavirin therapy failure in HCV genotype 2 patients: a single-centre study. Antivir Ther 12, 1033-1040, (2007).

495 Roulot, D. et al. Epidemiological characteristics and response to peginterferon plus ribavirin treatment of hepatitis C virus genotype 4 infection. J Viral Hepat 14, 460-467, (2007).

496 Roe, B. et al. Elevated Serum Levels of Interferon-γ-Inducible Protein-10 in Patients Coinfected with Hepatitis C Virus and HIV. Journal of Infectious Diseases 196, 1053-1057, (2007).

497 Rodrigue-Gervais, I. G. et al. Poly(I:C) and lipopolysaccharide innate sensing functions of circulating human myeloid dendritic cells are affected in vivo in hepatitis C virus-infected patients. J Virol 81, 5537-5546, (2007).

498 Roccatello, D., Fornasieri, A., Giachino, O. & Rossi…, D. Multicenter Study on Hepatitis C Virus–Related Cryoglobulinemic Glomerulonephritis. Am J Kid Dis 49, 69-82, (2007).

499 Ré, V., Contigiani, M., Yoshida, C. F. T. & Lampe, E. Identification of hepatitis C virus subtype 2c by sequencing analysis in patients from Córdoba, Argentina. Mem Inst Oswaldo Cruz 102, 995-998, (2007).

500 Ramos-Casals, M. et al. Cryoglobulinaemia associated with hepatitis C virus: influence of HCV genotypes, HCV-RNA viraemia and HIV coinfection. J Viral Hepat 14, 736-742, (2007).

501 Ramos, B. et al. Changes in the distribution of hepatitis C virus (HCV) genotypes over time in Spain according to HIV serostatus: implications for HCV therapy in HCV/HIV-coinfected patients. J Infect 54, 173-179, (2007).

502 Raffa, G., Maimone, S., Cargnel, A. & Santantonio…, T. Analysis of occult hepatitis B virus infection in liver tissue of HIV patients with chronic hepatitis C. AIDS 21, 2171-2175, (2007).

503 Puato, M. et al. Does HCV infection have a more favourable outcome in Tanzanian people?: Data from the Lugalawa study. Digestive and Liver Disease 39, 891-892, (2007).

504 Prasad, L. et al. Cohort Profile: the Swiss Hepatitis C Cohort Study (SCCS). Int J Epidemiol 36, 731-737, (2007).

505 Posthouwer, D., Mauser-Bunschoten, E. P., Fischer, K., van Erpecum, K. J. & de Knegt, R. J. Significant liver damage in patients with bleeding disorders and chronic hepatitis C: non-invasive assessment of liver fibrosis using transient elastography. J Thromb Haemost 5, 25-30, (2007).

506 Plamondon, M. et al. Hepatitis C virus infection in Guinea-Bissau: a sexually transmitted genotype 2 with parenteral amplification? PLoS One 2, e372, (2007).

507 Pineda, J. A. et al. Influence of concomitant antiretroviral therapy on the rate of sustained virological response to pegylated interferon plus ribavirin in hepatitis C virus/HIV-coinfected patients. J Antimicrob Chemother 60, 1347-1354, (2007).

508 Pillai, V., Lee, W. M., Thiele, D. L. & Karandikar, N. J. Clinical responders to antiviral therapy of chronic HCV infection show elevated antiviral CD4+ and CD8+ T-cell responses. J Viral Hepat 14, 318-329, (2007).

509 Petit, J.-M. et al. Cell surface expression of LDL receptor in chronic hepatitis C: correlation with viral load. American Journal of Physiology - Endocrinology and Metabolism 293, E416-E420, (2007).

510 Persico, M., Capasso, M., Persico, E. & Svelto…, M. Suppressor of cytokine signaling 3 (SOCS3) expression and hepatitis C virus–related chronic hepatitis: insulin resistance and response to antiviral therapy. Hepatology 46, 1009-1015, (2007).

511 Pearlman, B. L., Ehleben, C. & Saifee, S. Treatment extension to 72 weeks of peginterferon and ribavirin in hepatitis c genotype 1-infected slow responders. Hepatology 46, 1688-1694, (2007).

512 Mussi, A. D. H., Pereira, R. A. R. d. A., Corrêa e Silva, V. d. A., Martins, R. M. B. & Souto, F. J. D. Epidemiological aspects of hepatitis C virus infection among HIV-infected individuals in Mato Grosso State, Central Brazil. Acta Trop 104, 116-121, (2007).

513 Mudawi, H. M. Y., Smith, H. M., Fletcher, I. A. & Fedail, S. S. Prevalence and common genotypes of HCV infection in Sudanese patients with hepatosplenic schistosomiasis. J. Med. Virol. 79, 1322-1324, (2007).

514 Moriondo, M., Resti, M., Betti, L. & Indolfi…, G. SEN virus co-infection among HCV-RNA-positive mothers, risk of transmission to the offspring and outcome of child infection during a 1-year follow-up. J Viral Hepat 14, 355-359, (2007).

515 Moratorio, G. et al. Evolution of naturally occurring 5'non-coding region variants of Hepatitis C virus in human populations of the South American region. Virol J 4, 79, (2007).

516 Masarone, M., La Mura, V. & Bruno…, S. Steatohepatitis is associated with diabetes and fibrosis in genotype 1b HCV-related chronic liver disease. J Viral Hepat 14, 714-720, (2007).

517 Marzouk, D. et al. Metabolic and cardiovascular risk profiles and hepatitis C virus infection in rural Egypt. Gut 56, 1105-1110, (2007).

518 Lurie, Y. et al. Acute hepatitis C in Israel: a predominantly iatrogenic disease? J Gastroenterol Hepatol 22, 158-164, (2007).

519 Lindh, M. et al. Response prediction and treatment tailoring for chronic hepatitis C virus genotype 1 infection. J Clin Microbiol 45, 2439-2445, (2007).

520 Łapinski, T. W., Wiercińska-Drapało, A., Panasiuk, A. & Kovalchuk, O. Concentrations of ssDNA in liver tissue and its correlation with sFas and sFasL in serum of patients infected with HBV, HCV, HCV and HIV. Adv Med Sci 52, 109-113, (2007).

521 Kumar, M. et al. Risk factors analysis for hepatocellular carcinoma in patients with and without cirrhosis: A case–control study of 213 hepatocellular carcinoma patients from India. Journal of Gastroenterology and Hepatology 22, 1104-1111, (2007).

522 Ksiaa, L. et al. Clearance and persistence of hepatitis C virus in a Tunisian population: association with HLA class I and class II. Viral Immunol 20, 312-319, (2007).

523 Kowala-Piaskowska, A., Mozer-Lisewska, I., Figlerowicz, M. & Słuzewski, W. Influence of the presence of HCV-RNA in peripheral blood mononuclear cells on the clinical course of chronic hepatitis C in children. European Journal of Epidemiology 22, 343-348, (2007).

524 Ivić, I. et al. Hla-Cw7 allele as predictor of favorable therapeutic response to interferon-alpha in patients with chronic hepatitis C. Croat Med J 48, 807-813, (2007).

525 Honda, T. et al. Efficacy of ribavirin plus interferon-α in patients aged ≥60 years with chronic hepatitis C. Journal of Gastroenterology and Hepatology 22, 989-995, (2007).

526 Hmaied, F. et al. Full-length genome sequences of hepatitis C virus subtype 4f. J Gen Virol 88, 2985-2990, (2007).

527 Hallinan, R., Byrne, A., Agho, K. & Dore, G. J. Referral for chronic hepatitis C treatment from a drug dependency treatment setting. Drug and Alcohol Dependence 88, 49 - 53, (2007).

528 Grebely, J. et al. Directly observed therapy for the treatment of hepatitis C virus infection in current and former injection drug users. Journal of Gastroenterology and Hepatology 22, 1519-1525, (2007).

529 Grebely, J. et al. Treatment uptake and outcomes among current and former injection drug users receiving directly observed therapy within a multidisciplinary group model for the treatment of hepatitis C virus infection. Int J Drug Policy 18, 437-443, (2007).

530 Ghafur, A. et al. Travel-associated acquisition of hepatitis C virus infection in patients receiving haemodialysis. Nephrol Dial Transplant 22, 2640-2644, (2007).

531 García-Montalvo, B. M. & Macossay-Castillo, M. Preliminary data for genotype distribution and epidemiological aspects of hepatitis C virus infection in blood donors from Yucatan, Mexico. Transfus Med 17, 488-490, (2007).

532 Galun, E. et al. Clinical evaluation (Phase I) of a human monoclonal antibody against hepatitis C virus: safety and antiviral activity. J Hepatol 46, 37-44, (2007).

533 Gabbay, E. et al. Antioxidant therapy for chronic hepatitis C after failure of interferon: results of phase II randomized, double-blind placebo controlled clinical trial. World J Gastroenterol 13, 5317-5323, (2007).

534 Esmat, G. et al. Evaluation of serum biomarkers of fibrosis and injury in Egyptian patients with chronic hepatitis C. J Hepatol 46, 620-627, (2007).

535 Elefsiniotis, I. S. et al. Impact of shorter duration of treatment on virological response rate in genotype 2 or 3 chronic hepatitis C virus infection. Indian J Gastroenterol 26, 209-212, (2007).

536 Economou, M. et al. Treatment and retreatment in patients with chronic hepatitis C: 10 years clinical practice in a single centre. Liver International 27, 340-346, (2007).

537 Doucette, K. E., Weinkauf, J., Sumner, S., Ens, K. & Lien, D. Treatment of hepatitis C in potential lung transplant candidates. Transplantation 83, 1652-1655, (2007).

538 Di Marco, V. et al. Peg-interferon alone or combined with ribavirin in HCV cirrhosis with portal hypertension: a randomized controlled trial. J Hepatol 47, 484-491, (2007).

539 de Paula Farah, K. et al. Hepatitis C, HCV genotypes and hepatic siderosis in patients with chronic renal failure on haemodialysis in Brazil. Nephrol Dial Transplant 22, 2027-2031, (2007).

540 Cursino-Santos, J. R., Donadi, E. A., Martinelli, A. L. C., Louzada-Junior, P. & Martinez-Rossi, N. M. Evolution of hepatitis C virus infection under host factor influence in an ethnically complex population. Liver Int 27, 1371-1378, (2007).

541 Cunha, L. et al. Use of replacement blood donors to study the epidemiology of major blood-borne viruses in the general population of Maputo, Mozambique. J. Med. Virol. 79, 1832-1840, (2007).

542 Cunha, L. et al. Use of replacement blood donors to study the epidemiology of major blood-borne viruses in the general population of Maputo, Mozambique. J. Med. Virol. 79, 1832-1840, (2007).

543 Crespo, M. et al. Peginterferon alpha-2b plus ribavirin vs interferon alpha-2b plus ribavirin for chronic hepatitis C in HIV-coinfected patients. J Viral Hepat 14, 228-238, (2007).

544 Cohen, M. et al. Development of Specific Antibodies to an ARF Protein in Treated Patients with Chronic HCV Infection. Dig Dis Sci 52, 2427-2432, (2007).

545 Cenci, M., Massi, M., Alderisio, M. & De Soccio…, G. Prevalence of hepatitis C virus (HCV) genotypes and increase of type 4 in central Italy: an update and report of a new method of HCV genotyping. Anticancer Res 27, 1219-1222, (2007).

546 Castelain, S. et al. Hepatitis C Virus p7 membrane protein quasispecies variability in chronically infected patients treated with interferon and ribavirin, with or without amantadine. J Med Virol 79, 144-154, (2007).

547 Carneiro, M. A. S. et al. Molecular and epidemiological study on nosocomial transmission of HCV in hemodialysis patients in Brazil. J. Med. Virol. 79, 1325-1333, (2007).

548 Bortolotti, F., Jorio, R., Resti, M., Cammà, C. & Marcellini…, M. Epidemiological profile of 806 Italian children with hepatitis C virus infection over a 15-year period. J Hepatol 46, 783-790, (2007).

549 Berzsenyi, M. D. et al. Reduction in Hepatitis C–Related Liver Disease Associated With \GB\ Virus C in Human Immunodeficiency Virus Coinfection. Gastroenterology 133, 1821 - 1830, (2007).

550 Bergmann, J. F. et al. Gamma-glutamyltransferase and rapid virological response as predictors of successful treatment with experimental or standard peginterferon-alpha-2b in chronic hepatitis C non-responders. Liver Int 27, 1217-1225, (2007).

551 Bárcena, R. et al. The magnitude of week 4 HCV RNA decay on pegylated interferon/ribavirin accurately predicts virological failure in patients with genotype 1. Antivir Ther 12, 401-406, (2007).

552 Antonucci, G. et al. The effect of age on response to therapy with peginterferon alpha plus ribavirin in a cohort of patients with chronic HCV hepatitis including subjects older than 65 yr. Am J Gastroenterol 102, 1383-1391, (2007).

553 Antonucci, G., Longo, M. & Angeletti…, C. 고령자에서 만성 C 형간염의 치료. Korean Journal of …, (2007).

554 Annicchiarico, B. E., Siciliano, M., Avolio, A. W., Grillo, R. L. & Bombardieri, G. A 5-year prospective study of the late resolution of chronic hepatitis C after antiviral therapy. Aliment Pharmacol Ther 25, 1039-1046, (2007).

555 Anis, S. et al. Cryoglobulinaemia and autoimmune markers in hepatitis C virus infected patients on renal replacement therapy. J Pak Med Assoc 57, 225-229, (2007).

556 Amarapurkar, D., Patel, N., Rane, P. & Kamani, P. Do different hepatitis C virus genotypes behave differently? Trop Gastroenterol 28, 99-104, (2007).

557 Akuta, N. et al. Amino acid substitutions in the hepatitis C virus core region are the important predictor of hepatocarcinogenesis. Hepatology 46, 1357-1364, (2007).

558 Ahlenstiel, G. et al. The GNB3 C825T polymorphism affects response to HCV therapy with pegylated interferon in HCV/HIV co-infected but not in HCV mono-infected patients. J Hepatol 47, 348-355, (2007).

559 Aberle, J. et al. CD4+ T Cell Responses in Patients with Chronic Hepatitis C Undergoing Peginterferon/Ribavirin Therapy Correlate with Faster, but Not Sustained, Viral Clearance. Journal of Infectious Diseases 195, 1315-1319, (2007).

560 ABERGEL, A. et al. The epidemiology and virology of hepatitis C virus genotype 5 in central France. Aliment Pharmacol Ther 26, 1437-1446, (2007).

561 Zhou, D. et al. Hepatitis C virus genotype distribution among intravenous drug user and the general population in Hong Kong. J. Med. Virol. 78, 574-581, (2006).

562 Zandieh, I., Adenwalla, M., Cheong-Lee, C., Ma, P. E. & Yoshida, E. M. Retinal vein thrombosis associated with pegylated-interferon and ribavirin combination therapy for chronic hepatitis C. World J Gastroenterol 12, 4908-4910, (2006).

563 Yenice, N., Mehtap, O., Gümrah, M. & Arican, N. The efficacy of pegylated interferon alpha 2a or 2b plus ribavirin in chronic hepatitis C patients. Turk J Gastroenterol 17, 94-98, (2006).

564 Vince, A. et al. Distribution of hepatitis C virus genotypes in Croatia-a 10 year retrospective study of four geographic regions. Coll Antropol 30, 139-143, (2006).

565 van de Laar, T. J. W. et al. Diversity and origin of hepatitis C virus infection among unpaid blood donors in the Netherlands. Transfusion 46, 1719-1728, (2006).

566 Uberti-Foppa, C., De Bona, A. & Galli…, L. Liver fibrosis in HIV-positive patients with hepatitis C virus: role of persistently normal alanine aminotransferase levels. JAIDS 41, 63-67, (2006).

567 Tsatsralt-Od, B. et al. Infection with hepatitis A, B, C, and delta viruses among patients with acute hepatitis in Mongolia. J Med Virol 78, 542-550, (2006).

568 Tsamandas, A. et al. Potential role of hepatic progenitor cells expression in cases of chronic hepatitis C and their relation to response to therapy: a clinicopathologic study. Liver Int 26, 817-826, (2006).

569 Toubi, E. et al. Elevated serum B-Lymphocyte activating factor (BAFF) in chronic hepatitis C virus infection: association with autoimmunity. J Autoimmun 27, 134-139, (2006).

570 Torti, C., Lapadula, G., Puoti, M. & Casari…, S. Influence of genotype 3 hepatitis C coinfection on liver enzyme elevation in HIV-1-positive patients after commencement of a new highly active antiretroviral regimen: …. JAIDS 41, 180-185, (2006).

571 Toro, C. et al. Molecular and epidemiological characteristics of blood-borne virus infections among recent immigrants in Spain. J. Med. Virol. 78, 1599-1608, (2006).

572 Tanaka, Y. et al. Molecular Tracing of the Global Hepatitis C Virus Epidemic Predicts Regional Patterns of Hepatocellular Carcinoma Mortality. Gastroenterology 130, 703 - 714, (2006).

573 Suneetha, P. V., Goyal, A., Hissar, S. S. & Sarin, S. K. Studies on TAQ1 polymorphism in the 3′untranslated region of IL-12P40 gene in HCV patients infected predominantly with genotype 3. J. Med. Virol. 78, 1055-1060, (2006).

574 Stikleryte, A. et al. Characterization of HCV strains in an oncohematological pediatric department reveals little horizontal transmission but multiple introductions by un-screened blood products in the past. J Med Virol 78, 1411-1422, (2006).

575 Simó, R., Lecube, A., Genescà, J., Esteban, J. I. & Hernández, C. Sustained virological response correlates with reduction in the incidence of glucose abnormalities in patients with chronic hepatitis C virus infection. Diabetes Care 29, 2462-2466, (2006).

576 Selcuk, H. et al. Distribution of HCV genotypes in patients with end-stage renal disease according to type of dialysis treatment. Dig Dis Sci 51, 1420-1425, (2006).

577 Sebastiani, G., Vario, A., Ferrari, A. & Pistis…, R. Hepatic iron, liver steatosis and viral genotypes in patients with chronic hepatitis C. J Viral Hepat 13, 199-205, (2006).

578 Sánchez-Tapias, J. M. et al. Peginterferon-alfa2a plus ribavirin for 48 versus 72 weeks in patients with detectable hepatitis C virus RNA at week 4 of treatment. Gastroenterology 131, 451-460, (2006).

579 Saadoun, D. et al. Cryoglobulinemia is associated with steatosis and fibrosis in chronic hepatitis C. Hepatology 43, 1337-1345, (2006).

580 Rumbo, C. et al. Hepatitis C in children: a quaternary referral center perspective. J Pediatr Gastroenterol Nutr 43, 209-216, (2006).

581 Picchio, G. R. et al. High prevalence of infection with a single hepatitis C virus genotype in a small rural community of Argentina. Liver Int 26, 660-665, (2006).

582 Petti, S. et al. Analysis of the shift of the transmission pattern for hepatitis C in a community in Central Italy. New Microbiol 29, 207-209, (2006).

583 Pereira, G. A. S. et al. Human immunodeficiency virus type 1 and hepatitis C virus co-infection and viral subtypes at an HIV testing center in Brazil. J. Med. Virol. 78, 719-723, (2006).

584 Panzer, S. et al. Platelet autoantibodies are common in hepatitis C infection, irrespective of the presence of thrombocytopenia. Eur J Haematol 77, 513-517, (2006).

585 Oyunsuren, T. et al. High frequency of hepatocellular carcinoma in Mongolia; association with mono-, or co-infection with hepatitis C, B, and delta viruses. J Med Virol 78, 1688-1695, (2006).

586 Noppornpanth, S. et al. Identification of a naturally occurring recombinant genotype 2/6 hepatitis C virus. J Virol 80, 7569-7577, (2006).

587 Nattermann, J. et al. The tandem-repeat polymorphism of the DC-SIGNR gene in HCV infection. J Viral Hepat 13, 42-46, (2006).

588 Morice, Y. et al. Molecular epidemiology of hepatitis C virus subtype 3a in injecting drug users. J. Med. Virol. 78, 1296-1303, (2006).

589 Mimidis, K. et al. Hepatitis C virus survival curve analysis in naive patients treated with peginterferon alpha-2b plus ribavirin. A randomized controlled trial for induction with high doses of peginterferon and predictability of sustained viral response from early virologic data. J Gastrointestin Liver Dis 15, 213-219, (2006).

590 Mihm, U. et al. Amino acid variations in hepatitis C virus p7 and sensitivity to antiviral combination therapy with amantadine in chronic hepatitis C. Antivir Ther 11, 507-519, (2006).

591 Meyer-Wyss, B. et al. Comparison of two PEG-interferon alpha-2b doses (1.0 or 1.5 microg/kg) combined with ribavirin in interferon-naïve patients with chronic hepatitis C and up to moderate fibrosis. J Viral Hepat 13, 457-465, (2006).

592 Méndez-Sánchez, N. et al. Risk factors and prevalence of hepatitis virus B and C serum markers among nurses at a tertiary-care hospital in Mexico City, Mexico: a descriptive study. Ann Hepatol 5, 276-280, (2006).

593 McGovern, B. H. et al. Hepatic steatosis is associated with fibrosis, nucleoside analogue use, and hepatitis C virus genotype 3 infection in HIV-seropositive patients. Clin Infect Dis 43, 365-372, (2006).

594 Martins, R. M. B. et al. Distribution of hepatitis C virus genotypes among blood donors from mid-west region of Brazil. Rev Inst Med Trop Sao Paulo 48, 53-55, (2006).

595 Maor, Y. et al. Hepatitis C at the israeli national hemophilia center. Haemophilia 12, 68-74, (2006).

596 Lopes, E. P. A. et al. Determination of the cut-off value of serum alanine aminotransferase in patients undergoing hemodialysis, to identify biochemical activity in patients with hepatitis C viremia. J Clin Virol 35, 298-302, (2006).

597 Livingston, S. E. et al. Steatosis and hepatitis C in an Alaska Native/American Indian population. Int J Circumpolar Health 65, 253-260, (2006).

598 Leandro, G. et al. Relationship between steatosis, inflammation, and fibrosis in chronic hepatitis C: a meta-analysis of individual patient data. Gastroenterology 130, 1636-1642, (2006).

599 Khaja, M. N. et al. High prevalence of hepatitis C virus infection and genotype distribution among general population, blood donors and risk groups. Infection, Genetics and Evolution 6, 198 - 204, (2006).

600 Karaca, Ç. et al. Risk Factors for the Transmission of Hepatitis C Virus Infection in the Turkish Population. Dig Dis Sci 51, 365-369, (2006).

601 Jittiwutikarn, J. et al. Hepatitis C infection among drug users in northern Thailand. Am J Trop Med Hyg 74, 1111-1116, (2006).

602 Hosseini-Moghaddam, S. et al. Distribution of hepatitis C virus genotypes among hemodialysis patients in Tehran—a multicenter study. J. Med. Virol. 78, 569-573, (2006).

603 Hopkins, S. et al. Role of individualization of hepatitis C virus (HCV) therapy duration in HIV/HCV-coinfected individuals*. HIV Medicine 7, 248-254, (2006).

604 Hofer, H., Gurguta, C., Bergholz, U., Steindl-Munda, P. & Ferenci, P. Standard interferon-alpha in combination with ribavirin for hepatitis C patients with advanced liver disease and thrombocytopenia. Wien Klin Wochenschr 118, 595-600, (2006).

605 Helbling, B. et al. HCV-related advanced fibrosis/cirrhosis: randomized controlled trial of pegylated interferon alpha-2a and ribavirin. J Viral Hepat 13, 762-769, (2006).

606 Halfon, P., Bourlière, M., Pénaranda, G., Khiri, H. & Ouzan, D. Real-time PCR assays for hepatitis C virus (HCV) RNA quantitation are adequate for clinical management of patients with chronic HCV infection. J Clin Microbiol 44, 2507-2511, (2006).

607 Gutierrez-Reyes, G. et al. Effect of pentoxifylline on levels of pro-inflammatory cytokines during chronic hepatitis C. Scand J Immunol 63, 461-467, (2006).

608 Fuster, D. et al. Results of a study of prolonging treatment with pegylated interferon-alpha2a plus ribavirin in HIV/HCV-coinfected patients with no early virological response. Antivir Ther 11, 473-482, (2006).

609 Fujiwara, K. et al. Twenty-four weeks of interferon α-2b in combination with ribavirin for Japanese hepatitis C patients: sufficient treatment period for patients with genotype 2 but not for patients with genotype 1. Liver International 26, 520-528, (2006).

610 Fernández-Rodríguez, C. M. et al. Long-term reversal of hypocholesterolaemia in patients with chronic hepatitis C is related to sustained viral response and viral genotype. Aliment Pharmacol Ther 24, 507-512, (2006).

611 Fernández-Arcás, N. et al. High prevalence of hepatitis C virus subtypes 4c and 4d in Malaga (Spain): phylogenetic and epidemiological analyses. J. Med. Virol. 78, 1429-1435, (2006).

612 Ferenci, P. et al. Randomized, double-blind, placebo-controlled study of peginterferon alfa-2a (40KD) plus ribavirin with or without amantadine in treatment-naïve patients with chronic hepatitis C genotype 1 infection. J Hepatol 44, 275-282, (2006).

613 Federici, A., Santagostino, E., Rumi, M. & Russo…, A. The natural history of hepatitis C virus infection in Italian patients with von Willebrand's disease: a cohort study. Haematologica 91, 503-508, (2006).

614 Donnerer, J. et al. Ribavirin Levels and Haemoglobin Decline in Early Virological Responders and Non-Responders to Hepatitis C Virus Combination Therapy. Pharmacology 76, 136-140, (2006).

615 Di Liberto, G. et al. Clinical and therapeutic implications of hepatitis C virus compartmentalization. Gastroenterology 131, 76-84, (2006).

616 Di Bisceglie, A. M., Fan, X., Chambers, T. & Strinko, J. Pharmacokinetics, pharmacodynamics, and hepatitis C viral kinetics during antiviral therapy: the null responder. J. Med. Virol. 78, 446-451, (2006).

617 Derbala, M. et al. Treatment of hepatitis C virus genotype 4 with peginterferon alfa-2a: impact of bilharziasis and fibrosis stage. World J Gastroenterol 12, 5692-5698, (2006).

618 de Vries, M. J., te Rijdt, B. & van Nieuwkerk, C. M. J. Genotype distribution amongst hepatitis C patients in The Netherlands. Neth J Med 64, 109-113, (2006).

619 Cornberg, M. et al. Treatment with daily consensus interferon (CIFN) plus ribavirin in non-responder patients with chronic hepatitis C: A randomized open-label pilot study. J Hepatol 44, 291-301, (2006).

620 Cooper, C. L., Al-Bedwawi, S., Lee, C. & Garber, G. Rate of infectious complications during interferon-based therapy for hepatitis C is not related to neutropenia. Clin Infect Dis 42, 1674-1678, (2006).

621 Conjeevaram, H. S. et al. Peginterferon and ribavirin treatment in African American and Caucasian American patients with hepatitis C genotype 1. Gastroenterology 131, 470-477, (2006).

622 Ciancio, A. et al. A randomized trial of pegylated-interferon-alpha2a plus ribavirin with or without amantadine in the re-treatment of patients with chronic hepatitis C not responding to standard interferon and ribavirin. Aliment Pharmacol Ther 24, 1079-1086, (2006).

623 Cantaloube, J. et al. Analysis of the 5' noncoding region versus the NS5b region in genotyping hepatitis C virus isolates from blood donors in France. J Clin Microbiol 44, 2051-2056, (2006).

624 Bronowicki, J. P. et al. Effect of Ribavirin in Genotype 1 Patients With Hepatitis C Responding to Pegylated Interferon Alfa-2a Plus Ribavirin. Gastroenterology 131, 1040-1048, (2006).

625 Bracho, M. A., Carrillo-Cruz, F. Y., Ortega, E., Moya, A. & Gonzalez-Candelas, F. A new subtype of hepatitis C virus genotype 1: complete genome and hylogenetic relationships of an Equatorial Guinea isolate. Journal of General Violgy 87, 1697-1702, (2006).

626 Biggar, R. J. et al. Hepatitis C virus genotype 4 in Ugandan children and their mothers. Emerg Infect Dis 12, 1440-1443, (2006).

627 Benetti, G., Borzio, M., Ramella, G. & Bellati…, G. Daily dose of interferon alpha-2b and ribavirin in treatment-naive patients with chronic hepatitis C virus genotype 1 infection: a randomised controlled study. Intern Emerg Med 1, 113-118, (2006).

628 Ben-Ari, Z. et al. Platelet-derived growth factor gene polymorphism in recurrent hepatitis C infection after liver transplantation. Transplantation 81, 392-397, (2006).

629 Barreiro, P. et al. Predictors of liver fibrosis in HIV-infected patients with chronic hepatitis C virus (HCV) infection: assessment using transient elastometry and the role of HCV genotype 3. Clin Infect Dis 42, 1032-1039, (2006).

630 Bain, V. G. et al. A phase 2 study to evaluate the antiviral activity, safety, and pharmacokinetics of recombinant human albumin-interferon alfa fusion protein in genotype 1 chronic hepatitis C patients. J Hepatol 44, 671-678, (2006).

631 Altindis, M., Yilmaz, S., Dikengil, T., Acemoglu, H. & Hosoglu, S. Seroprevalence and genotyping of hepatitis B, hepatitis C

and HIV among healthy population and Turkish soldiers in

Northern Cyprus World Journal of Gastroenterology 12, 6792-6796, (2006).

632 Al-Kubaisy, W. A., Al-Naib, K. T. & Habib, M. Seroprevalence of hepatitis C virus

specific antibodies among Iraqi

children with thalassaemia. Eastern Mediterranean Health Journal 12, 204-210, (2006).

633 Aghemo, A., Rumi, M., Soffredini, R. & D'Ambrosio…, R. Impaired response to interferon-alpha2b plus ribavirin in cirrhotic patients with genotype 3a hepatitis C virus infection. Antivir Ther 11, 797-802, (2006).

634 Aberle, J. et al. Prospective study of viral clearance and CD4(+) T-cell response in acute hepatitis C primary infection and reinfection. J Clin Virol 36, 24-31, (2006).

635 Zusinaite, E., Metskula, K. & Salupere, R. Autoantibodies and hepatitis C virus genotypes in chronic hepatitis C patients in Estonia. World J Gastroenterol 11, 488-491, (2005).

636 Wong, V.-K., Cheong-Lee, C., Ford, J.-A.-E. & Yoshida, E.-M. Acute sensorineural hearing loss associated with peginterferon and ribavirin combination therapy during hepatitis C treatment: outcome after resumption of therapy. World J Gastroenterol 11, 5392-5393, (2005).

637 von Wagner, M. et al. Peginterferon-alpha-2a (40KD) and ribavirin for 16 or 24 weeks in patients with genotype 2 or 3 chronic hepatitis C. Gastroenterology 129, 522-527, (2005).

638 Valenti, L. et al. TNFalpha genotype affects TNFalpha release, insulin sensitivity and the severity of liver disease in HCV chronic hepatitis. J Hepatol 43, 944-950, (2005).

639 Tsatsralt-Od, B. et al. High prevalence of hepatitis B, C and delta virus infections among blood donors in Mongolia. Arch Virol 150, 2513-2528, (2005).

640 Tsatsralt-Od, B. et al. High prevalence of dual or triple infection of hepatitis B, C, and delta viruses among patients with chronic liver disease in Mongolia. J Med Virol 77, 491-499, (2005).

641 Troesch, M. et al. Characterization of humoral and cell-mediated immune responses directed against hepatitis C virus F protein in subjects co-infected with hepatitis C virus and HIV-1. AIDS 19, 775-784, (2005).

642 Tahan, V. et al. Sexual transmission of HCV between spouses. Am J Gastroenterol 100, 821-824, (2005).

643 Syriopoulou, V. et al. Mother to child transmission of hepatitis C virus: Rate of infection and risk factors. Scand J Infect Dis 37, 350-353, (2005).

644 Sypsa, V. et al. Incidence and patterns of hepatitis C virus seroconversion in a cohort of hemodialysis patients. American Journal of Kidney Diseases 45, 334-343, (2005).

645 Sulkowski, M. S. et al. Hepatic steatosis and antiretroviral drug use among adults coinfected with HIV and hepatitis C virus. AIDS 19, 585-592, (2005).

646 Sominskaya, I. et al. Hepatitis B and C Virus Variants in Long-Term Immunosuppressed Renal Transplant Patients in Latvia. Intervirology 48, 192-200, (2005).

647 Simpore, J. et al. HCV and HIV co-infection in pregnant women attending St. Camille Medical Centre in Ouagadougou (Burkina Faso). J. Med. Virol. 75, 209-212, (2005).

648 Silva, G. F., Nishimura, N. F., Coelho, K. I. R. & Soares, E. C. Grading and staging chronic hepatitis C and its relation to genotypes and epidemiological factors in Brazilian blood donors. Braz J Infect Dis 9, 142-149, (2005).

649 Shustov, A. et al. Molecular epidemiology of the hepatitis C virus in Western Siberia. J Med Virol 77, 382-389, (2005).

650 Savvas, S. et al. Changes in epidemiological patterns of HCV infection and their impact on liver disease over the last 20 years in Greece. J Viral Hepat 12, 551-557, (2005).

651 Rigopoulou, E. et al. HCV-RNA qualitative assay based on transcription mediated amplification improves the detection of hepatitis C virus infection in patients on hemodialysis: Results from five hemodialysis units in central Greece. Journal of Clinical Virology 34, 81-85, (2005).

652 Richardson, M. et al. A combination of genetic polymorphisms increases the risk of progressive disease in chronic hepatitis C. J Med Genet 42, e45-e45, (2005).

653 Pham, T. N. Q. et al. Mitogen-induced upregulation of hepatitis C virus expression in human lymphoid cells. J Gen Virol 86, 657-666, (2005).

654 Payan, C. et al. Changing of hepatitis C virus genotype patterns in France at the beginning of the third millenium: The GEMHEP GenoCII Study. J Viral Hepat 12, 405-413, (2005).

655 Núnez, M. et al. Impact of ribavirin exposure on early virological response to hepatitis C therapy in HIV-infected patients with chronic hepatitis C. Antivir Ther 10, 657-662, (2005).

656 Njouom, R. et al. LOW RISK OF MOTHER-TO-CHILD TRANSMISSION OF HEPATITIS C VIRUS IN YAOUNDÉ, CAMEROON: THE ANRS 1262 STUDY. American Journal of Tropical Medicine and Hygiene 73, 460-466, (2005).

657 Nicot, F. et al. Heterogeneity of hepatitis C virus genotype 4 strains circulating in south-western France. J Gen Virol 86, 107-114, (2005).

658 Mosley, J. W. et al. Viral and host factors in early hepatitis C virus infection. Hepatology 42, 86-92, (2005).

659 Moreno, A. et al. HCV clearance and treatment outcome in genotype 1 HCV-monoinfected, HIV-coinfected and liver transplanted patients on peg-IFN-alpha-2b/ribavirin. J Hepatol 43, 783-790, (2005).

660 Monto, A. et al. Hepatic steatosis in HIV/hepatitis C coinfection: prevalence and significance compared with hepatitis C monoinfection. Hepatology 42, 310-316, (2005).

661 Méndez-Sánchez, N. et al. Prevalence of hepatitis C infection in a population of asymptomatic people in a checkup unit in Mexico city. Dig Dis Sci 50, 733-737, (2005).

662 Melhem, A. et al. Treatment of chronic hepatitis C virus infection via antioxidants: results of a phase I clinical trial. J Clin Gastroenterol 39, 737-742, (2005).

663 Mejri, S. et al. Contrasting patterns of hepatitis C virus infection in two regions from Tunisia. J. Med. Virol. 76, 185-193, (2005).

664 Meiler, C. et al. Different effects of a CD14 gene polymorphism on disease outcome in patients with alcoholic liver disease and chronic hepatitis C infection. World J Gastroenterol 11, 6031-6037, (2005).

665 McHutchison, J. G. et al. A randomized, double-blind, placebo-controlled dose-escalation trial of merimepodib (VX-497) and interferon-alpha in previously untreated patients with chronic hepatitis C. Antivir Ther 10, 635-643, (2005).

666 McGovern, B. et al. Delivering therapy for hepatitis C virus infection to incarcerated HIV-seropositive patients. Clin Infect Dis 41 Suppl 1, S56-62, (2005).

667 Mangia, A., Santoro, R., Minerva, N. & Ricci…, G. Peginterferon alfa-2b and ribavirin for 12 vs. 24 weeks in HCV genotype 2 or 3. NEJM 352, 2609-2617, (2005).

668 Mangia, A., Ricci, G., Persico, M. & Minerva…, N. A randomized controlled trial of pegylated interferon α-2a (40 KD) or interferon α-2a plus ribavirin and amantadine vs interferon α-2a and ribavirin in treatment-naïve …. J Viral Hepat 12, 292-299, (2005).

669 Maida, I. et al. Characteristics and prospects for hepatitis C therapy of an HIV-HCV coinfected population followed at a reference HIV center. HIV Clinical Trials 6, 329-336, (2005).

670 Macedo de Oliveira, A. et al. An outbreak of hepatitis C virus infections among outpatients at a hematology/oncology clinic. Ann Intern Med 142, 898-902, (2005).

671 Lu, L. et al. Hepatitis C virus genotype distribution in China: Predominance of closely related subtype 1b isolates and existence of new genotype 6 variants. J. Med. Virol. 75, 538-549, (2005).

672 Longman, R. S., Talal, A. H., Jacobson, I. M., Rice, C. M. & Albert, M. L. Normal functional capacity in circulating myeloid and plasmacytoid dendritic cells in patients with chronic hepatitis C. J Infect Dis 192, 497-503, (2005).

673 Lackner, C. et al. Comparison and validation of simple noninvasive tests for prediction of fibrosis in chronic hepatitis C. Hepatology 41, 1376-1382, (2005).

674 Keating, S., Coughlan, S., Connell, J., Sweeney, B. & Keenan, E. Hepatitis C viral clearance in an intravenous drug-using cohort in the Dublin area. Irish J Med Sci 174, 37-41, (2005).

675 Jiao, J. & Wang, J. B. Hepatitis C virus genotypes, HLA-DRB alleles and their response to interferon-alpha and ribavirin in patients with chronic hepatitis C. Hepatobiliary Pancreat Dis Int 4, 80-83, (2005).

676 Iorio, R., Giannattasio, A. & Sepe…, A. Chronic hepatitis C in childhood: an 18-year experience. Clin Infect Dis 41, 1431-1437, (2005).

677 Huber, M. et al. Interferon alpha-2a plus ribavirin 1,000/1,200 mg versus interferon alpha-2a plus ribavirin 600 mg for chronic hepatitis C infection in patients on opiate maintenance treatment: an open-label randomized multicenter trial. Infection 33, 25-29, (2005).

678 Hass, H.-G., Kreysel, C., Fischinger, J., Menzel, J. & Kaiser, S. High-dose interferon-alpha2b induction therapy in combination with ribavirin for treatment of chronic hepatitis C in patients with non-response or relapse after interferon-alpha monotherapy. World J Gastroenterol 11, 5342-5346, (2005).

679 Grassi, A. et al. HCV liver infection and liver steatosis: evidence for indirect mechanisms in genotype 3? Aliment Pharmacol Ther 22 Suppl 2, 79-82, (2005).

680 Gérard, C. et al. Evolution over a 10 year period of the epidemiological profile of 1,726 newly diagnosed HCV patients in Belgium. J Med Virol 76, 503-510, (2005).

681 Gatselis, N. et al. Impact of parietal cell autoantibodies and non-organ-specific autoantibodies on the treatment outcome of patients with hepatitis C virus infection: a pilot study. World J Gastroenterol 11, 482-487, (2005).

682 Garten, R. et al. Coinfection with HIV and hepatitis C virus among injection drug users in southern China. Clin Infect Dis 41 Suppl 1, S18-24, (2005).

683 Gallegos-Orozco, J. F. et al. Early hepatitis C virus changes and sustained response in patients with chronic hepatitis C treated with peginterferon alpha-2b and ribavirin. Liver Int 25, 91-95, (2005).

684 Fartoux, L. et al. Insulin resistance is a cause of steatosis and fibrosis progression in chronic hepatitis C. Gut 54, 1003-1008, (2005).

685 El Gaafary, M. et al. Surveillance of acute hepatitis C in Cairo, Egypt. J. Med. Virol. 76, 520-525, (2005).

686 Dutoit, V., Ciuffreda, D., Comte, D., Gonvers, J.-J. & Pantaleo, G. Differences in HCV-specific T cell responses between chronic HCV infection and HIV/HCV co-infection. Eur J Immunol 35, 3493-3504, (2005).

687 Dove, L., Phung, Y., Bzowej, N. & Kim…, M. Viral evolution of hepatitis C in injection drug users. J Viral Hepat 12, 574-583, (2005).

688 Desombere, I., Van Vlierberghe, H., Couvent, S., Clinckspoor, F. & Leroux-Roels, G. Comparison of qualitative (COBAS AMPLICOR HCV 2.0 versus VERSANT HCV RNA) and quantitative (COBAS AMPLICOR HCV monitor 2.0 versus VERSANT HCV RNA 3.0) assays for hepatitis C virus (HCV) RNA detection and quantification: impact on diagnosis and treatment of HCV infections. J Clin Microbiol 43, 2590-2597, (2005).

689 Dai, C. Y. et al. Co-infection of SENV-D among chronic hepatitis C patients treated with combination therapy with high-dose interferon-alfa and ribavirin. World Journal of Gastroenterology 11, 4241-4245, (2005).

690 Cusumano, A. M., Poratto, F., del Pino, N., Fernández, J. L. & Vilches, A. Identification of hepatitis C virus RNA in peritoneal dialysis fluid of patients with viremia. Perit Dial Int 25, 478-482, (2005).

691 Cristina, J. et al. Hepatitis C virus F protein sequence reveals a lack of functional constraints and a variable pattern of amino acid substitution. J Gen Virol 86, 115-120, (2005).

692 Clouston, A. et al. Fibrosis correlates with a ductular reaction in hepatitis C: roles of impaired replication, progenitor cells and steatosis. Hepatology 41, 809-818, (2005).

693 Cital-Zamora, J. L., Navarrete-Castro, R., Magaña-Muñoz, K. D., Martinez-Rodríguez, M. L. & Calderon, G. M. Prevalence of hepatitis C virus genotypes in a Mexican population. Arch Med Res 36, 607, (2005).

694 Chaudhuri, S. et al. Molecular epidemiology of \HCV\ infection among acute and chronic liver disease patients in Kolkata, India. Journal of Clinical Virology 32, 38 - 46, (2005).

695 Cargnel, A. et al. Open, randomized, multicentre italian trial on PEG-IFN plus ribavirin versus PEG-IFN monotherapy for chronic hepatitis C in HIV-coinfected patients on HAART. Antivir Ther 10, 309-317, (2005).

696 Cantaloube, J. et al. Genotype distribution and molecular epidemiology of hepatitis C virus in blood donors from southeast France. J Clin Microbiol 43, 3624-3629, (2005).

697 Campiotto, S. et al. Geographic distribution of hepatitis C virus genotypes in Brazil. Braz J Med Biol Res 38, 41-49, (2005).

698 Butera, D. et al. Plasma chemokine levels correlate with the outcome of antiviral therapy in patients with hepatitis C. Blood 106, 1175-1182, (2005).

699 Bortolotti, F., Resti, M., Marcellini, M. & Giacchino…, R. Hepatitis C virus (HCV) genotypes in 373 Italian children with HCV infection: changing distribution and correlation with clinical features and outcome. Gut 54, 852-857, (2005).

700 Barbosa, V. S., Silva, N. A. d. & Martins, R. M. B. Hepatitis C virus seroprevalence and genotypes in patients with diffuse connective tissue diseases and spondyloarthropathies. Braz J Med Biol Res 38, 801-805, (2005).

701 Aranzabal, L. et al. Influence of liver fibrosis on highly active antiretroviral therapy-associated hepatotoxicity in patients with HIV and hepatitis C virus coinfection. Clin Infect Dis 40, 588-593, (2005).

702 Ansaldi, F., Bruzzone, B. & Salmaso…, S. Different seroprevalence and molecular epidemiology patterns of hepatitis C virus infection in Italy. J Med Virol, (2005).

703 Alfonso, V., Mbayed, V. A., Sookoian, S. & Campos, R. H. Intra-host evolutionary dynamics of hepatitis C virus E2 in treated patients. J Gen Virol 86, 2781-2786, (2005).

704 Albuquerque, A. C. C. d., Coêlho, M. R. C., Lopes, E. P., Lemos, M. F. & Moreira, R. C. Prevalence and risk factors of hepatitis C virus infection in hemodialysis patients from one center in Recife, Brazil. Mem Inst Oswaldo Cruz 100, 467-470, (2005).

705 Adinolfi, L., Ingrosso, D., Cesaro, G. & Cimmino…, A. Hyperhomocysteinemia and the MTHFR C677T polymorphism promote steatosis and fibrosis in chronic hepatitis C patients. Hepatology 41, 995-1003, (2005).

706 ABONYI, M. & LAKATOS, P. Ribavirin in the Treatment of Hepatitis C. Anticancer Res 25, 1315-1320, (2005).

707 Yin, L. M. et al. Association of interleukin-12 p40 gene 3'-untranslated region polymorphism and outcome of HCV infection. World J Gastroenterol 10, 2330-2333, (2004).

708 Vogler, I. H. et al. Serological, epidemiological and molecular aspects of hepatitis C virus infection in a population from Londrina, PR, Brazil, 2001-2002. Rev Inst Med Trop Sao Paulo 46, 303-308, (2004).

709 Takahashi, M. et al. High prevalence of antibodies to hepatitis A and E viruses and viremia of hepatitis B, C, and D viruses among apparently healthy populations in Mongolia. Clin Diagn Lab Immunol 11, 392-398, (2004).

710 Sulkowski, M. S. et al. Daily versus thrice-weekly interferon alfa-2b plus ribavirin for the treatment of chronic hepatitis C in HIV-infected persons: a multicenter randomized controlled trial. JAIDS Journal of Acquired Immune Deficiency Syndromes 35, 464-472, (2004).

711 Sud, A. et al. Improved prediction of fibrosis in chronic hepatitis C using measures of insulin resistance in a probability index. Hepatology 39, 1239-1247, (2004).

712 Stroffolini, T., Colloredo, G. & Gaeta…, G. Does an 'autoimmune'profile affect the clinical profile of chronic hepatitis C? An Italian multicentre survey. J Viral Hepat 11, 257-262, (2004).

713 Stieltjes, N. et al. Interest of transjugular liver biopsy in adult patients with haemophilia or other congenital bleeding disorders infected with hepatitis C virus. Br J Haematol 125, 769-776, (2004).

714 Soza, A. et al. Clinical and epidemiological features of 147 Chilean patients with chronic hepatitis C. Ann Hepatol 3, 146-151, (2004).

715 Siagris, D. et al. Virological, immunological and histological aspects in adult β-thalassemic patients with chronic hepatitis C virus infection. Liver International 24, 204-209, (2004).

716 Siagris, D. et al. Cryoglobulinemia and progression of fibrosis in chronic HCV infection: cause or effect? Journal of Infection 49, 236-241, (2004).

717 Shinji, T. et al. Analysis of HCV genotypes from blood donors shows three new HCV type 6 subgroups exist in Myanmar. Acta Med Okayama 58, 135-142, (2004).

718 Shiffman, M. L. et al. Peginterferon alfa-2a and ribavirin in patients with chronic hepatitis C who have failed prior treatment. Gastroenterology 126, 1015-1023; discussion 1947, (2004).

719 Sharma, P. et al. Hepatic steatosis in hepatitis C virus genotype 3 infection: does it correlate with body mass index, fibrosis, and HCV risk factors? Dig Dis Sci 49, 25-29, (2004).

720 Samimi-Rad, K., Nategh, R., Malekzadeh, R., Norder, H. & Magnius, L. Molecular epidemiology of hepatitis C virus in Iran as reflected by phylogenetic analysis of the NS5B region. J Med Virol 74, 246-252, (2004).

721 Rouet, F. et al. HBV and HCV prevalence and viraemia in HIV-positive and HIV-negative pregnant women in Abidjan, Cote d'Ivoire: the ANRS 1236 study. J Med Virol 74, 34-40, (2004).

722 Qadi, A. et al. Hepatitis B and hepatitis C virus prevalence among dialysis patients in Bahrain and Saudi Arabia: a survey by serologic and molecular methods. Am J Infect Control 32, 493-495, (2004).

723 Puoti, M., Zanini, B., Quinzan, G. & Ravasio…, L. A randomized, controlled trial of triple antiviral therapy as initial treatment of chronic hepatitis C in HIV-infected patients. J Hepatol 41, 312-318, (2004).

724 Pradat, P. et al. The predictive value of core antigen testing for the management of hepatitis C patients receiving pegylated interferon/ribavirin treatment. J Med Virol 73, 392-396, (2004).

725 Poo, J. L. et al. Triple combination of thymalfasin, peginterferon alfa-2a and ribavirin in patients with chronic hepatitis C who have failed prior interferon and ribavirin treatment: 24-week interim results of a pilot study. Journal of Gastroenterology and Hepatology 19 Suppl 6, S79-81, (2004).

726 Peffault de Latour, R. et al. Long-term outcome of hepatitis C infection after bone marrow transplantation. Blood 103, 1618-1624, (2004).

727 Patton, H. M. et al. The impact of steatosis on disease progression and early and sustained treatment response in chronic hepatitis C patients. J Hepatol 40, 484-490, (2004).

728 Neumann, U. et al. Fibrosis progression after liver transplantation in patients with recurrent hepatitis C. J Hepatol 41, 830-836, (2004).

729 Nakano, T., Lu, L., Liu, P. & Pybus, O. G. Viral gene sequences reveal the variable history of hepatitis C virus infection among countries. J Infect Dis 190, 1098-1108, (2004).

730 Méndez-Sánchez, N. et al. Prevalence of hepatitis C virus infection among hemodialysis patients at a tertiary-care hospital in Mexico City, Mexico. J Clin Microbiol 42, 4321-4322, (2004).

731 McMahon, B. J. et al. Epidemiology and risk factors for hepatitis C in Alaska Natives. Hepatology 39, 325-332, (2004).

732 McDougall, N., McCluggage, W., Coyle, P., Sloan, J. & Callender, M. Early experience with chronic hepatitis C in Northern Ireland: epidemiology and response to monotherapy. Ulster Med J 73, 25-31, (2004).

733 MASCHERETTI, S. et al. Genetic variants in the CCR gene cluster and spontaneous viral elimination in hepatitis C-infected patients. Clinical & Experimental Immunology 136, 328-333, (2004).

734 Martial, J. et al. Hepatitis C virus (HCV) genotypes in the Caribbean island of Martinique: evidence for a large radiation of HCV-2 and for a recent introduction from Europe of HCV-4. J Clin Microbiol 42, 784-791, (2004).

735 Lyra, A. C., Ramrakhiani, S., Bacon, B. R. & Di Bisceglie, A. M. Infection with hepatitis C virus genotype 4 in the United States. J Clin Gastroenterol 38, 68-71, (2004).

736 López-Labrador, F. X. et al. Genetic variability of hepatitis C virus non-structural protein 3 and virus-specific CD8+ response in patients with chronic hepatitis C. J. Med. Virol. 72, 575-585, (2004).

737 Lehmann, M. et al. High rate of spontaneous clearance of acute hepatitis C virus genotype 3 infection. J Med Virol 73, 387-391, (2004).

738 Hultgren, C. et al. Evidence for a relation between the viral load and genotype and hepatitis C virus-specific T cell responses. J Hepatol 40, 971-978, (2004).

739 Hézode, C., Roudot-Thoraval, F., Zafrani, E.-S., Dhumeaux, D. & Pawlotsky, J.-M. Different mechanisms of steatosis in hepatitis C virus genotypes 1 and 3 infections. J Viral Hepat 11, 455-458, (2004).

740 HENQUELL, C. et al. High prevalence of hepatitis C virus type 5 in central France evidenced by a prospective study from 1996 to 2002. J Clin Microbiol 42, 3030-3035, (2004).

741 Gismondi, M. I., Turazza, E. I., Grinstein, S., Galoppo, M. C. & Preciado, M. V. Hepatitis C virus infection in infants and children from Argentina. J Clin Microbiol 42, 1199-1202, (2004).

742 Fernández, J. L. et al. Influence of hemodialysis procedure on HCV RNA detection in serum and peripheral blood mononuclear cells. Ren Fail 26, 369-373, (2004).

743 Fan, L. Y. et al. Cytotoxic T lymphocyte associated antigen-4 gene polymorphisms confer susceptibility to primary biliary cirrhosis and autoimmune hepatitis in Chinese population. World J Gastroenterol 10, 3056-3059, (2004).

744 Engler, S. et al. Interferon alfa2a induction therapy in combination with ribavirin and amantadine for the treatment of naive patients with chronic HCV infection. J Viral Hepat 11, 60-68, (2004).

745 de Almeida, A. J. et al. Hepatitis C virus-associated thrombocytopenia: a controlled prospective, virological study. Ann Hematol 83, 434-440, (2004).

746 Dalgard, O. et al. Treatment with pegylated interferon and ribavarin in HCV infection with genotype 2 or 3 for 14 weeks: a pilot study. Hepatology 40, 1260-1265, (2004).

747 Colina, R. et al. Evidence of intratypic recombination in natural populations of hepatitis C virus. J Gen Virol 85, 31-37, (2004).

748 Castéra, L. et al. Effect of antiviral treatment on evolution of liver steatosis in patients with chronic hepatitis C: indirect evidence of a role of hepatitis C virus genotype 3 in steatosis. Gut 53, 420-424, (2004).

749 Castellino, S. et al. The epidemiology of chronic hepatitis C infection in survivors of childhood cancer: an update of the St Jude Children's Research Hospital hepatitis C seropositive cohort. Blood 103, 2460-2466, (2004).

750 Campisi, G., Di Fede, O., Craxì, A. & Di Stefano…, R. Oral lichen planus, hepatitis C virus, and HIV: no association in a cohort study from an area of high hepatitis C virus endemicity. J Am Acad Dermatol 51, 364-370, (2004).

751 Bruno, S., Cammà, C., Di Marco, V., Rumi, M. & Vinci…, M. Peginterferon alfa-2b plus ribavirin for naive patients with genotype 1 chronic hepatitis C: a randomized controlled trial. J Hepatol 41, 474-481, (2004).

752 Brojer, E. et al. The hepatitis C virus genotype and subtype frequency in hepatitis C virus RNA-positive, hepatitis C virus antibody-negative blood donors identified in the nucleic acid test screening program in Poland. Transfusion 44, 1706-1710, (2004).

753 Bozdayı, A. et al. Molecular epidemiology of hepatitis B, C and D viruses in Turkish patients. Arch Virol 149, 2115-2129, (2004).

754 Ben-Ari, Z. et al. Role of cytokine gene polymorphism and hepatic transforming growth factor β1 expression in recurrent hepatitis C after liver transplantation. Cytokine 27, 7-14, (2004).

755 Andriulli, A. et al. Treatment of patients with HCV infection with or without liver biopsy. J Viral Hepat 11, 536-542, (2004).

756 Amorim, R. M. S. et al. Hepatitis C virus genotypes in blood donors from the Federal District, Central Brazil. Mem Inst Oswaldo Cruz 99, 895-897, (2004).

757 Alfonso, V., Flichman, D. M., Sookoian, S., Mbayed, V. A. & Campos, R. H. Evolutionary study of HVR1 of E2 in chronic hepatitis C virus infection. J Gen Virol 85, 39-46, (2004).

758 Alfaleh, F. et al. Peginterferon α-2b plus ribavirin compared with interferon α-2b plus ribavirin for initial treatment of chronic hepatitis C in Saudi patients commonly infected with genotype 4. Liver International 24, 568-574, (2004).

759 Aitken, C. et al. Molecular Epidemiology of Hepatitis C Virus in a Social Network of Injection Drug Users. Journal of Infectious Diseases 190, 1586-1595, (2004).

760 Airoldi, A. et al. Lack of a strong association between HLA class II, tumour necrosis factor and transporter associated with antigen processing gene polymorphisms and virological response to alpha-interferon treatment in patients with chronic hepatitis C. Eur J Immunogenet 31, 259-265, (2004).

761 Agwale, S. M. et al. Prevalence of \HCV\ coinfection in HIV-infected individuals in Nigeria and characterization of \HCV\ genotypes. Journal of Clinical Virology 31, Supplement 1, 3 - 6, (2004).

762 Torres, M. C. M. R. et al. Hepatitis C virus infection in a Brazilian population with sickle-cell anemia. Braz J Med Biol Res 36, 323-329, (2003).

763 Tassopoulos, N. et al. A randomized trial to assess the efficacy of interferon alpha in combination with ribavirin in the treatment of interferon alpha nonresponders with chronic hepatitis C: superior efficacy of high daily dosage of interferon alpha in genotype 1. J Viral Hepat 10, 189-196, (2003).

764 Tarantino, G. et al. Low daily dosage of interferon for 1 year after HCV-related end-therapy response. A randomized-controlled study. Liver Int 23, 413-419, (2003).

765 Tamalet, C. et al. Genomic and phylogenetic analysis of hepatitis C virus isolates: a survey of 535 strains circulating in southern France. J Med Virol 71, 391-398, (2003).

766 Strasfeld, L., Lo, Y., Netski, D., Thomas, D. L. & Klein, R. S. The association of hepatitis C prevalence, activity, and genotype with HIV infection in a cohort of New York City drug users. JAIDS Journal of Acquired Immune Deficiency Syndromes 33, 356-364, (2003).

767 Steindl-Munda, P. et al. Impact of high-dose interferon induction and ribavirin therapy in patients with chronic hepatitis C relapsing after or not responding to interferon monotherapy. Liver Int 23, 269-275, (2003).

768 Squadrito, G., Previti, M., Lenzi, M. & Le Rose…, E. High prevalence of non-organ-specific autoantibodies in hepatitis C virus-infected cirrhotic patients from southern Italy. Dig Dis Sci 48, 349-353, (2003).

769 Souza, K. P. et al. Hepatitis B and C in the hemodialysis unit of Tocantins, Brazil: serological and molecular profiles. Mem Inst Oswaldo Cruz 98, 599-603, (2003).

770 Siagris, D. et al. Viraemia, cryoglobulins and autoantibodies in haemodialysis patients infected with hepatitis C virus. European Journal of Gastroenterology & Hepatology 15, (2003).

771 Shobokshi, O., Serebour, F. E. & Skakni, L. I. Hepatitis C genotypes/subtypes among chronic hepatitis patients in

Saudi Arabia. Saudi Med J 24, S87-S91, (2003).

772 Serra, M. A., Rodríguez, F., del Olmo, J. A., Escudero, A. & Rodrigo, J. M. Influence of age and date of infection on distribution of hepatitis C virus genotypes and fibrosis stage. J Viral Hepat 10, 183-188, (2003).

773 Sanyal, A. J. et al. Nonalcoholic fatty liver disease in patients with hepatitis C is associated with features of the metabolic syndrome. Am J Gastroenterol 98, 2064-2071, (2003).

774 Romero-Gómez, M. et al. Serum leptin levels correlate with hepatic steatosis in chronic hepatitis C. Am J Gastroenterol 98, 1135-1141, (2003).

775 Quarleri, J. F. et al. In vitro detection of dissimilar amounts of hepatitis C virus (HCV) subtype-specific RNA genomes in mixes prepared from sera of persons infected with a single HCV genotype. J Clin Microbiol 41, 2727-2733, (2003).

776 Pizot-Martin, I. et al. Efficacy and Tolerance of HCV Treatment in HIV-HCV Coinfected Patients: The Potential Interaction of PI Treatment. HIV Clinical Trials 4, 262-268, (2003).

777 Petit, J. et al. Hepatitis C virus-associated hypobetalipoproteinemia is correlated with plasma viral load, steatosis, and liver fibrosis. Am J Gastroenterol 98, 1150-1154, (2003).

778 Perez, R. M. et al. Unexpected distribution of hepatitis C virus genotypes in patients on hemodialysis and kidney transplant recipients. J. Med. Virol. 69, 489-494, (2003).

779 Nolte, F. S. et al. Clinical evaluation of two methods for genotyping hepatitis C virus based on analysis of the 5' noncoding region. J Clin Microbiol 41, 1558-1564, (2003).

780 Nevens, F. et al. A pilot study of therapeutic vaccination with envelope protein \E1\ in 35 patients with chronic hepatitis C. Hepatology 38, 1289 - 1296, (2003).

781 Müller, Z. et al. Hepatitis C virus genotypes in Hungarian and Austrian patients with chronic hepatitis C. Journal of Clinical Virology 26, 295-300, (2003).

782 Mühlbauer, M. et al. A novel MCP-1 gene polymorphism is associated with hepatic MCP-1 expression and severity of HCV-related liver disease. Gastroenterology 125, 1085-1093, (2003).

783 Mele, A., Pulsoni, A., Bianco, E. & Musto…, P. Hepatitis C virus and B-cell non-Hodgkin lymphomas: an Italian multicenter case-control study. Blood 102, 996-999, (2003).

784 Layden-Almer, J. E., Ribeiro, R. M., Wiley, T., Perelson, A. S. & Layden, T. J. Viral dynamics and response differences in HCV-infected African American and white patients treated with IFN and ribavirin. Hepatology 37, 1343-1350, (2003).

785 Kurbanov, F. et al. Hepatitis C virus molecular epidemiology in Uzbekistan. J. Med. Virol. 69, 367-375, (2003).

786 Kumar, D. et al. Effectiveness of interferon alfa-2b/ribavirin combination therapy for chronic hepatitis C in a clinic setting. Medical Journal of Australia 178, 267-271, (2003).

787 Kamar, N. et al. Long-term ribavirin therapy in hepatitis C virus-positive renal transplant patients: effects on renal function and liver histology. American Journal of Kidney Diseases 42, 184-192, (2003).

788 Jessner, W. et al. Early viral kinetics on treatment with pegylated interferon-α-2a in chronic hepatitis C virus genotype 1 infection*. J Viral Hepat 10, 37-42, (2003).

789 Januszkiewicz-Lewandowska, D. et al. Transmission of HCV infection among long-term hospitalized onco-haematological patients. Journal of Hospital Infection 53, 120-123, (2003).

790 Hofer, H. et al. Spontaneous viral clearance in patients with acute hepatitis C can be predicted by repeated measurements of serum viral load. Hepatology 37, 60-64, (2003).

791 Hickman, I. J. et al. In overweight patients with chronic hepatitis C, circulating insulin is associated with hepatic fibrosis: implications for therapy. J Hepatol 39, 1042 - 1048, (2003).

792 Haushofer, A. et al. Genotyping of hepatitis C virus-comparison of three assays. J Clin Virol 27, 276-285, (2003).

793 Hartman, C., Berkowitz, D., Rimon, N. & Shamir, R. The effect of early treatment in children with chronic hepatitis. J Pediatr Gastroenterol Nutr 37, 252-257, (2003).

794 Giordano, H. M. et al. Chronic liver disease in kidney recipients with hepatitis C virus infection. Clin Transplant 17, 195-199, (2003).

795 Gehrke, S. et al. Hemochromatosis and transferrin receptor gene polymorphisms in chronic hepatitis C: impact on iron status, liver injury and HCV genotype. J Mol Med 81, 780-787, (2003).

796 Flichman, D., Kott, V., Sookoian, S. & Campos, R. Acute hepatitis C in a chronically HIV-infected patient: evolution of different viral genomic regions. World J Gastroenterol 9, 1496-1500, (2003).

797 Fargion, S., Bruno, S., Borzio, M. & Battezzati…, P. Sustained response to combination therapy in patients with chronic hepatitis C who failed to respond to interferon. J Hepatol 38, 499-505, (2003).

798 Fabris, P., Tositti, G. & Giordani…, M. Prevalence and clinical significance of circulating cryoglobulins in HIV-positive patients with and without Co-infection with hepatitis C virus. J Med Virol 69, 339-343, (2003).

799 Erhardt, A. et al. HFE mutations and chronic hepatitis C: H63D and C282Y heterozygosity are independent risk factors for liver fibrosis and cirrhosis. J Hepatol 38, 335-342, (2003).

800 Dumpis, U. et al. An outbreak of HBV and HCV infection in a paediatric oncology ward: epidemiological investigations and prevention of further spread. J Med Virol 69, 331-338, (2003).

801 Djebbi, A. et al. Genotypes of hepatitis C virus circulating in Tunisia. Epidemiology & Infection 130, 501-505 M503 - 510.1017/S095026880300846X, (2003).

802 Ding, X. et al. Molecular epidemiology of hepatitis viruses and genotypic distribution of hepatitis B and C viruses in Harbin, China. Jpn J Infect Dis 56, 19-22, (2003).

803 De Cock, L. & Vranckx, R. Serotyping and genotyping of hepatitis C virus in Belgium. Infection 31, 92-97, (2003).

804 Dalgard, O., Jeansson, S., Skaug, K., Raknerud, N. & Bell, H. Hepatitis C in the general adult population of Oslo: prevalence and clinical spectrum. Scand J Gastroenterol 38, 864-870, (2003).

805 Corbet, S., Bukh, J., Heinsen, A. & Fomsgaard, A. Hepatitis C virus subtyping by a core-envelope 1-based reverse transcriptase PCR assay with sequencing and its use in determining subtype distribution among Danish patients. J Clin Microbiol 41, 1091-1100, (2003).

806 Codes, L. et al. Comparative study of hepatitis C virus genotypes 1 and 3 in Salvador, Bahia Brazil. Braz J Infect Dis 7, 409-417, (2003).

807 Chowdhury, A. et al. Hepatitis C virus infection in the general population: A community-based study in West Bengal, India. Hepatology 37, 802-809, (2003).

808 Chopra, K. B. et al. Progression of liver fibrosis in patients with chronic hepatitis C after orthotopic liver transplantation. Transplantation 76, 1487-1491, (2003).

809 Chironna, M. et al. Prevalence Rates of Viral Hepatitis Infections in Refugee Kurds from Iraq and Turkey. Infection 31, 70-74, (2003).

810 Candotti, D., Temple, J., Sarkodie, F. & Allain, J. Frequent recovery and broad genotype 2 diversity characterize hepatitis C virus infection in Ghana, West Africa. J Virol 77, 7914-7923, (2003).

811 C., N. et al. Effect of Antiviral Therapy on Markers of Fibrogenesis in Patients with Chronic Hepatitis C. Scand J Gastroenterol 38, 659-665, (2003).

812 Buffet-Janvresse, C. et al. HIV and HCV co-infection: situation at six French university hospitals in the year 2000. J Med Virol 69, 7-17, (2003).

813 Berg, T. et al. Triple therapy with amantadine in treatment-naive patients with chronic hepatitis C: a placebo-controlled trial. Hepatology 37, 1359-1367, (2003).

814 Balogun, M. A. et al. A national survey of genitourinary medicine clinic attenders provides little evidence of sexual transmission of hepatitis C virus infection. Sex Transm Infect 79, 301-306, (2003).

815 Balciuniene, L., Carlsson, T., Ambrozaitis, A., Reichard, O. & Weiland, O. Hepatitis C Virus RNA Levels During Initial Induction or Standard Interferon Therapy: Influence of Continued Treatment on Sustained Response. Scand J Infect Dis 35, 180-185, (2003).

816 Ahlenstiel, G. et al. Effects of the CCR5-Δ32 mutation on antiviral treatment in chronic hepatitis C. J Hepatol 39, 245-252, (2003).

817 Zuckerman, E. et al. Peripheral B-cell CD5 expansion and CD81 overexpression and their association with disease severity and autoimmune markers in chronic hepatitis C virus infection. Clin Exp Immunol 128, 353-358, (2002).

818 Zhang, C. et al. High Prevalence of HIV-1 and Hepatitis C Virus Coinfection Among Injection Drug Users in the Southeastern Region of Yunnan, China. JAIDS Journal of Acquired Immune Deficiency Syndromes 29, (2002).

819 Woitas, R. et al. Frequency of the HIV-protective CC chemokine receptor 5-Delta32/Delta32 genotype is increased in hepatitis C. Gastroenterology 122, 1721-1728, (2002).

820 Westin, J. et al. Moderate alcohol intake increases fibrosis progression in untreated patients with hepatitis C virus infection. J Viral Hepat 9, 235-241, (2002).

821 Verbaan, H. P., Widell, H. E. A., Bondeson, T. L., Lindgren, S. C. & Group, S. H. T. High sustained response rate in patients with histologically mild (low grade and stage) chronic hepatitis C infection. A randomized, double blind, placebo controlled trial of interferon alpha-2b with and without ribavirin. European Journal of Gastroenterology & Hepatology 14, 627-633, (2002).

822 Vardas, E., Ross, M., Sharp, G., McAnerney, J. & Sim, J. Viral hepatitis in South African healthcare workers at increased risk of occupational exposure to blood-borne viruses. Journal of Hospital Infection 50, 6 - 12, (2002).

823 Van Vlierberghe, H. et al. Non-transferrin-bound iron in untreated and ribavirin-treated chronic hepatitis C patients. Aliment Pharmacol Ther 16, 1555-1562, (2002).

824 Tassopoulos, N. et al. IFN-alpha2b monotherapy in patients with chronic hepatitis C and persistently normal or near normal aminotransferase activity: a randomized, controlled study. J Interferon Cytokine Res 22, 365-369, (2002).

825 Taliani, G., Badolato, M., Nigro, G., Biasin, M. & Boddi…, V. Serum concentration of γGT is a surrogate marker of hepatic TNF-α mRNA expression in chronic hepatitis C. Clin Immunol 105, 279-285, (2002).

826 Siagris, D. et al. Keratoconjunctivitis Sicca and Chronic HCV Infection. Infection 30, 229-233, (2002).

827 Sherman, K. E., Rouster, S. D., Chung, R. T. & Rajicic, N. Hepatitis C Virus prevalence among patients infected with Human Immunodeficiency Virus: a cross-sectional analysis of the US adult AIDS Clinical Trials Group. Clin Infect Dis 34, 831-837, (2002).

828 Schulman, S., Kinnman, N., Lindmarker, P. & von Sydow, M. A randomized study of alpha-interferon plus ribavirin for 6 months or 12 months for the treatment of chronic hepatitis C in patients with bleeding disorders. Haemophilia 8, 129-135, (2002).

829 Reynolds, W. F. et al. A genotypic association implicates myeloperoxidase in the progression of hepatic fibrosis in chronic hepatitis C virus infection. Genes Immun 3, 345-349, (2002).

830 Resti, M., Azzari, C., Moriondo, M. & Betti…, L. Injection drug use facilitates hepatitis C virus infection of peripheral blood mononuclear cells. Clin Infect Dis 35, 236-239, (2002).

831 Renou, C. et al. Histological features and HLA class II alleles in hepatitis C virus chronically infected patients with persistently normal alanine aminotransferase levels. Gut 51, 585-590, (2002).

832 Puoti, C., Castellacci, R., Montagnese, F. & Zaltron…, S. Histological and virological features and follow-up of hepatitis C virus carriers with normal aminotransferase levels: the Italian prospective study of the asymptomatic C …. J Hepatol 37, 117-123, (2002).

833 Pereira, L. M. M. B. et al. Chronic hepatitis C infection: influence of the viral load, genotypes, and GBV-C/HGV coinfection on the severity of the disease in a Brazilian population. J. Med. Virol. 67, 27-32, (2002).

834 Passos, E. P. et al. Hepatitis C virus infection and assisted reproduction. Hum Reprod 17, 2085-2088, (2002).

835 Otagiri, H. et al. Evaluation of a new assay for hepatitis C virus genotyping and viral load determination in patients with chronic hepatitis C. J Virol Methods 103, 137 - 143, (2002).

836 Ortiz, V., Berenguer, M., Rayón, J. M., Carrasco, D. & Berenguer, J. Contribution of obesity to hepatitis C-related fibrosis progression. Am J Gastroenterol 97, 2408-2414, (2002).

837 Ndjomou, J. et al. Hepatitis C virus infection and genotypes among human immunodeficiency virus high-risk groups in Cameroon. J Med Virol 66, 179-186, (2002).

838 Matera, G. et al. Changes in the prevalence of hepatitis C virus (HCV) genotype 4 in Calabria, Southern Italy. Diagn Microbiol Infect Dis 42, 169-173, (2002).

839 Mangia, A., Santoro, R., Piattelli, M. & Leandro…, G. High doses of interferon in combination with ribavirin are more effective than the standard regimen in patients with HCV genotype 1 chronic hepatitis. J Hepatol 37, 109-116, (2002).

840 Mahmood, M. et al. Detection of Hepatitis C Virus (HCV) RNA in Normal Cervical Smears of HCV-Seropositive Patients. Clinical Infectious Diseases 35, 966-973, (2002).

841 Larke, B. et al. Acute nosocomial HCV infection detected by NAT of a regular blood donor. Transfusion 42, 759-765, (2002).

842 Lagging, L. M. et al. Progression of fibrosis in untreated patients with hepatitis C virus infection. Liver 22, 136-144, (2002).

843 Kramer, L. et al. Subclinical impairment of brain function in chronic hepatitis C infection. J Hepatol 37, 349 - 354, (2002).

844 Kalinina, O., Norder, H., Mukomolov, S. & Magnius, L. A natural intergenotypic recombinant of hepatitis C virus identified in St. Petersburg. J Virol 76, 4034-4043, (2002).

845 Ikeda, K. et al. Influence of Hepatitis C Virus Subtype on Hepatocellular Carcinogenesis: A Multivariate Analysis of a Retrospective Cohort of 593 Patients with Cirrhosis. Intervirology 45, 71-78, (2002).

846 Hüe, S. et al. Human leukocyte antigen class II alleles may contribute to the severity of hepatitis C virus-related liver disease. J Infect Dis 186, 106-109, (2002).

847 Hinrichsen, H. et al. Prevalence and risk factors of hepatitis C virus infection in haemodialysis patients: a multicentre study in 2796 patients. Gut 51, 429-433, (2002).

848 Helbling, D., Breitbach, T. H. & Krause, M. Disseminated cytomegalovirus infection in Crohn's disease following anti-tumour necrosis factor therapy. European Journal of Gastroenterology & Hepatology 14, 1393-1395, (2002).

849 Hanuka, N. et al. Hepatitis C virus infection in renal failure patients in the absence of anti-hepatitis C virus antibodies. J Viral Hepat 9, 141-145, (2002).

850 Halfon, P. et al. Serological response to infection with different isolates of hepatitis C virus. J Viral Hepat 9, 438-442, (2002).

851 Di Stefano, R., Stroffolini, T. & Ferraro…, D. Endemic hepatitis C virus infection in a Sicilian town: further evidence for iatrogenic transmission. J Med Virol 67, 339-344, (2002).

852 Deignan, T. et al. Decrease in hepatic CD56(+) T cells and V alpha 24(+) natural killer T cells in chronic hepatitis C viral infection. J Hepatol 37, 101-108, (2002).

853 de Lédinghen, V. et al. Daily or three times per week interferon α-2b in combination with ribavirin or interferon alone for the treatment of patients with chronic hepatitis C not responding to previous interferon alone. J Hepatol 36, 819-826, (2002).

854 Davoren, A. et al. Outcome of an optional HCV screening program for blood transfusion recipients in Ireland. Transfusion 42, 1501-1506, (2002).

855 Dal Molin, G., D'Agaro, P. & Ansaldi…, F. Mother-to-infant transmission of hepatitis C virus: Rate of infection and assessment of viral load and IgM anti-HCV as risk factors*. J Med Virol 67, 137-142, (2002).

856 Dal Molin, G., Ansaldi, F. & Biagi…, C. Changing molecular epidemiology of hepatitis C virus infection in Northeast Italy. J Med Virol 68, 352-356, (2002).

857 da Silva, L. C. et al. High rate of sustained response to consensus interferon plus ribavirin in chronic hepatitis C patients resistant to alpha-interferon and ribavirin: a pilot study. Journal of Gastroenterology 37, 732-736, (2002).

858 Crippin, J. S., McCashland, T., Terrault, N., Sheiner, P. & Charlton, M. R. A pilot study of the tolerability and efficacy of antiviral therapy in hepatitis C virus-infected patients awaiting liver transplantation. Liver Transpl 8, 350-355, (2002).

859 Cochrane, A. et al. A Genetic Analysis of Hepatitis C Virus Transmission between Injection Drug Users. Journal of Infectious Diseases 186, 1212-1221, (2002).

860 Carmo, R. A. et al. Hepatitis C virus infection among Brazilian hemophiliacs: a virological, clinical and epidemiological study. Braz J Med Biol Res 35, 589-598, (2002).

861 Campello, C., Poli, A., Dal Molin, G. & Besozzi-Valentini, F. Seroprevalence, viremia and genotype distribution of hepatitis C virus: a community-based population study in northern Italy. Infection 30, 7-12, (2002).

862 Busek, S. U. et al. Hepatitis C and hepatitis B virus infection in different hemodialysis units in Belo Horizonte, Minas Gerais, Brazil. Mem Inst Oswaldo Cruz 97, 775-778, (2002).

863 Bourlière, M. et al. Epidemiological changes in hepatitis C virus genotypes in France: evidence in intravenous drug users. J Viral Hepat 9, 62-70, (2002).

864 Bjøro, K. et al. Effect of combined interferon-alpha induction therapy and ribavirin on chronic hepatitis C virus infection: a randomized multicentre study. Scand J Gastroenterol 37, 226-232, (2002).

865 Bdour, S. Hepatitis C virus infection in Jordanian haemodialysis units: serological diagnosis and genotyping. J Med Microbiol 51, 700-704, (2002).

866 Bassit, L. et al. Comparative study of two anti-HCV screening tests in a large genotyped population of Brazilian dialysis patients. Eur J Clin Microbiol Infect Dis 21, 404-406, (2002).

867 Balogun, M. A. et al. The prevalence of hepatitis C in England and Wales. J Infect 45, 219-226, (2002).

868 Ansari, N., Ahmed, A., Esmail, J. & Mujeeb, A. HCV Serotypes in Karachi: a Liaquat National Hospital

Experience Journal of Pakistan Medical Association 71, (2002).

869 Al-Kubaisy, W. A., Niazi, A. D. & Kubba, K. History of miscarriage as a risk factor for hepatitis C virus infection in pregnant Iraqi women. Eastern Mediterranean Health Journal 8, 239-244, (2002).

870 Alberti, A., Noventa, F. & Benvegnù…, L. Prevalence of liver disease in a population of asymptomatic persons with hepatitis C virus infection. Ann Intern Med 137, 961-964, (2002).

871 Akuta, N. et al. Efficacy of interferon monotherapy to 394 consecutive naive cases infected with hepatitis C virus genotype 2a in Japan: therapy efficacy as consequence of tripartite interaction of viral, host and interferon treatment-related factors. J Hepatol 37, 831 - 836, (2002).

872 Accapezzato, D., Fravolini, F. & Casciaro…, M. Hepatitis C flare due to superinfection by genotype 4 in an HCV genotype 1b chronic carrier. European journal of … 14, 879-881, (2002).

873 Zuckerman, E. et al. bcl-2 and immunoglobulin gene rearrangement in patients with hepatitis C virus infection. Br J Haematol 112, 364-369, (2001).

874 Yerly, S. et al. Nosocomial Outbreak of Multiple Bloodborne Viral Infections. Journal of Infectious Diseases 184, 369-372, (2001).

875 Weinstein, T. et al. Hepatitis C inflection in dialysis patients in Israel. Israel Med Assoc J 3, 174-177, (2001).

876 Vlierberghe, H. V., Delanghe, J., Vos, M. D. & Leroux-Roel, G. Factors influencing ribavirin-induced hemolysis. J Hepatol 34, 911 - 916, (2001).

877 van Doornum, G. J., Lodder, A., Buimer, M., van Ameijden, E. J. & Bruisten, S. Evaluation of hepatitis C antibody testing in saliva specimens collected by two different systems in comparison with HCV antibody and HCV RNA in serum. J. Med. Virol. 64, 13-20, (2001).

878 Sullivan, D. G., Kim, S. S., Wilson, J. J., Stehman-Breen, C. & Gretch, D. R. Investigating hepatitis C virus heterogeneity in a high prevalence setting using heteroduplex tracking analysis. J Virol Methods 96, 5-16, (2001).

879 Serfaty, L. et al. Hepatitis C virus induced hypobetalipoproteinemia: a possible mechanism for steatosis in chronic hepatitis C. J Hepatol 34, 428-434, (2001).

880 Schinkel, J., Kroes, A. C., Wagtmans, M. J., Lamers, C. B. & van Hoek, B. Monitoring response during a randomised controlled trial of escalating interferon dose for chronic hepatitis C infection: predictive value of quantitative and qualitative HCV RNA assays. J Clin Virol 22, 61-71, (2001).

881 Saracco, G., Ciancio, A., Ghisetti, V. & Rocca…, G. Treatment with interferon-[alpha] 2b of naive non-cirrhotic patients with chronic hepatitis C according to viraemia and genotype. Results of a randomized multicentre …. Eur J Gastroenterol Hepatol 13, 149-155, (2001).

882 Sandres, K., Dubois, M., Pasquier, C., Puel, J. & Izopet, J. Determination of HCV Genotype Using Two Antibody Assays and Genome Typing. Eur J Clin Microbiol Infect Dis 20, 666-669, (2001).

883 Sagnelli, E., Coppola, N. & Scolastico…, C. CV genotype and “silent” HBV coinfection: Two main risk factors for a more severe liver disease. J Med Virol 64, 350-355, (2001).

884 Saginur, R. et al. Transmission of hepatitis C in a pharmacologic study. Infect Control Hosp Epidemiol 22, 697-700, (2001).

885 Rubbia-Brandt, L. et al. Liver steatosis in chronic hepatitis C: a morphological sign suggesting infection with HCV genotype 3. Histopathology 39, 119-124, (2001).

886 Rossini, A. et al. Hepatitis C virus (HCV) RNA determination after two weeks of induction interferon treatment is an accurate predictor of nonresponse: comparison of two treatment schedules. Dig Dis Sci 46, 2389-2395, (2001).

887 Riestra, S. et al. Prevalence of hepatitis C virus infection in the general population of northern Spain. European Journal of Gastroenterology & Hepatology 13, 477-481, (2001).

888 Rendina, D., Vigorita, E., Bonavolta, R. & D'Onofrio…, M. HCV and GBV-c/HGV infection in HIV positive patients in southern Italy. European Journal of Epidemiology 17, 801-807, (2001).

889 Raptopoulou-Gigi, M., Orphanou, E., Lalla, T., Lita, A. & 0Garifallos, A. Prevalence of hepatitis C virus infection in a cohort of pregnant women in northern Greece and transmission of HCV from mother to child. European Journal of Epidemiology 17, 263-266, (2001).

890 Queneau, P.-E. et al. Treatment of mild chronic hepatitis C with interferon α-2b: results of a multi-centre randomized study in 80 patients. European Journal of Gastroenterology & Hepatology 13, (2001).

891 Petit, J. et al. Risk factors for diabetes mellitus and early insulin resistance in chronic hepatitis C. J Hepatol 35, 279-283, (2001).

892 Oubiña, J. R. et al. Hepatitis C virus and GBV-C/hepatitis G virus in Argentine patients with porphyria cutanea tarda. Intervirology 44, 215-218, (2001).

893 Nakano, I. et al. Interferon responsiveness in patients infected with hepatitis C virus 1b differs depending on viral subtype. Gut 49, 263-267, (2001).

894 Nakai, K., Win, K., Oo, S., Arakawa, Y. & Abe, K. Molecular characteristic-based epidemiology of hepatitis B, C, and E viruses and GB virus C/hepatitis G virus in Myanmar. J Clin Microbiol 39, 1536-1539, (2001).

895 Mohsen, A. H. & Group, T. H. S. The epidemiology of hepatitis C in a UK health regional population of 5.12 million. Gut 48, 707-713, (2001).

896 Mihály, I. et al. Prevalence, genotype distribution and outcome of hepatitis C infections among the employees of the Hungarian Central Hospital for Infectious Diseases. Journal of Hospital Infection 49, 239-244, (2001).

897 Mangia, A., Minerva, N., Annese, M. & Leandro…, G. A randomized trial of amantadine and interferon versus interferon alone as initial treatment for chronic hepatitis C. Hepatology 33, 989-993, (2001).

898 Lee, S. S. & Sherman, M. Pilot study of interferon-alpha and ribavirin treatment in patients with chronic hepatitis C and normal transaminase values. J Viral Hepat 8, 202-205, (2001).

899 Krekulova, L. et al. Genotypic and epidemiologic characteristics of hepatitis C virus infections among recent injection drug user and nonuser populations. Clin Infect Dis 33, 1435-1438, (2001).

900 Jover, R. et al. Infection by genotype 5a of HCV in a district of southeast Spain. Am J Gastroenterol 96, 3042-3043, (2001).

901 Hwang, S. et al. Hepatic steatosis in chronic hepatitis C virus infection: prevalence and clinical correlation. J Gastroenterol Hepatol 16, 190-195, (2001).

902 Hervé, S. et al. Chronic hepatitis C with normal or abnormal aminotransferase levels: is it the same entity? Eur J Gastroenterol Hepatol 13, 495-500, (2001).

903 Haushofer, A. C., Kopty, C., Hauer, R., Brunner, H. & Halbmayer, W.-M. \HCV\ genotypes and age distribution in patients of Vienna and surrounding areas. Journal of Clinical Virology 20, 41 - 47, (2001).

904 Halfon, P. et al. Hepatitis C virus genotyping based on 5' noncoding sequence analysis (Trugene). J Clin Microbiol 39, 1771-1773, (2001).

905 Gürsoy, M., Gür, G., Arslan, H., Özdemir, N. & Boyacıogˇlu, S. Interferon therapy in haemodialysis patients with acute hepatitis C virus infection and factors that predict response to treatment. J Viral Hepat 8, 70-77, (2001).

906 Grahovac, B. et al. Dynamics of serum hepatitis C virus load and quasispecies complexity during antiviral therapy in patients with chronic hepatitis C. Journal of Clinical Virology 20, 85 - 89, (2001).

907 Franchini, M., Rossetti, G. & Tagliaferri…, A. The natural history of chronic hepatitis C in a cohort of HIV-negative Italian patients with hereditary bleeding disorders. Blood 98, 1836-1841, (2001).

908 Fontaine, H. et al. Hepatitis activity index is a key factor in determining the natural history of chronic hepatitis C. Hum Pathol 32, 904-909, (2001).

909 Ferenci, P. et al. Treatment of patients with chronic hepatitis C not responding to interferon with high-dose interferon alpha with or without ribavirin: final results of a prospective randomized trial. European Journal of Gastroenterology & Hepatology 13, (2001).

910 Ferenci, P. et al. Combination of interferon induction therapy and ribavirin in chronic hepatitis C. Hepatology 34, 1006-1011, (2001).

911 Desenclos, J. et al. Hepatitis C in a ward for cystic fibrosis and diabetic patients: possible transmission by spring-loaded finger-stick devices for self-monitoring of capillary blood glucose. Infect Control Hosp Epidemiol 22, 701-707, (2001).

912 de-Moreau-de-Gerbehaye, A. I., Bodeus, M. & Goubau, P. Age trend in hepatitis C virus genotype distribution as seen in a Brussels teaching hospital. Acta Clin Belg 56, 220-224, (2001).

913 de Oliveira, J. M. et al. Differences in HCV antibody patterns in haemodialysis patients infected with the same virus isolate. J. Med. Virol. 63, 265-270, (2001).

914 Damen, M. et al. Sustained virological response in chronic hepatitis C patients after a 6- and a 36-month interferon-alpha2b treatment schedule: a multicenter, randomized, controlled study. Scand J Gastroenterol 36, 97-104, (2001).

915 Chu, C. et al. Clinical, virologic, and pathologic significance of elevated serum alpha-fetoprotein levels in patients with chronic hepatitis C. J Clin Gastroenterol 32, 240-244, (2001).

916 Ceci, O. et al. Vertical transmission of hepatitis C virus in a cohort of 2,447 HIV-seronegative pregnant women: a 24-month prospective study. J Pediatr Gastroenterol Nutr 33, 570-575, (2001).

917 Castro, F. J. et al. Evaluation of hepatitis C virus RNA RT/PCR qualitative and quantitative second generation assays. J Virol Methods 91, 51-58, (2001).

918 Bekkering, F. C., Stalgis, C., McHutchison, J. G., Brouwer, J. T. & Perelson, A. S. Estimation of early hepatitis C viral clearance in patients receiving daily interferon and ribavirin therapy using a mathematical model. Hepatology 33, 419-423, (2001).

919 Ansaldi, F., Torre, F. & Bruzzone…, B. Evaluation of a new hepatitis C virus sequencing assay as a routine method for genotyping. J Med Virol, (2001).

920 Alvarez-Muñoz, M. T. et al. Hepatitis C virus RNA (HCV-RNA) in blood donors and family members seropositive for anti-HCV antibodies. Arch Med Res 32, 442-445, (2001).

921 Adinolfi, L., Utili, R., Andreana, A. & Tripodi…, M. Serum HCV RNA levels correlate with histological liver damage and concur with steatosis in progression of chronic hepatitis C. Digestive diseases and … 46, 1683, (2001).

922 Zucca, E. et al. Prevalence of Helicobacter pylori and hepatitis C virus infections among non-Hodgkin's lymphoma patients in Southern Switzerland. Haematologica 85, 147-153, (2000).

923 Yao, G.-B. et al. A multicenter, randomized, controlled trial of interferon alfacon-1 compared with α-2a-interferon in Chinese patients with chronic hepatitis C virus infection. Journal of Gastroenterology and Hepatology 15, 1165-1170, (2000).

924 Trimoulet, P. et al. TT virus infection during chronic hepatitis C. Am J Gastroenterol 95, 1765-1769, (2000).

925 Tanaka, H. et al. Effect of interferon therapy on the incidence of hepatocellular carcinoma and mortality of patients with chronic hepatitis C: a retrospective cohort study of 738 patients. Int J Cancer 87, 741-749, (2000).

926 Silva, L. K. et al. Hepatitis C virus genotypes in a northeastern area of Brazil. Am J Trop Med Hyg 62, 257-260, (2000).

927 Saracco, G., Sostegni, R. & Ghisetti…, V. Hepatitis C virus genotypes in a non-cirrhotic Italian population with chronic hepatitis C: correlation with clinical, virological and histological parameters. Results of a …. J Viral Hepat 7, 124-129, (2000).

928 Sanchez, J. L. et al. Hepatitis C in Peru: risk factors for infection, potential iatrogenic transmission, and genotype distribution. Am J Trop Med Hyg 63, 242-248, (2000).

929 Samokhvalov, E., Hijikata, M., Gylka, R., Lvov, D. & Mishiro, S. Full-genome nucleotide sequence of a hepatitis C virus variant (isolate name VAT96) representing a new subtype within the genotype 2 (arbitrarily 2k). Virus Genes 20, 183-187, (2000).

930 Rubbia-Brandt, L. et al. Hepatocyte steatosis is a cytopathic effect of hepatitis C virus genotype 3. J Hepatol 33, 106-115, (2000).

931 Ross, R., Viazov, S., Renzing-Köhler, K. & Roggendorf, M. Changes in the epidemiology of hepatitis C infection in Germany: Shift in the predominance of hepatitis C subtypes. J. Med. Virol. 60, 122-125, (2000).

932 Ross, R. et al. Genotyping of hepatitis C virus isolates using CLIP sequencing. J Clin Microbiol 38, 3581-3584, (2000).

933 Ray, S., Arthur, R., Carella, A., Bukh, J. & Thomas, D. Genetic Epidemiology of Hepatitis C Virus throughout Egypt. Journal of Infectious Diseases 182, 698-707, (2000).

934 Ramalho, F. et al. Correlation of genotypes and route of transmission with histologic activity and disease stage in chronic hepatitis C. Dig Dis Sci 45, 182-187, (2000).

935 Quarleri, J. F. et al. Genomic and phylogenetic analysis of hepatitis C virus isolates from argentine patients: a six-year retrospective study. J Clin Microbiol 38, 4560-4568, (2000).

936 Poupon, R. et al. Randomized trial of interferon-alpha plus ursodeoxycholic acid versus interferon plus placebo in patients with chronic hepatitis C resistant to interferon. Scand J Gastroenterol 35, 642-649, (2000).

937 Paraná, R. et al. HCV infection in northeastern Brazil: unexpected high prevalence of genotype 3a and absence of African genotypes. Arq Gastroenterol 37, 213-216, (2000).

938 Neumann, A. U. et al. Differences in viral dynamics between genotypes 1 and 2 of hepatitis C virus. J Infect Dis 182, 28-35, (2000).

939 Matsumura et al. Natural course of progression of liver fibrosis in Japanese patients with chronic liver disease type C – a study of 527 patients at one establishment. J Viral Hepat 7, 268-275, (2000).

940 Martinelli, A. L. et al. Porphyria cutanea tarda in Brazilian patients: association with hemochromatosis C282Y mutation and hepatitis C virus infection. Am J Gastroenterol 95, 3516-3521, (2000).

941 Maio, G. et al. Hepatitis C virus infection and alanine transaminase levels in the general population: a survey in a southern Italian town. J Hepatol 33, 116-120, (2000).

942 Lemaire, J. M. et al. HCV RNA in blood donors with isolated reactivities by third-generation RIBA. Transfusion 40, 867-870, (2000).

943 Krarup, H. et al. Haemophilic patients with hepatitis C have higher viral load compared to other well-defined patient groups. J Viral Hepat 7, 435-439, (2000).

944 Kazemi-Shirazi, L., Petermann, D. & Müller, C. Hepatitis B virus \DNA\ in sera and liver tissue of \HBsAg\ negative patients with chronic hepatitis C. J Hepatol 33, 785 - 790, (2000).

945 Kamal, S. et al. Clinical, virological and histopathological features: long-term follow-up in patients with chronic hepatitis C co-infected with S. mansoni. Liver 20, 281-289, (2000).

946 Inoue, Y. et al. Genotypic analysis of hepatitis C virus in blood donors in Indonesia. Am J Trop Med Hyg 62, 92-98, (2000).

947 Inoue, G., Horiike, N., Michitaka, K. & Onji, M. Hepatitis C virus clearance is prominent in women in an endemic area. Journal of Gastroenterology and Hepatology 15, 1054-1058, (2000).

948 Hsieh, M. et al. Virologic factors related to interferon-alpha-induced thyroid dysfunction in patients with chronic hepatitis C. European Journal of Endocrinology 142, 431-437, (2000).

949 Hoffmann, G. et al. Prevalence and clinical spectrum of chronic viral hepatitis in a middle-aged Swedish general urban population. Scand J Gastroenterol 35, 861-865, (2000).

950 Halfon, P. et al. Integrity of the NS5A (amino acid 2209 to 2248) region in hepatitis C virus 1b patients non-responsive to interferon therapy. Liver 20, 381-386, (2000).

951 Greub, G. et al. Clinical progression, survival, and immune recovery during antiretroviral therapy in patients with HIV-1 and hepatitis C virus coinfection: the Swiss HIV Cohort Study. Lancet 356, 1800-1805, (2000).

952 Gombas, W. et al. Prevalence and Distribution of Hepatitis C Subtypes in Patients with Opioid Dependence. Eur Addict Res 6, 198-204, (2000).

953 Fernandez, J. L. et al. Hepatitis G virus infection in hemodialysis patients and its relationship with hepatitis C virus infection. Am J Nephrol 20, 380-384, (2000).

954 Elghouzzi, M. H. et al. Hepatitis C Virus: Routes of Infection and Genotypes in a Cohort of Anti-HCV-Positive French Blood Donors. Vox Sang 79, 138-144, (2000).

955 Ebeling, F. et al. Leukocyte interferon-alpha in the treatment of chronic hepatitis C in Finland. Scand J Gastroenterol 35, 540-545, (2000).

956 Coppola, R., Masia, G., Pradat, P. & Trepo…, C. Impact of hepatitis C virus infection on healthy subjects on an Italian island. J Viral Hepat 7, 130-137, (2000).

957 Conte, D., Fraquelli, M., Prati, D., Colucci, A. & Minola, E. Prevalence and clinical course of chronic hepatitis C virus (HCV) infection and rate of HCV vertical transmission in a cohort of 15,250 pregnant women. Hepatology 31, 751-755, (2000).

958 Cicardi, M. et al. Prevalence and risk factors for the presence of serum cryoglobulins in patients with chronic hepatitis C. J Viral Hepat 7, 138-143, (2000).

959 Cavalletto, L., Chemello, L., Donada, C. & Casarin…, P. The pattern of response to interferon alpha (α-IFN) predicts sustained response to a 6-month α-IFN and ribavirin retreatment for chronic hepatitis C. J Hepatol 33, 128-134, (2000).

960 Causse, X., Payen, J., Izopet, J., Babany, G. & Girardin, M. Does HIV-infection influence the response of chronic hepatitis C to interferon treatment? A French multicenter prospective study. French Multicenter Study Group. J Hepatol 32, 1003-1010, (2000).

961 Castro, F. J. et al. Utility of early testing for HCV viremia as predictive factor of sustained response during interferon or interferon plus ribavirin treatment. J Hepatol 32, 843-849, (2000).

962 Calabrese, F., Pontisso, P. & Pettenazzo…, E. Liver cell apoptosis in chronic hepatitis C correlates with histological but not biochemical activity or serum HCV-RNA levels. Hepatology 31, 1153-1159, (2000).

963 Blatt, L. M. et al. Assessment of hepatitis C virus RNA and genotype from 6807 patients with chronic hepatitis C in the United States. J Viral Hepat 7, 196-202, (2000).

964 Bizollon, T. et al. Anti-hepatitis C virus core IgM antibodies correlate with hepatitis C recurrence and its severity in liver transplant patients. Gut 47, 698-702, (2000).

965 Berg, T. et al. Efficacy of a short-term ribavirin plus interferon alpha combination therapy followed by interferon alpha alone in previously untreated patients with chronic hepatitis C: a randomized multicenter trial. Liver 20, 427-436, (2000).

966 Belli, L., Zavaglia, C., Alberti, A. & Poli…, F. Influence of immunogenetic background on the outcome of recurrent hepatitis C after liver transplantation. Hepatology 31, 1345-1350, (2000).

967 Barbaro, G., Lorenzo, G., Soldini, M. & Giancaspro…, G. Evaluation of long-term efficacy of interferon alpha-2b and ribavirin in combination in naive patients with chronic hepatitis C: an Italian multicenter experience. J Hepatol 33, 448-455, (2000).

968 Balogun, M. A. et al. The prevalence and genetic diversity of hepatitis C infection in antenatal clinic attenders in two regions of England. Epidemiol Infect 125, 705-712, (2000).

969 Azzari, C., Resti, M., Moriondo, M. & Ferrari…, R. Vertical transmission of HCV is related to maternal peripheral blood mononuclear cell infection. Blood 96, 2045-2048, (2000).

970 Al-Faleh et al. Treatment of chronic hepatitis C genotype IV with interferon–ribavirin combination in Saudi Arabia: a multicentre study. J Viral Hepat 7, 287-291, (2000).

971 Yu, M. et al. Clinical application of the Quantiplex HCV RNA 2.0 and Amplicor HCV Monitor assays for quantifying serum hepatitis C virus RNA. J Clin Pathol 52, 807-811, (1999).

972 Yates, S. et al. Hepatocellular carcinoma in Egyptians with and without a history of hepatitis B virus infection: association with hepatitis C virus (HCV) infection but not with (HCV) RNA level. Am J Trop Med Hyg 60, 714-720, (1999).

973 Westin, J., Lindh, M., Lagging, L. M., Norkrans, G. & Wejstål, R. Chronic hepatitis C in Sweden: genotype distribution over time in different epidemiological settings. Scand J Infect Dis 31, 355-358, (1999).

974 Weiland, O. et al. Influence of pre-treatment factors on outcome of interferon-alpha treatment of patients with chronic hepatitis C. Scand J Infect Dis 31, 115-118, (1999).

975 Vogel, W. Treatment of acute hepatitis C virus infection. J Hepatol 31, Supplement 1, 189 - 192, (1999).

976 Villano, S. A., Vlahov, D., Nelson, K. E., Cohn, S. & Thomas, D. L. Persistence of viremia and the importance of long-term follow-up after acute hepatitis C infection. Hepatology 29, 908-914, (1999).

977 Vardas, E., Sitas, F., Seidel, K., Casteling, A. & Sim, J. Prevalence of hepatitis C virus antibodies and genotypes in asymptomatic, first-time blood donors in Namibia. Bull World Health Organ 77, 965-972, (1999).

978 van Rossum, T. G. et al. Intravenous glycyrrhizin for the treatment of chronic hepatitis C: a double-blind, randomized, placebo-controlled phase I/II trial. Journal of Gastroenterology and Hepatology 14, 1093-1099, (1999).

979 Smith, D. B. et al. A Second Outbreak of Hepatitis C Virus Infection from Anti–D Immunoglobulin in Ireland. Vox Sang 76, 175-180, (1999).

980 Shobokshi, O., Serebour, F., Skakni, L., Al-Saffy, Y. & Ahdal, M. Hepatitis C genotypes and subtypes in Saudi Arabia. J. Med. Virol. 58, 44-48, (1999).

981 Pontisso, P., Bellati, G., Brunetto, M. & Chemello…, L. Hepatitis C virus RNA profiles in chronically infected individuals: do they relate to disease activity? Hepatology 29, 585-589, (1999).

982 Pol, S. et al. A randomized trial of ribavirin and interferon-alpha vs. interferon-alpha alone in patients with chronic hepatitis C who were non-responders to a previous treatment. Multicenter Study Group under the coordination of the Necker Hospital, Paris, France. J Hepatol 31, 1-7, (1999).

983 Pérez García, F. J. et al. [Renal cell carcinoma. Clinical onset with peritoneal carcinomatosis. Report of a case]. Actas Urol Esp 23, 156-159, (1999).

984 Pawlotsky, J.-M. et al. Quantification of hepatitis C virus RNA in serum by branched DNA-based signal amplification assays. J Virol Methods 79, 227-235, (1999).

985 Osella, A., Sonzogni, L., Cavallini, A. & Foti…, L. Molecular epidemiology of hepatitis C virus infection in an area of hyperendemicity in southern Italy: a population-based study. J Clin Microbiol 37, 2371-2372, (1999).

986 Oliveira, M. L. et al. Prevalence and risk factors for HBV, HCV and HDV infections among injecting drug users from Rio de Janeiro, Brazil. Braz J Med Biol Res 32, 1107-1114, (1999).

987 Oliveira, M. L. et al. Distribution of HCV genotypes among different exposure categories in Brazil. Braz J Med Biol Res 32, 279-282, (1999).

988 Oliveira, G. C. et al. Hepatitis C virus genotypes in hemophiliacs in the state of Minas Gerais, Brazil. Transfusion 39, 1194-1199, (1999).

989 Neumayr, G., Propst, A., Schwaighofer, H., Judmaier, G. & Vogel, W. Lack of evidence for the heterosexual transmission of hepatitis C. QJM 92, 505-508, (1999).

990 Mihm, S. et al. Ratio of serum gamma-GT/ALT rather than ISDR variability is predictive for initial virological response to IFN-alpha in chronic HCV infection. J Med Virol 58, 227-234, (1999).

991 Menendez, C. et al. Prevalence and mother-to-infant transmission of hepatitis viruses B, C, and E in Southern Tanzania. J. Med. Virol. 58, 215-220, (1999).

992 Mathai, J. et al. IFN-alpha receptor mRNA expression in a United States sample with predominantly genotype 1a/I chronic hepatitis C liver biopsies correlates with response to IFN therapy. J Interferon Cytokine Res 19, 1011-1018, (1999).

993 Martinot-Peignoux, M. et al. Hepatitis C virus genotypes in France: relationship with epidemiology, pathogenicity and response to interferon therapy. J Viral Hepat 6, 435-443, (1999).

994 Maggi, G., Armitano, S., Brambilla, L., Brenna, M. & Cairo…, M. Hepatitis C infection in an Italian population not selected for risk factors. Liver 19, 427-431, (1999).

995 Lunel, F. et al. Comparative evaluation of hepatitis C virus RNA quantitation by branched DNA, NASBA, and monitor assays. Hepatology 29, 528-535, (1999).

996 Katsoulidou, A. et al. Molecular epidemiology of a hepatitis C virus outbreak in a haemodialysis unit. Multicentre Haemodialysis Cohort Study on Viral Hepatitis. Nephrology Dialysis Transplantation 14, 1188-1194, (1999).

997 Hagiwara, H. et al. Influence of transfusion-transmitted virus infection on the clinical features and response to interferon therapy in Japanese patients with chronic hepatitis C. J Viral Hepat 6, 463-469, (1999).

998 Grønbæk, K. et al. Natural history and etiology of liver disease in patients with previous community-acquired acute non-A, non-B hepatitis: A follow-up study of 178 Danish patients consecutively enrolled in The Copenhagen Hepatitis Acuta Programme in the period 1969–1987. J Hepatol 31, 800-807, (1999).

999 Gish, R. G. et al. Characterization of anti-hepatitis C virus-positive sera not genotyped by restriction fragment length polymorphism or serology. Journal of Gastroenterology and Hepatology 14, 339-344, (1999).

1000 Giannini, E., Ceppa, P., Botta, F., Fasoli, A. & Romagnoli…, P. Steatosis and bile duct damage in chronic hepatitis C: distribution and relationships in a group of Northern Italian patients. Liver 19, 432-437, (1999).

1001 Giannini, C., Giannelli, F., Monti, M. & Careccia…, G. Prevalence of mixed infection by different hepatitis C virus genotypes in patients with hepatitis C virus—related chronic liver disease. J Lab Clin Med 134, 68-73, (1999).

1002 Fakeeh, M. & Zaki, A. Hepatitis C: prevalence and common genotypes among ethnic groups in Jeddah, Saudi Arabia. Am J Trop Med Hyg 61, 889-892, (1999).

1003 Duvoux, C. et al. Low HCV replication levels in end-stage hepatitis C virus-related liver disease. J Hepatol 31, 593-597, (1999).

1004 Dickson, R. C., Mizokami, M., Orito, E., Qian, K. P. & Lau, J. Y. Quantification of serum HCV core antigen by a fluorescent enzyme immunoassay in liver transplant recipients with recurrent hepatitis C--clinical and virologic implications. Transplantation 68, 1512-1516, (1999).

1005 Dentico, P., Curatolo, N., Sacco, R. & De Luca, M. Hepatitis C virus serotypes and sources of infection in patients with HCV-related chronic liver disease from one geographical area in southeast Italy. Infection 27, 118-121, (1999).

1006 Cresta, P. et al. Response to interferon alpha treatment and disappearance of cryoglobulinaemia in patients infected by hepatitis C virus. Gut 45, 122-128, (1999).

1007 Colina, R., Azambuja, C., Uriarte, R., Mogdasy, C. & Cristina, J. Evidence of increasing diversification of hepatitis C viruses. J Gen Virol 80 (Pt 6), 1377-1382, (1999).

1008 Christensen, P. et al. Transfusion-acquired hepatitis C: the Danish lookback experience. Transfusion 39, 188-193, (1999).

1009 Chaudhary, R., Tepper, M., Eisaadany, S. & Gully, P. R. Distribution of hepatitis C virus genotypes in Canada: Results from the LCDC Sentinel Health Unit Surveillance System. Can J Infect Dis 10, 53-56, (1999).

1010 Cacciola, I., Pollicino, T. & Squadrito…, G. Occult hepatitis B virus infection in patients with chronic hepatitis C liver disease. NEJM 341, 22-26, (1999).

1011 Buoro, S., Pizzighella, S., Boschetto, R. & Pellizzari…, L. Typing of hepatitis C virus by a new method based on restriction fragment length polymorphism. Intervirology 42, 1-8, (1999).

1012 Brojer, E. et al. Anti-HCV RIBA/LiaTek reactivity and HCV genotype in EIA-negative patients with viremia. J. Med. Virol. 59, 451-455, (1999).

1013 Bellentani, S., Pozzato, G., Saccoccio, G. & Crovatto…, M. Clinical course and risk factors of hepatitis C virus related liver disease in the general population: report from the Dionysos study. Gut 44, 874-880, (1999).

1014 Barbaro, G. et al. Hepatocellular mitochondrial alterations in patients with chronic hepatitis C: ultrastructural and biochemical findings. Am J Gastroenterol 94, 2198-2205, (1999).

1015 Asti, M., Martinetti, M., Zavaglia, C. & Cuccia…, M. Human leukocyte antigen class II and III alleles and severity of hepatitis C virus–related chronic liver disease. Hepatology 29, 1272-1279, (1999).

1016 Alter, M. J. et al. The prevalence of hepatitis C virus infection in the United States, 1988 through 1994. N Engl J Med 341, 556-562, (1999).

1017 Alric, L. et al. Study of the association between major histocompatibility complex class II genes and the response to interferon alpha in patients with chronic hepatitis C infection. Hum Immunol 60, 516-523, (1999).

1018 Zeuzem, S. et al. Quantification of the initial decline of serum hepatitis C virus RNA and response to interferon alfa. Hepatology 27, 1149-1156, (1998).

1019 Zavaglia, C. et al. Association between HLA class II alleles and protection from or susceptibility to chronic hepatitis C. J Hepatol 28, 1-7, (1998).

1020 Wong, D., Tong, L. & Lim, W. High prevalence of hepatitis C virus genotype 6 among certain risk groups in Hong Kong. European Journal of Epidemiology 14, 421-426, (1998).

1021 Vince, A. et al. HCV genotypes in patients with chronic hepatitis C in Croatia. Infection 26, 173-177, (1998).

1022 Verbaan, H., Widell, A., Bondeson, L., Andersson, K. & Eriksson, S. Factors associated with cirrhosis development in chronic hepatitis C patients from an area of low prevalence. J Viral Hepat 5, 43-51, (1998).

1023 Valtuille, R. et al. Evidence of hepatitis C virus passage across dialysis membrane. Nephron 80, 194-196, (1998).

1024 Toro, F., Conesa, A., Garcia, A., Bianco, N. E. & De Sanctis, J. B. Increased peroxide production by polymorphonuclear cells of chronic hepatitis C virus-infected patients. Clin Immunol Immunopathol 88, 169-175, (1998).

1025 Thiers, V. et al. Hepatitis G virus infection in hepatitis C virus-positive patients co-infected or not with hepatitis B virus and/or human immunodeficiency virus. J Viral Hepat 5, 123-130, (1998).

1026 Sreevatsan, S. et al. Algorithmic approach to high-throughput molecular screening for alpha interferon-resistant genotypes in hepatitis C patients. J Clin Microbiol 36, 1895-1901, (1998).

1027 Sim, H., Yim, C., Krajden, M. & Heathcote, J. Durability of serological remission in chronic hepatitis C treated with interferon-alpha-2B. Am J Gastroenterol 93, 39-43, (1998).

1028 Shemer-Avni, Y. et al. Hepatitis C virus infection and genotypes in Southern Israel and the Gaza strip. J. Med. Virol. 56, 230-233, (1998).

1029 Shapiro et al. mRNA cytokine profile in peripheral blood cells from chronic hepatitis C virus (HCV)-infected patients: effects of interferon-alpha (IFN-α) treatment. Clinical & Experimental Immunology 114, 55-60, (1998).

1030 Serfaty, L. et al. Determinants of outcome of compensated hepatitis C virus-related cirrhosis. Hepatology 27, 1435-1440, (1998).

1031 Schneeberger, P. M. et al. Hepatitis C virus infections in dialysis centers in The Netherlands: a national survey by serological and molecular methods. J Clin Microbiol 36, 1711-1715, (1998).

1032 Rostaing, L. et al. IMPACT OF HEPATITIS C VIRUS DURATION AND HEPATITIS C VIRUS GENOTYPES ON RENAL TRANSPLANT PATIENTS: Correlation with Clinicopathological Features. Transplantation KW - 65, (1998).

1033 Roffi, L., Ricci, A., Ogliari, C., Scalori, A. & Minola…, E. HCV genotypes in Northern Italy: a survey of 1368 histologically proven chronic hepatitis C patients. J Hepatol 29, 701-706, (1998).

1034 Rodrigues, A. et al. Hepatitis C virus genotypes and the influence of the induction of immunosuppression with anti-thymocyte globulin (ATG) on chronic hepatitis in renal graft recipients. Transplant International 11 Suppl 1, S115-118, (1998).

1035 Reichard, O. et al. Randomised, double-blind, placebo-controlled trial of interferon alpha-2b with and without ribavirin for chronic hepatitis C. The Swedish Study Group. Lancet 351, 83-87, (1998).

1036 Pramoolsinsap, C. et al. Hepatitis G infection and therapeutic response to interferon in HCV -related chronic liver disease. Southeast Asian J Trop Med Public Health 29, 480-491, (1998).

1037 Pol, S. et al. Retrospective analysis of the impact of HIV infection and alcohol use on chronic hepatitis C in a large cohort of drug users. J Hepatol 28, 945-950, (1998).

1038 Pirisi, M. et al. Factors associated with serum HCV RNA positivity in anti-HCV antibody positive intravenous drug users. J Clin Epidemiol 51, 423-427, (1998).

1039 Payen, J. et al. Better efficacy of a 12-month interferon alfa-2b retreatment in patients with chronic hepatitis C relapsing after a 6-month treatment: a multicenter, controlled, randomized trial. Le Groupe D'etude et De Traitement du Virus De L'hepatite C (Get.Vhc). Hepatology 28, 1680-1686, (1998).

1040 Ostapowicz, G., Watson, K., Locarnini, S. & Desmond, P. Role of alcohol in the progression of liver disease caused by hepatitis C virus infection. Hepatology 27, 1730-1735, (1998).

1041 Niederau, C. et al. Prognosis of chronic hepatitis C: results of a large, prospective cohort study. Hepatology 28, 1687-1695, (1998).

1042 Natov, S. N. et al. Serologic and virologic profiles of hepatitis C infection in renal transplant candidates. New England Organ Bank Hepatitis C Study Group. Am J Kidney Dis 31, 920-927, (1998).

1043 Monica, F. et al. Hepatitis C virus infection and related chronic liver disease in a resident elderly population: the Silea Study. J Viral Hepat 5, 345-351, (1998).

1044 McHutchison, J. G. et al. Interferon alfa-2b alone or in combination with ribavirin as initial treatment for chronic hepatitis C. Hepatitis Interventional Therapy Group. N Engl J Med 339, 1485-1492, (1998).

1045 Lumbreras, C. et al. Clinical, virological, and histologic evolution of hepatitis C virus infection in liver transplant recipients. Clin Infect Dis 26, 48-55, (1998).

1046 Leroy, V. et al. Role of anti-interferon antibodies in breakthrough occurrence during alpha 2a and 2b therapy in patients with chronic hepatitis C. J Hepatol 28, 375-381, (1998).

1047 Leone, F. et al. Hepatitis C virus (HCV) hypervariable region 1 complexity does not correlate with severity of liver disease, HCV type, viral load or duration of infection. J Hepatol 29, 689-694, (1998).

1048 Le Pogam, S. et al. Comparison of DNA enzyme immunoassay and line probe assays (Inno-LiPA HCV I and II) for hepatitis C virus genotyping. J Clin Microbiol 36, 1461-1463, (1998).

1049 Lampe, E. et al. Hepatitis G virus (GBV-C) infection among Brazilian patients with chronic liver disease and blood donors. Clin Diagn Virol 9, 1-7, (1998).

1050 Lamoril, J. et al. Epidemiology of hepatitis C and G in sporadic and familial porphyria cutanea tarda. Hepatology 27, 848-852, (1998).

1051 Kumar, R. M. & Shahul, S. Role of breast-feeding in transmission of hepatitis C virus to infants of HCV-infected mothers. J Hepatol 29, 191 - 197, (1998).

1052 Knolle, P. et al. Viral and host factors in the prediction of response to interferon-alpha therapy in chronic hepatitis C after long-term follow-up. J Viral Hepat 5, 399-406, (1998).

1053 Kleter, B. et al. Hepatitis C virus genotypes: epidemiological and clinical associations. Benelux Study Group on Treatment of Chronic Hepatitis C. Liver 18, 32-38, (1998).

1054 Khorsi, H. et al. Amplification and detection of the terminal 3' non-coding region of hepatitis C virus isolates. Res Virol 149, 115-121, (1998).

1055 Kasahara, A. et al. Risk factors for hepatocellular carcinoma and its incidence after interferon treatment in patients with chronic hepatitis C. Osaka Liver Disease Study Group. Hepatology 27, 1394-1402, (1998).

1056 Kakumu, S. et al. Prevalence of hepatitis B, hepatitis C, and GB virus C/hepatitis G virus infections in liver disease patients and inhabitants in Ho Chi Minh, Vietnam. J Med Virol 54, 243-248, (1998).

1057 Kaba, S. et al. Molecular epidemiology of hepatitis C in Australia. J Gastroenterol Hepatol 13, 914-920, (1998).

1058 Jeannel, D. et al. Evidence for high genetic diversity and long-term endemicity of hepatitis C virus genotypes 1 and 2 in West Africa. J. Med. Virol. 55, 92-97, (1998).

1059 Jadoul, M., Cornu, C. & van Ypersele de Strihou, C. Universal precautions prevent hepatitis C virus transmission: a 54 month follow-up of the Belgian Multicenter Study. The Universitaires Cliniques St-Luc (UCL) Collaborative Group. Kidney Int 53, 1022-1025, (1998).

1060 Izopet, J. et al. Baseline level and early suppression of serum HCV RNA for predicting sustained complete response to alpha-interferon therapy. J Med Virol 54, 86-91, (1998).

1061 Hizel, N., Tuncbilek, S., Boyacıogˇlu, S., Ozdemir, N. & Haberal, M. Hepatitis G Virus Infection in Haemodialysis Patients and its Relationship with Hepatitis C Virus RNA Positivity. Scand J Infect Dis 30, 451-454, (1998).

1062 Hayashi, J. et al. Age-Related Response to Interferon Alfa Treatment in Women vs Men With Chronic Hepatitis C Virus Infection. Arch Intern Med 158, 177-181, (1998).

1063 Frangeul, L. et al. Mutations in NS5A region of hepatitis C virus genome correlate with presence of NS5A antibodies and response to interferon therapy for most common European hepatitis C virus genotypes. Hepatology 28, 1674-1679, (1998).

1064 Doglio, A., Laffont, C., Thyss, S. & Lefebvre, J. Rapid genotyping of hepatitis C virus by direct cycle sequencing of PCR-amplified cDNAs and capillary electrophoresis analysis. Res Virol 149, 219-227, (1998).

1065 Cammà, C., Di Marco, V., Lo Iacono, O. & Almasio…, P. Long-term course of interferon-treated chronic hepatitis C. J Hepatol 28, 531-537, (1998).

1066 Burjel, L. et al. Characterization of hepatitis C virus genotypes in an hemodialysis unit in Paysandú, Uruguay. Rev Argent Microbiol 30, 190-194, (1998).

1067 Andreone, P., Zignego, A. & Cursaro…, C. Prevalence of monoclonal gammopathies in patients with hepatitis C virus infection. Ann Intern Med 129, 294-298, (1998).

1068 Agha, S., Shérif, L., Allam, M. & Fawzy, M. Transplacental transmission of hepatitis C virus in HIV-negative mother. Res Virol 149, 229 - 234, (1998).

1069 Abdulkarim, A. et al. Hepatitis C virus genotypes and hepatitis G virus in hemodialysis patients from Syria: identification of two novel hepatitis C virus subtypes. Am J Trop Med Hyg 59, 571-576, (1998).

1070 Viazov, S. et al. Hepatitis C virus genotypes in different regions of the former Soviet Union (Russia, Belarus, Moldova, and Uzbekistan). J. Med. Virol. 53, 36-40, (1997).

1071 Thiers, V., Jaffredo, F., Tuveri, R., Chodan, N. & Bréchot, C. Development of a simple restriction fragment length polymorphism (RFLP) based assay for HCV genotyping and comparative analysis with genotyping and serotyping tests. J Virol Methods 65, 9-17, (1997).

1072 Tanzi, M. L. et al. Antipoliomyelitis neutralizing antibodies in maternal and neonatal serum. European Journal of Epidemiology 13, 559-565, (1997).

1073 Silvestri, F., Barillari, G., Fanin, R., Pipan, C. & Falasca…, E. The genotype of the hepatitis C virus in patients with HCV-related B cell non-Hodgkin's lymphoma. Leukemia 11, 2215-2234, (1997).

1074 Shrestha, S. et al. Infection with GB virus C and hepatitis C virus in drug addicts, patients on maintenance hemodialysis, or with chronic liver disease in Nepal. J Med Virol 53, 157-161, (1997).

1075 Shiratori, Y. et al. Predictors of the efficacy of interferon therapy in chronic hepatitis C virus infection. Tokyo-Chiba Hepatitis Research Group. Gastroenterology 113, 558-566, (1997).

1076 Serfaty, L. et al. Risk factors for cirrhosis in patients with chronic hepatitis C virus infection: Results of a case-control study. Hepatology 26, 776-779, (1997).

1077 Seme, K. et al. Molecular evidence for nosocomial spread of two different hepatitis C virus strains in one hemodialysis unit. Nephron 77, 273-278, (1997).

1078 Seme, K. et al. Distribution of hepatitis C virus genotypes in Slovenia. Scand J Infect Dis 29, 29-31, (1997).

1079 Saracco, G., Borghesio, E., Mesina, P. & Solinas…, A. Prolonged treatment (2 years) with different doses (3 versus 6 MU) of interferon α-2b for chronic hepatitis type C: Results of a multicenter randomized trial. J Hepatol 27, 56-62, (1997).

1080 Rumi, M., Santagostino, E. & Morfini…, M. A Multicenter Controlled, Randomized, Open Trial of Interferon α2b Treatment of Anti–Human Immunodeficiency Virus-Negative Hemophilic Patients With Chronic …. Blood 89, 3529-3533, (1997).

1081 Poynard, T., Bedossa, P. & Opolon, P. Natural history of liver fibrosis progression in patients with chronic hepatitis C. The OBSVIRC, METAVIR, CLINIVIR, and DOSVIRC groups. Lancet 349, 825-832, (1997).

1082 Picchio, G. R. et al. Hepatitis C (HCV) genotype and viral titer distribution among Argentinean hemophilic patients in the presence or absence of human immunodeficiency virus (HIV) co-infection. J. Med. Virol. 52, 219-225, (1997).

1083 Pawlotsky, J. et al. Serological determination of hepatitis C virus genotype: comparison with a standardized genotyping assay. J Clin Microbiol 35, 1734-1739, (1997).

1084 Papatheodoridis, G. et al. Significance of IgM anti-HCVcore level in chronic hepatitis C. J Hepatol 27, 36-41, (1997).

1085 Ohno, O. et al. New hepatitis C virus (HCV) genotyping system that allows for identification of HCV genotypes 1a, 1b, 2a, 2b, 3a, 3b, 4, 5a, and 6a. J Clin Microbiol 35, 201-207, (1997).

1086 Noguchi, S., Sata, M., Suzuki, H., Mizokami, M. & Tanikawa, K. Routes of Transimission of Hepatitis C Virus in an Endemic Rural Area of Japan : Molecular Epidemiologic Study of Hepatitis C Virus Infection. Scand J Infect Dis 29, 23-28, (1997).

1087 Monteverde, A., Ballarè, M. & Pileri, S. Hepatic lymphoid aggregates in chronic hepatitis C and mixed cryoglobulinemia. Springer seminars in immunopathology 19, 99-110, (1997).

1088 Mison, L. et al. Prevalence of hepatitis C virus and genotype distribution in an Australian volunteer blood donor population. Transfusion 37, 73-78, (1997).

1089 Mendel, I. et al. Detection and genotyping of the hepatitis C RNA in tear fluid from patients with chronic hepatitis C. J. Med. Virol. 51, 231-233, (1997).

1090 McHutchison, J. G. et al. Hepatitis C and G co-infection: response to interferon therapy and quantitative changes in serum HGV-RNA. Hepatology 26, 1322-1327, (1997).

1091 McCaw, R., Moaven, L., Locarnini, S. & Bowden, D. Hepatitis C virus genotypes in Australia. J Viral Hepat 4, 351-357, (1997).

1092 Marshall, D. J. et al. Determination of hepatitis C virus genotypes in the United States by cleavase fragment length polymorphism analysis. J Clin Microbiol 35, 3156-3162, (1997).

1093 Marcellin, P. et al. Long-term histologic improvement and loss of detectable intrahepatic HCV RNA in patients with chronic hepatitis C and sustained response to interferon-alpha therapy. Ann Intern Med 127, 875-881, (1997).

1094 Manzin, A. et al. Quantitative analysis of hepatitis C virus activity in vivo in different groups of untreated patients. Arch Virol 142, 465-472, (1997).

1095 Mangia, A., Cascavilla, I., Lezzi, G. & Spirito…, F. HCV genotypes in patients with liver disease of different stages and severity. J Hepatol 26, 1173-1178, (1997).

1096 López-Labrador, F. X. et al. Hepatitis C virus (HCV) genotypes in Spanish patients with HCV infection: relationship between HCV genotype 1b, cirrhosis and hepatocellular carcinoma. J Hepatol 27, 959-965, (1997).

1097 Lee, J.-H., Roth, W. K. & Zeuzem, S. Evaluation and comparison of different hepatitis C virus genotyping and serotyping assays. J Hepatol 26, 1001-1009, (1997).

1098 Le Guen, B. et al. Hepatitis C virus genome complexity correlates with response to interferon therapy: a study in French patients with chronic hepatitis C. Hepatology 25, 1250-1254, (1997).

1099 KUWANA, K. et al. Risk factors and the effect of interferon therapy in the development of hepatocellular carcinoma: A multivariate analysis in 343 patients. Journal of Gastroenterology and Hepatology 12, 149-155, (1997).

1100 Imhof, M., Popal, H., Lee, J.-H., Zeuzem, S. & Milbradt, R. Prevalence of Hepatitis C Virus Antibodies and Evaluation of Hepatitis C Virus Genotypes in Patients with Lichen planus. Dermatology 195, 1-5, (1997).

1101 Haydon, G. H. et al. Association between chronic hepatitis C infection and hepatocellular carcinoma in a Scottish population. Gut 40, 128-132, (1997).

1102 Hassoba, H. et al. Antibody to GBV-C second envelope glycoprotein (anti-GBV-C E2): Is it a marker for immunity? J. Med. Virol. 53, 354-360, (1997).

1103 Guadagnino, V., Stroffolini, T. & Rapicetta…, M. Prevalence, risk factors, and genotype distribution of hepatitis C virus infection in the general population: a community-based survey in southern Italy. Hepatology 26, 1006-1011, (1997).

1104 Goeser, T. et al. Clinical presentation of GB-C virus infection in drug abusers with chronic hepatitis C. J Hepatol 26, 498-502, (1997).

1105 Ginabreda, M. G., Yoshida, C. F. & Niel, C. Genomic characterization of Brazilian hepatitis C virus genotypes 1a and 1b. Braz J Med Biol Res 30, 339-345, (1997).

1106 Gaeta, G. B. et al. Human leucocyte interferon-alpha in chronic hepatitis C resistant to recombinant or lymphoblastoid interferon-alpha: a randomized controlled trial. J Viral Hepat 4, 209-214, (1997).

1107 Francesconi, R., Giostra, F., Ballardini, G. & Manzin…, A. Clinical implications of GBV-C/HGV infection in patients with "HCV-related" chronic hepatitis. J Hepatol 26, 1165-1172, (1997).

1108 Forns, X. et al. Incidence and risk factors of hepatitis C virus infection in a haemodialysis unit. Nephrol Dial Transplant 12, 736-740, (1997).

1109 Feucht, H. et al. The influence of age on the prevalence of hepatitis C virus subtypes 1a and 1b. J Infect Dis 175, 685-688, (1997).

1110 Fernández, I. et al. Influence of viral genotype and level of viremia on the severity of liver injury and the response to interferon therapy in Spanish patients with chronic C infection. Scand J Gastroenterol 32, 70-76, (1997).

1111 Fabrizi, F., Lunghi, G., Andrulli, S. & Pagliari…, B. Influence of hepatitis C virus (HCV) viraemia upon serum aminotransferase activity in chronic dialysis patients. Nephrology Dialysis Transplantation 12, 1394-1398, (1997).

1112 Dubois, F., Desenclos, J., Mariotte, N. & Goudeau, A. Hepatitis C in a French population-based survey, 1994: seroprevalence, frequency of viremia, genotype distribution, and risk factors. The Collaborative Study Group. Hepatology 25, 1490-1496, (1997).

1113 Donato, F., Tagger, A., Chiesa, R. & Ribero…, M. Hepatitis B and C virus infection, alcohol drinking, and hepatocellular carcinoma: A case-control study in Italy. Hepatology 26, 579-584, (1997).

1114 Cribier, B. et al. HIV increases hepatitis C viraemia irrespective of the hepatitis C virus genotype. Res Virol 148, 267-271, (1997).

1115 Corado, J. et al. Impairment of natural killer (NK) cytotoxic activity in hepatitis C virus (HCV) infection. Clin Exp Immunol 109, 451-457, (1997).

1116 Cicciarello, S. et al. Prevalence of hepatitis C virus genotypes in southern Italy. European Journal of Epidemiology 13, 49-54, (1997).

1117 CHEN, J. et al. Hepatitis C virus genotypes in a cohort of Australian blood donors and haemophiliac and liver transplant patients. Journal of Gastroenterology and Hepatology 12, 182-187, (1997).

1118 Cesaro, S., Petris, M. & Rossetti…, F. Chronic hepatitis C virus infection after treatment for pediatric malignancy. Blood 90, 1315-1320, (1997).

1119 Castelain, S. et al. Comparison of hepatitis C virus serotyping and genotyping in French patients. Clin Diagn Virol 7, 159-165, (1997).

1120 Bruno, S., Silini, E., Crosignani, A. & Borzio…, F. Hepatitis C virus genotypes and risk of hepatocellular carcinoma in cirrhosis: a prospective study. Hepatology 25, 754-758, (1997).

1121 Böker, K. et al. Long-term outcome of hepatitis C virus infection after liver transplantation. Hepatology 25, 203-210, (1997).

1122 Biasin, M., Fiordalisi, G., Zanella, I. & Cavicchini…, A. A DNA hybridization method for typing hepatitis C virus genotype 2c. J Virol Methods 65, 307-315, (1997).

1123 Berg, T. et al. Distribution of hepatitis C virus genotypes in German patients with chronic hepatitis C: correlation with clinical and virological parameters. J Hepatol 26, 484-491, (1997).

1124 Benvegnu, L., Pontisso, P., Cavalletto, D. & Noventa…, F. Lack of correlation between hepatitis C virus genotypes and clinical course of hepatitis C virus-related cirrhosis. Hepatology 25, 211-215, (1997).

1125 Bell, H. et al. Genotype, viral load and age as independent predictors of treatment outcome of interferon-alpha 2a treatment in patients with chronic hepatitis C. Construct group. Scand J Infect Dis 29, 17-22, (1997).

1126 Zhou, S. et al. Severity of liver disease in liver transplantation recipients with hepatitis C virus infection: relationship to genotype and level of viremia. Hepatology 24, 1041-1046, (1996).

1127 Zein, N. N. et al. Hepatitis C virus genotypes in the United States: epidemiology, pathogenicity, and response to interferon therapy. Collaborative Study Group. Ann Intern Med 125, 634-639, (1996).

1128 Watson, J. P. et al. Hepatitis C virus: epidemiology and genotypes in the north east of England. Gut 38, 269-276, (1996).

1129 Tong, C. et al. The occurrence of hepatitis B and C viruses in Pakistani patients with chronic liver disease and hepatocellular carcinoma. Epidemiol Infect 117, 327-332, (1996).

1130 TAKADA, A. et al. Relationship between hepatocellular carcinoma and subtypes of hepatitis C virus: A nationwide analysis. Journal of Gastroenterology and Hepatology 11, 166-169, (1996).

1131 Stuyver, L. et al. Hepatitis C virus in a hemodialysis unit: molecular evidence for nosocomial transmission. Kidney Int 49, 889-895, (1996).

1132 Soetjipto et al. Differential prevalence of hepatitis C virus subtypes in healthy blood donors, patients on maintenance hemodialysis, and patients with hepatocellular carcinoma in Surabaya, Indonesia. J Clin Microbiol 34, 2875-2880, (1996).

1133 Rumi, M., Del Ninno, E., Parravicini, M. & Romeo…, R. A prospective, randomized trial comparing lymphoblastoid to recombinant interferon alfa 2a as therapy for chronic hepatitis C. Hepatology 24, 1366-1370, (1996).

1134 Ruggieri, A. et al. Heterogeneity of Hepatitis C Virus Genotype 2 Variants in West Central Africa (Guinea Conakry). Journal of General Virology 77, 2073-2076, (1996).

1135 Romeo, R., Colombo, M. & Rumi…, M. Lack of association between type of hepatitis C virus, serum load and severity of liver disease. J Viral Hepat 3, 183-190, (1996).

1136 Quiroga, J. A., Pardo, M., Navas, S., Martín, J. & Carreño, V. Patterns of immune responses to the host-encoded GOR and hepatitis C virus core-derived epitopes with relation to hepatitis C viremia, genotypes, and liver disease severity. J Infect Dis 173, 300-305, (1996).

1137 Prati, D. et al. Influence of different hepatitis C virus genotypes on the course of asymptomatic hepatitis C virus infection. Gastroenterology 110, 178-183, (1996).

1138 Pohjanpelto, P., Lappalainen, M., Widell, A., Asikainen, K. & Paunio, M. Hepatitis C genotypes in Finland determined by RFLP. Clin Diagn Virol 7, 7-16, (1996).

1139 Pawlotsky, J. et al. Factors affecting treatment responses to interferon-alpha in chronic hepatitis C. J Infect Dis 174, 1-7, (1996).

1140 Panigrahi, A., Roca, J., Acharya, S., Jameel, S. & Panda, S. Genotype determination of hepatitis C virus from Northern India: Identification of a new subtype. J. Med. Virol. 48, 191-198, (1996).

1141 Oni, A. & Harrison, T. Genotypes of hepatitis C virus in Nigeria. J. Med. Virol. 49, 178-186, (1996).

1142 Murphy, D. G. et al. Biological and clinicopathological features associated with hepatitis C virus type 5 infections. J Hepatol 24, 109-113, (1996).

1143 Lovo, D. K. et al. Prevalence of hepatitis C virus and distribution of its genotypes in Northern Eurasia. Arch Virol 141, 1613-1622, (1996).

1144 Löve, A. et al. Characteristics of hepatitis C virus among intravenous drug users in Iceland. Am J Epidemiol 143, 631-636, (1996).

1145 Jarvis, L. et al. Investigation of the relative infectivity and pathogenicity of different hepatitis C virus genotypes in hemophiliacs. Blood 87, 3007-3011, (1996).

1146 Ikeda, K. et al. Hepatitis C virus subtype 3b infection in a hospital in Japan: Epidemiological study. Journal of Gastroenterology 31, 801-805, (1996).

1147 Huber, K. R., Sebesta, C. & Bauer, K. Detection of common hepatitis C virus subtypes with a third-generation enzyme immunoassay. Hepatology 24, 471-473, (1996).

1148 Huber, K., Sebesta, C. & Bauer, K. Detection of common hepatitis C virus subtypes with a third-generation enzyme immunoassa. Hepatology 24, 471-473, (1996).

1149 Hatzakis, A. et al. Hepatitis C virus 1b is the dominant genotype in HCV-related carcinogenesis: a case-control study. Int J Cancer 68, 51-53, (1996).

1150 Halfon, P. et al. Myasthenia gravis and hepatitis C virus infection. J Viral Hepat 3, 329-332, (1996).

1151 González-Peralta, R. P. et al. Clinical implications of viral quasispecies heterogeneity in chronic hepatitis C. J. Med. Virol. 49, 242-247, (1996).

1152 Gane, E. J. et al. Long-term outcome of hepatitis C infection after liver transplantation. N Engl J Med 334, 815-820, (1996).

1153 Feucht, H. et al. Distribution of genotypes and response to alpha-interferon in patients with hepatitis C virus infection in Germany. Eur J Clin Microbiol Infect Dis 15, 128-132, (1996).

1154 Dhaliwal, S. K. et al. Influence of viraemia and genotype upon serological reactivity in screening assays for antibody to hepatitis C virus. J. Med. Virol. 48, 184-190, (1996).

1155 de Lamballerie, X., Olmer, M., Bouchouareb, D., Zandotti, C. & De Micco, P. Nosocomial transmission of hepatitis C virus in haemodialysis patients. J. Med. Virol. 49, 296-302, (1996).

1156 Colleoni, N., Bucci, R., Ribero, M. & Zhou…, J. Hepatitis C virus genotype in anti-HCV-positive hemodialysed patients. Nephrology Dialysis Transplantation 11, 2258-2264, (1996).

1157 Cilla, G. et al. Genotyping of hepatitis C virus isolates from Basque Country, Spain. Epidemiol Infect 117, 533-536, (1996).

1158 Bernier, L., Willems, B., Delage, G. & Murphy, D. G. Identification of numerous hepatitis C virus genotypes in Montreal, Canada. J Clin Microbiol 34, 2815-2818, (1996).

1159 Berger, A., von Depka Prondzinski, M., Doerr, H., Rabenau, H. & Weber, B. Hepatitis C plasma viral load is associated with HCV genotype but not with HIV coinfection. J Med Virol 48, 339-343, (1996).

1160 Berg, T. et al. Responsiveness to interferon alpha treatment in patients with chronic hepatitis C coinfected with hepatitis G virus. J Hepatol 25, 763-768, (1996).

1161 Andonov, A., Teoharov, P. & Bakalova, S. Predominance of hepatitis C virus genotype 1b in Bulgaria. Eur J Clin Microbiol Infect Dis 15, 521-523, (1996).

1162 Zeuzem, S., Rüster, B., Lee, J.-H., Stripf, T. & Roth, W. K. Evaluation of a reverse hybridization assay for genotyping of hepatitis C virus. J Hepatol 23, 654-661, (1995).

1163 Valliammai, T., Thyagarajan, S. P., Zuckerman, A. J. & Harrison, T. J. Diversity of genotypes of hepatitis C virus in southern India. Journal of General Viology 76, 711-716, (1995).

1164 Tawaraya, H. et al. Epidemiologic survey and genetic analysis of endemic hepatitis C virus infection in a Japanese town with a high prevalence of hepatitis B virus carriers. J. Med. Virol. 45, 367-372, (1995).

1165 Tanaka, E. et al. Epidemiology of genotypes of hepatitis C virus in Japanese patients with type C chronic liver diseases: A multi-institution analysis. Journal of Gastroenterology and Hepatology 10, 538-545, (1995).

1166 Stark, K. et al. Prevalence and Determinants of Anti-HCV Seropositivity and of HCV Genotype among Intravenous Drug Users in Berlin. Scand J Infect Dis 27, 331-337, (1995).

1167 Silini, E., Bono, F., Cividini, A., Cerino, A. & Bruno…, S. Differential distribution of hepatitis C virus genotypes in patients with and without liver function abnormalities. Hepatology 21, 285-290, (1995).

1168 Shev, S. et al. HCV genotypes in Swedish blood donors as correlated to epidemiology, liver disease and hepatitis C virus antibody profile. Infection 23, 253-257, (1995).

1169 Sheng, L., Willems, M., Peerlinck, K., Yap, S. & Vermylen, J. Hepatitis C virus genotypes in belgian hemophiliacs. J. Med. Virol. 45, 211-214, (1995).

1170 Power, J. P. et al. Molecular epidemiology of an outbreak of infection with hepatitis C virus in recipients of anti-D immunoglobulin. The Lancet 345, 1211-1213, (1995).

1171 Pontisso, P. et al. Distribution of three major hepatitis C virus genotypes in Italy. A multicentre study of 495 patients with chronic hepatitis C. J Viral Hepat 2, 33-38, (1995).

1172 Pol, S. et al. The changing relative prevalence of hepatitis C virus genotypes: Evidence in hemodialyzed patients and kidney recipients. Gastroenterology 108, 581-583, (1995).

1173 Pernas, M. et al. Sequence of non-structural regions 3 and 5 of hepatitis C virus genomes from Spanish patients: existence of a predominant variant related to type 1b. J Gen Virol 76 (Pt 2), 415-420, (1995).

1174 Pawlotsky, J. et al. Relationship between hepatitis C virus genotypes and sources of infection in patients with chronic hepatitis C. J Infect Dis 171, 1607-1610, (1995).

1175 Pawlotsky, J. et al. Influence of hepatitis C virus (HCV) genotypes on HCV recombinant immunoblot assay patterns. J Clin Microbiol 33, 1357-1359, (1995).

1176 Oubiña, J. R. et al. Genomic characterization of hepatitis C virus isolates from Argentina. J. Med. Virol. 47, 97-104, (1995).

1177 Ng, W. et al. Hepatitis C virus genotypes in Singapore and Indonesia. J Viral Hepat 2, 203-209, (1995).

1178 Mendel, I., Clotteau, L., Lambed, S. & Buffet-Janvresse, C. Hepatitis C virus infection in an HIV-positive population in normandy: Antibodies, HCV RNA and genotype prevalence. J. Med. Virol. 47, 231-236, (1995).

1179 Mellor, J., Holmes, E. C., Jarvis, L. M., Yap, P. L. & Simmonds, P. Investigation of the pattern of hepatitis C virus sequence diversity in different geographical regions: implications for virus classification. Journal of General Virology 76, 2493-2507, (1995).

1180 Majid, A., Holmes, R., Desselberger, U., Simmonds, P. & McKee, T. A. Molecular epidemiology of hepatitis C virus infection amongst intravenous drug users in rural communities. J. Med. Virol. 46, 48-51, (1995).

1181 Maggi, J., Dirks, P. & Doyle…, J. Co-amplification of MYCN and a DEAD box gene (DDX1) in primary neuroblastoma. …, (1995).

1182 Maggi, F. et al. Serological reactivity and viral genotypes in hepatitis C virus infection. J Clin Microbiol 33, 209-211, (1995).

1183 Lau, J. Y. et al. Application of six hepatitis C virus genotyping systems to sera from chronic hepatitis C patients in the United States. J Infect Dis 171, 281-289, (1995).

1184 Hofmann, H. Genotypes and virus load in patients with hepatitis C infection. Infection 23, 133-138, (1995).

1185 Gournay, J. et al. Hepatitis C virus genotypes in French blood donors. J. Med. Virol. 45, 399-404, (1995).

1186 Fretz, C. et al. HCV infection in a rural population of the Central African Republic (CAR): evidence for three additional subtypes of genotype 4. J Med Virol 47, 435-437, (1995).

1187 Davidson, F. et al. Survey of major genotypes and subtypes of hepatitis C virus using RFLP of sequences amplified from the 5′ non-coding region. Journal of General Virology 76, 1197-1204, (1995).

1188 Chemello, L., Bonetti, P., Cavalletto, L. & Talato…, F. Randomized trial comparing three different regimens of alpha-2a-interferon in chronic hepatitis C. Hepatology 22, 700-706, (1995).

1189 Chambost, H. et al. Persistent hepatitis C virus RNA replication in haemophiliacs: role of co-infection with human immunodeficiency virus. Br J Haematol 91, 703-707, (1995).

1190 Cammarota, G., Maggi, F. & Vatteroni…, M. Partial nucleotide sequencing of six subtype 2c hepatitis C viruses detected in Italy. J Clin Microbiol 33, 2781-2784, (1995).

1191 Befrits, R. et al. Chronic hepatitis C in alcoholic patients: prevalence, genotypes, and correlation to liver disease. Scand J Gastroenterol 30, 1113-1118, (1995).

1192 Ambrozaitis, A., Z Agminas, K., Balc Iūnaite, G. & Widell, A. Hepatitis C in Lithuania: incidence, prevalence, risk factors and viral genotypes. Clin Diagn Virol 4, 273-284, (1995).

1193 Altamirano, M., Delaney, A., Wong, A., Marostenmaki, J. & Pi, D. Identification of hepatitis C virus genotypes among hospitalized patients in British Columbia, Canada. J Infect Dis 171, 1034-1038, (1995).

1194 Abacioglu, Y. et al. The distribution of hepatitis C virus genotypes in Turkish patients. J Viral Hepat 2, 297-301, (1995).

1195 Woodfield, D. et al. Identification and genotyping of hepatitis C virus in injectable and oral drug users in New Zealand. Aust N Z J Med 24, 47-50, (1994).

1196 Willems, M. et al. Hepatitis C virus and its genotypes in patients suffering from chronic hepatitis C with or without a cryoglobulinemia-related syndrome. J. Med. Virol. 44, 266-271, (1994).

1197 Viazov, S. et al. Typing of hepatitis C virus isolates by \DNA\ enzyme immunoassay. J Virol Methods 48, 81 - 91, (1994).

1198 Song, P. et al. Markers of hepatitis C and B virus infections among blood donors in Ho Chi Minh City and Hanoi, Vietnam. Clin Diagn Lab Immunol 1, 413-418, (1994).

1199 Quiroga, J. A. et al. Evidence of subtype-specific antibodies to antigenic epitopes in the NS5 region of hepatitis C virus in the circulation of patients with chronic hepatitis C. Clin Diagn Lab Immunol 1, 545-551, (1994).

1200 Pujol, F. H. et al. [Usefulness of the PCR technique (polymerase chain reaction) in the follow-up of patients infected with hepatitis C virus. Preliminary communication]. G E N 48, 121-123, (1994).

1201 OKUNO, H. et al. Genotypes of hepatitis C virus in Guangxi Province, southern China. Journal of Gastroenterology and Hepatology 9, 169-171, (1994).

1202 Murphy, D., Willems, B. & Delage, G. Use of the 5' noncoding region for genotyping hepatitis C virus. J Infect Dis 169, 473-475, (1994).

1203 Mondelli, M., Cerino, A., Bono, F. & Cividini…, A. Hepatitis C virus (HCV) core serotypes in chronic HCV infection. J Clin Microbiol 32, 2523-2527, (1994).

1204 McOmish, F. et al. Geographical distribution of hepatitis C virus genotypes in blood donors: an international collaborative survey. J Clin Microbiol 32, 884-892, (1994).

1205 Masuko, K. et al. Hepatitis C virus antibodies, viral RNA and genotypes in sera from patients on maintenance haemodialysis. J Viral Hepat 1, 65-71, (1994).

1206 Mahaney, K. et al. Genotypic analysis of hepatitis C virus in American patients. Hepatology 20, 1405-1411, (1994).

1207 Luengrojanakul, P. et al. Hepatitis C virus infection in patients with chronic liver disease or chronic renal failure and blood donors in Thailand. J. Med. Virol. 44, 287-292, (1994).

1208 Lee, A. et al. Partial sequence analysis of the hepatitis C viral genome in Singapore patients. Biochem Biophys Res Commun 199, 37-40, (1994).

1209 Ichimura, H. et al. Hepatitis C virus genotypes, reactivity to recombinant immunoblot assay 2 antigens and liver disease. J. Med. Virol. 43, 212-215, (1994).

1210 Hadiwandowo, S. et al. Hepatitis B virus subtypes and hepatitis C virus genotypes in patients with chronic liver disease or on maintenance hemodialysis in Indonesia. J. Med. Virol. 43, 182-186, (1994).

1211 Du, D. et al. Heterogeneity of Hepatitis C Virus Genotypes in France. Journal of General Virology 75, 1063-1070, (1994).

1212 Driesel, G. et al. Hepatitis C virus (HCV) genotype distribution in German isolates: studies on the sequence variability in the E2 and NS5 region. Arch Virol 139, 379-388, (1994).

1213 Arico, M., Maggiore, G., Silini, E. & Bono…, F. Hepatitis C virus infection in children treated for acute lymphoblastic leukemia. Blood 84, 2919-2922, (1994).

1214 Andonov, A. & Chaudhary, R. K. Genotyping of Canadian hepatitis C virus isolates by PCR. J Clin Microbiol 32, 2031-2034, (1994).

1215 Alonso, C. et al. Serological responses to different genotypes of hepatitis C virus in France. J Clin Microbiol 32, 211-212, (1994).

1216 Wang, Y. et al. Prevalence, genotypes, and an isolate (HC-C2) of hepatitis C virus in Chinese patients with liver disease. J. Med. Virol. 40, 254-260, (1993).

1217 Silini, E., Bono, F., Cerino, A. & Piazza…, V. Virological features of hepatitis C virus infection in hemodialysis patients. J Clin Microbiol 31, 2913-2917, (1993).
